# Supplementary figures and images for: Proteolytic cleavage of G3BP1 by calpain 1 couples NMDAR activation to mTOR-dependent local translation (part 1 of 2)
Source: EMBO Rep. 2026 Apr 4;27(10):2749–71. doi: 10.1038/s44319-026-00766-9 (PMC13219515; doi:10.1038/s44319-026-00766-9)

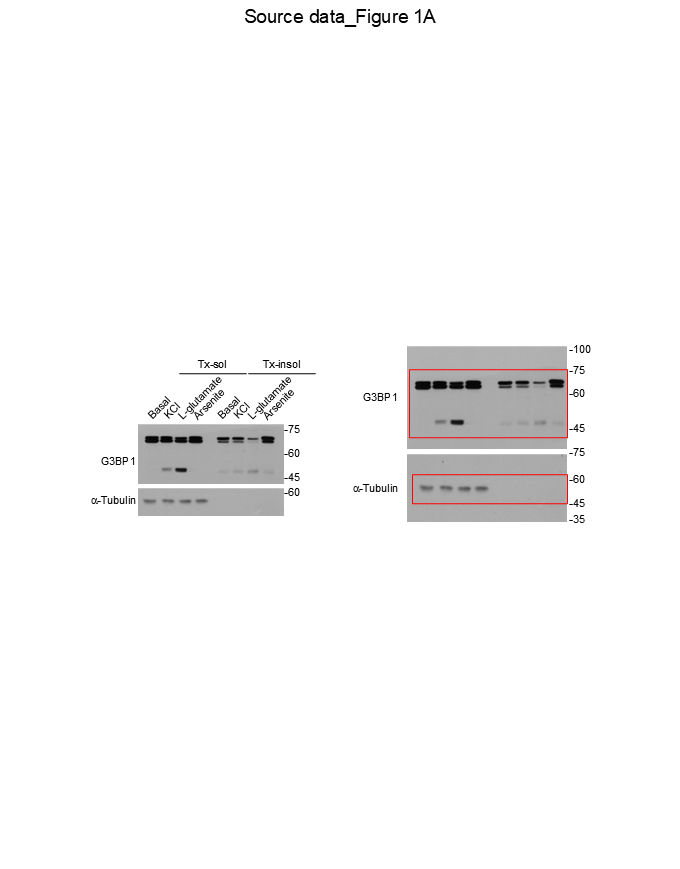

Supplement: Supplementary file 2 — Source data Fig. 1 [file 44319_2026_766_MOESM2_ESM.zip › 1A/Figure1A_Blots.TIF]

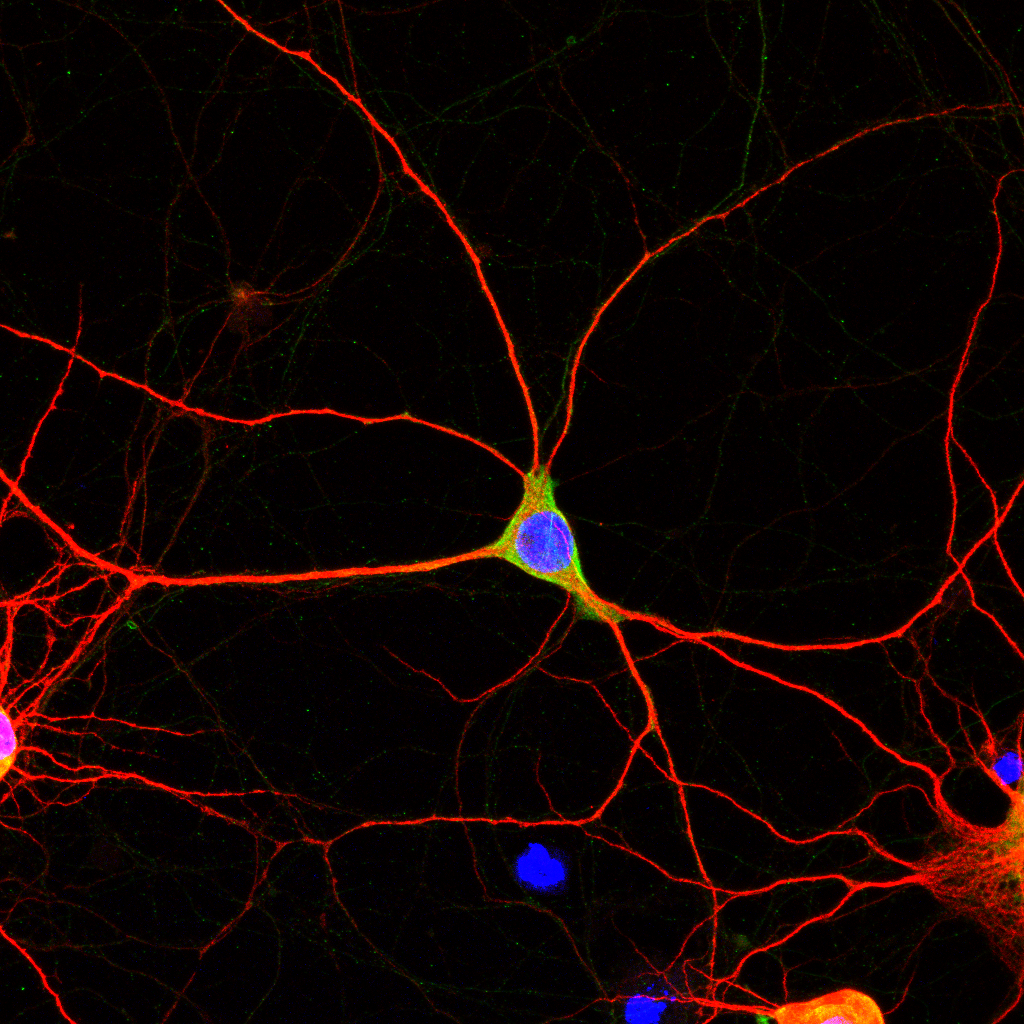

Supplement: Supplementary file 2 — Source data Fig. 1 [file 44319_2026_766_MOESM2_ESM.zip › 1C/G3BP1_MAP2_Tau_Fig1002_1. Basal003_Processed001.tif]

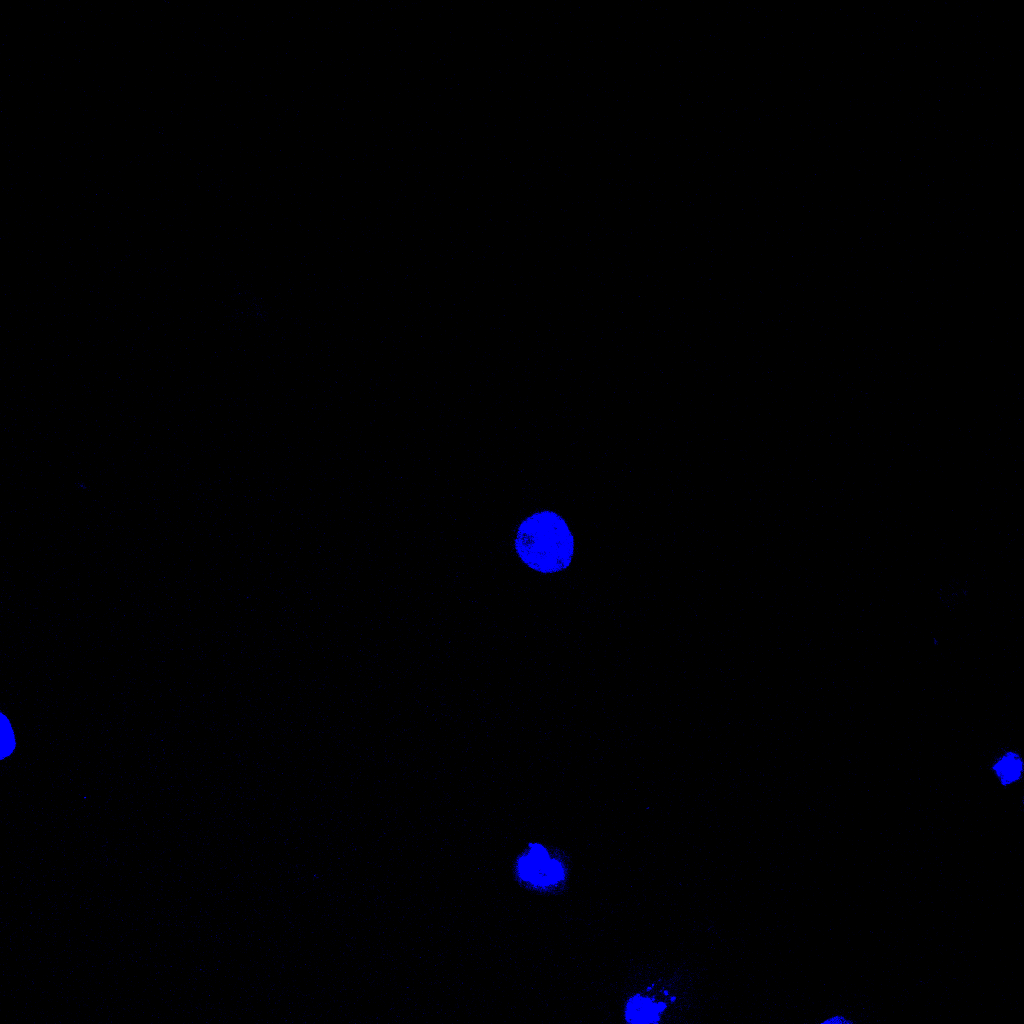

Supplement: Supplementary file 2 — Source data Fig. 1 [file 44319_2026_766_MOESM2_ESM.zip › 1C/G3BP1_MAP2_Tau_Fig1002_1. Basal003_Processed001_ch00.tif]

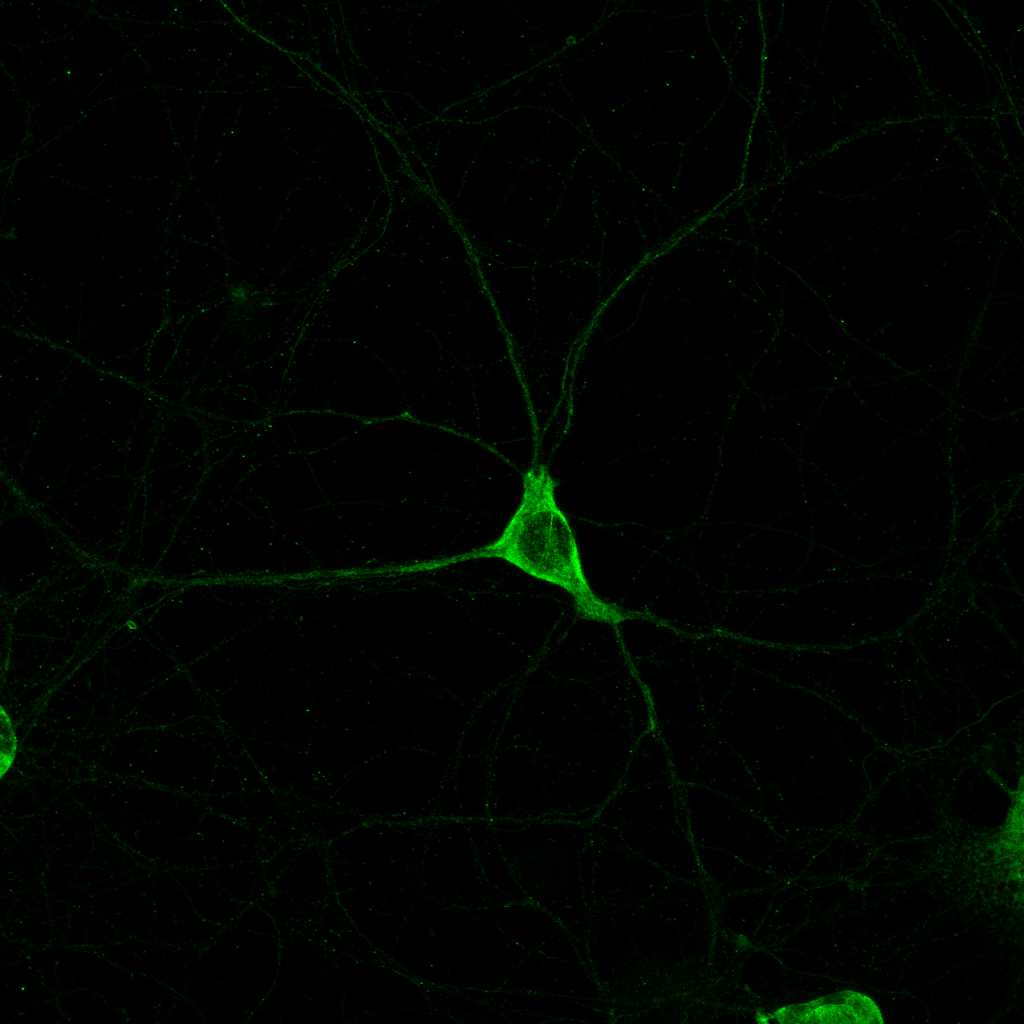

Supplement: Supplementary file 2 — Source data Fig. 1 [file 44319_2026_766_MOESM2_ESM.zip › 1C/G3BP1_MAP2_Tau_Fig1002_1. Basal003_Processed001_ch01.tif]

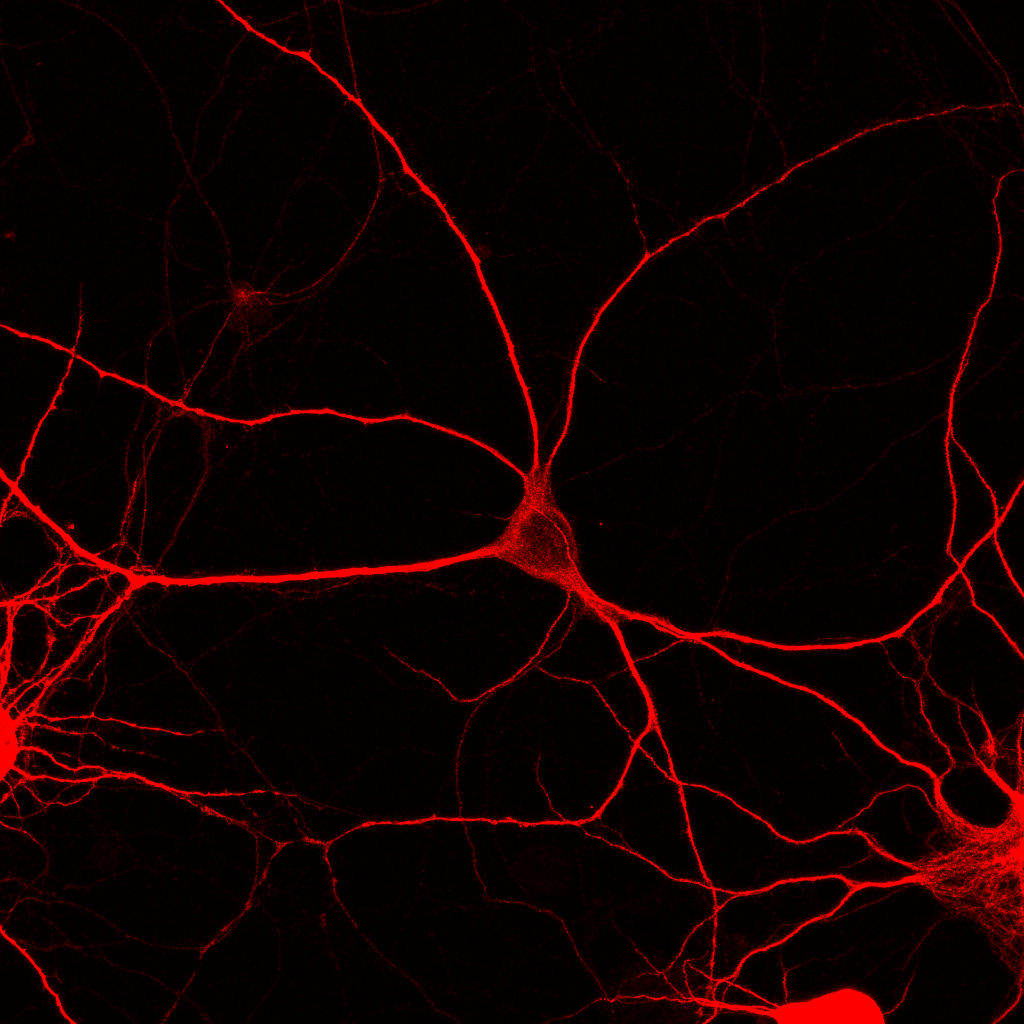

Supplement: Supplementary file 2 — Source data Fig. 1 [file 44319_2026_766_MOESM2_ESM.zip › 1C/G3BP1_MAP2_Tau_Fig1002_1. Basal003_Processed001_ch02.tif]

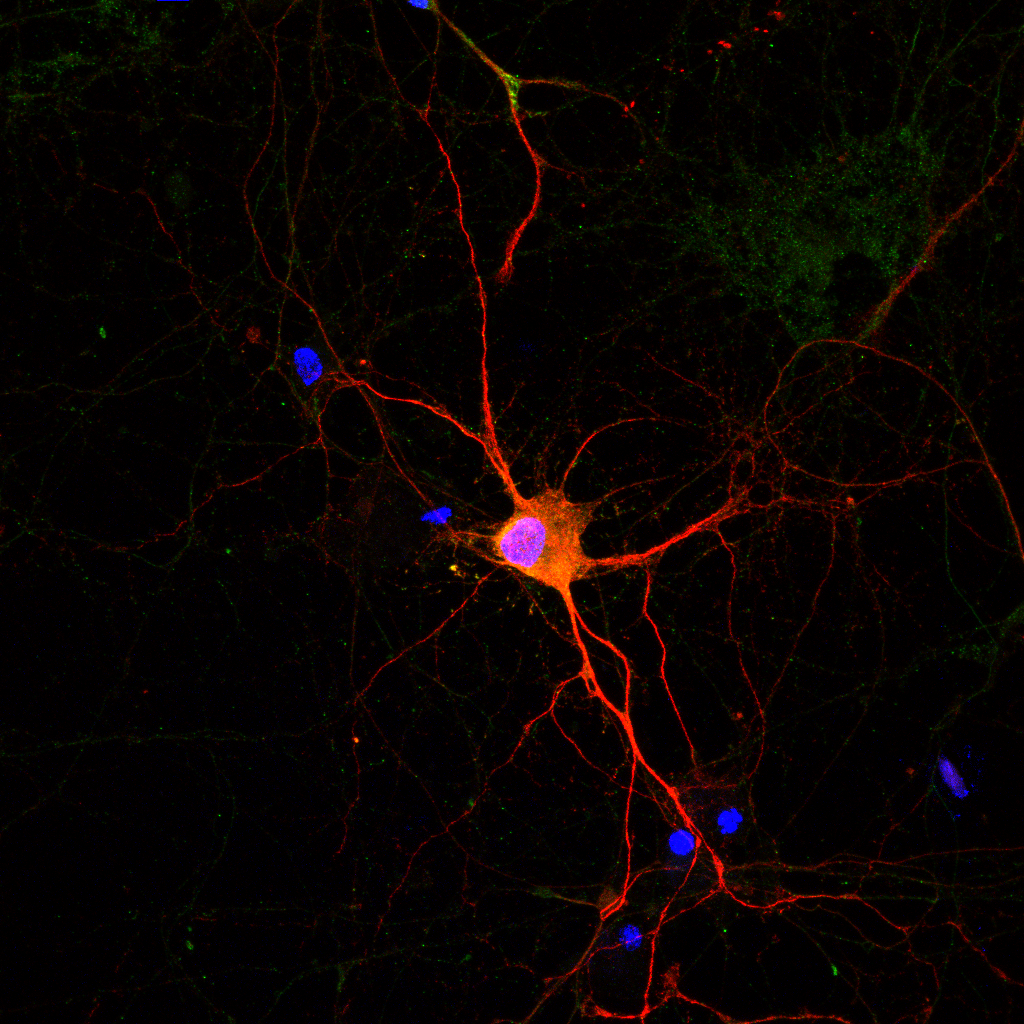

Supplement: Supplementary file 2 — Source data Fig. 1 [file 44319_2026_766_MOESM2_ESM.zip › 1C/G3BP1_MAP2_Tau_Fig1002_3. L-glu004_Processed001.tif]

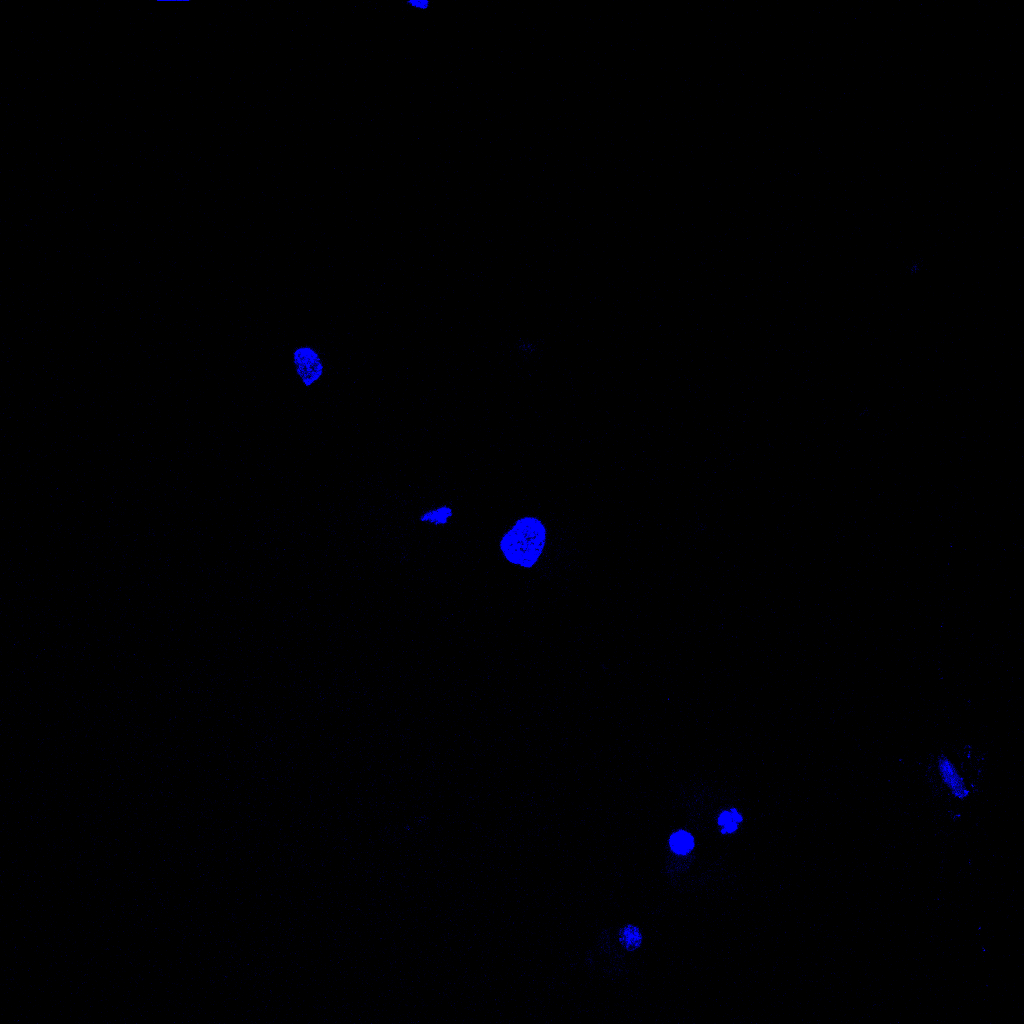

Supplement: Supplementary file 2 — Source data Fig. 1 [file 44319_2026_766_MOESM2_ESM.zip › 1C/G3BP1_MAP2_Tau_Fig1002_3. L-glu004_Processed001_ch00.tif]

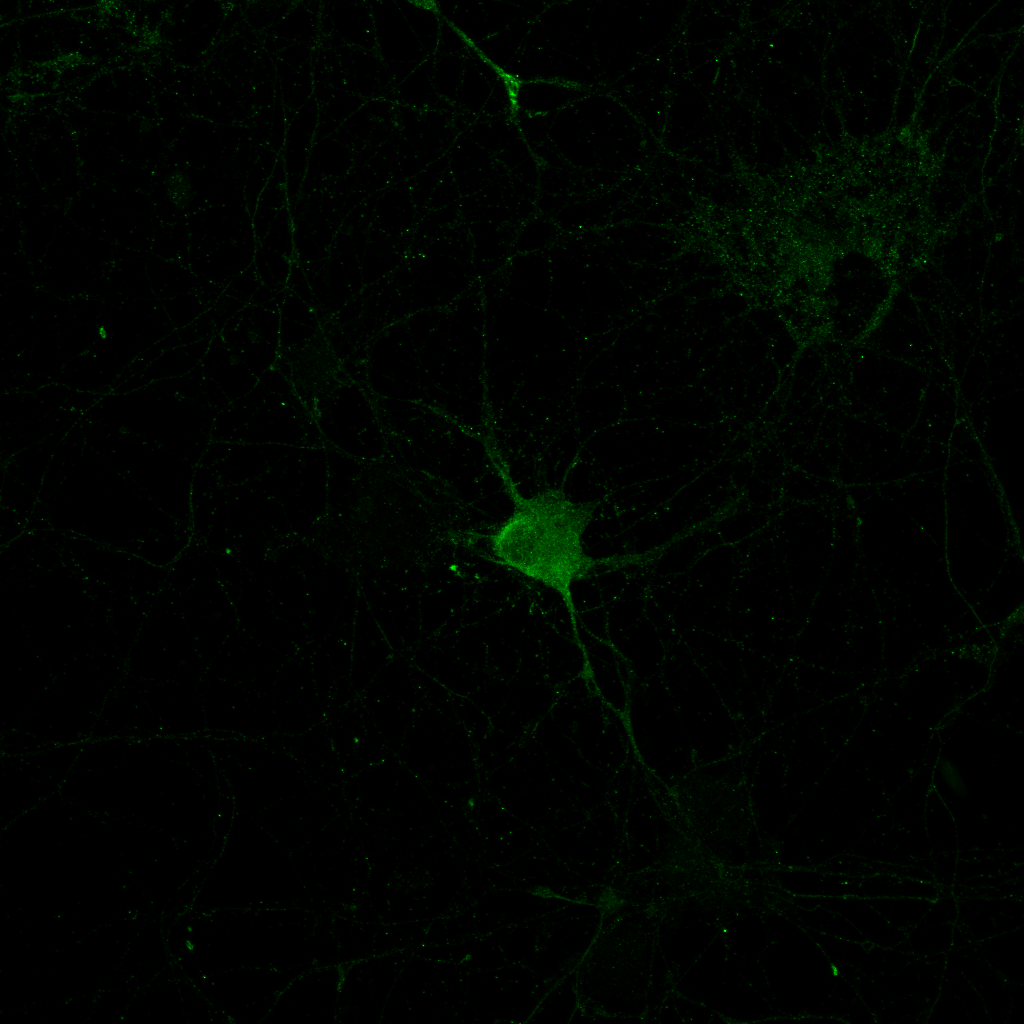

Supplement: Supplementary file 2 — Source data Fig. 1 [file 44319_2026_766_MOESM2_ESM.zip › 1C/G3BP1_MAP2_Tau_Fig1002_3. L-glu004_Processed001_ch01.tif]

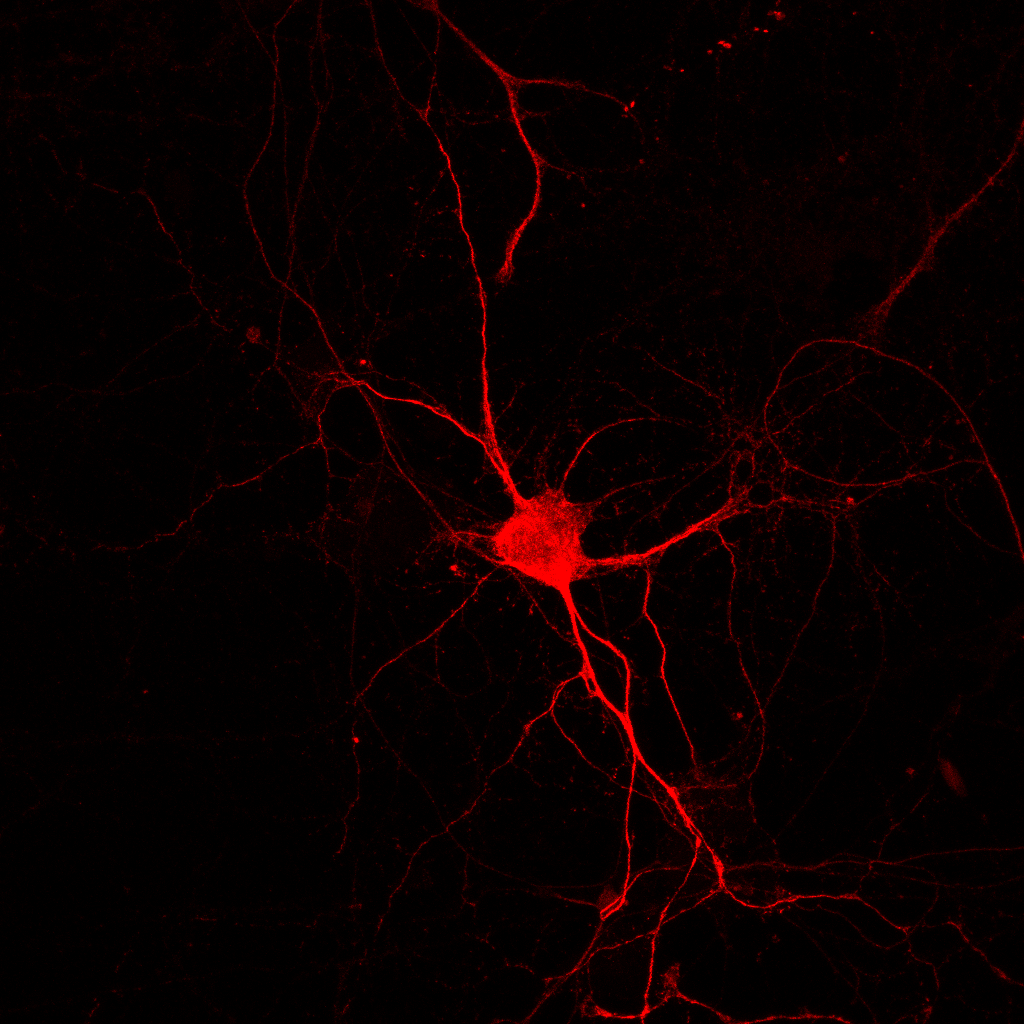

Supplement: Supplementary file 2 — Source data Fig. 1 [file 44319_2026_766_MOESM2_ESM.zip › 1C/G3BP1_MAP2_Tau_Fig1002_3. L-glu004_Processed001_ch02.tif]

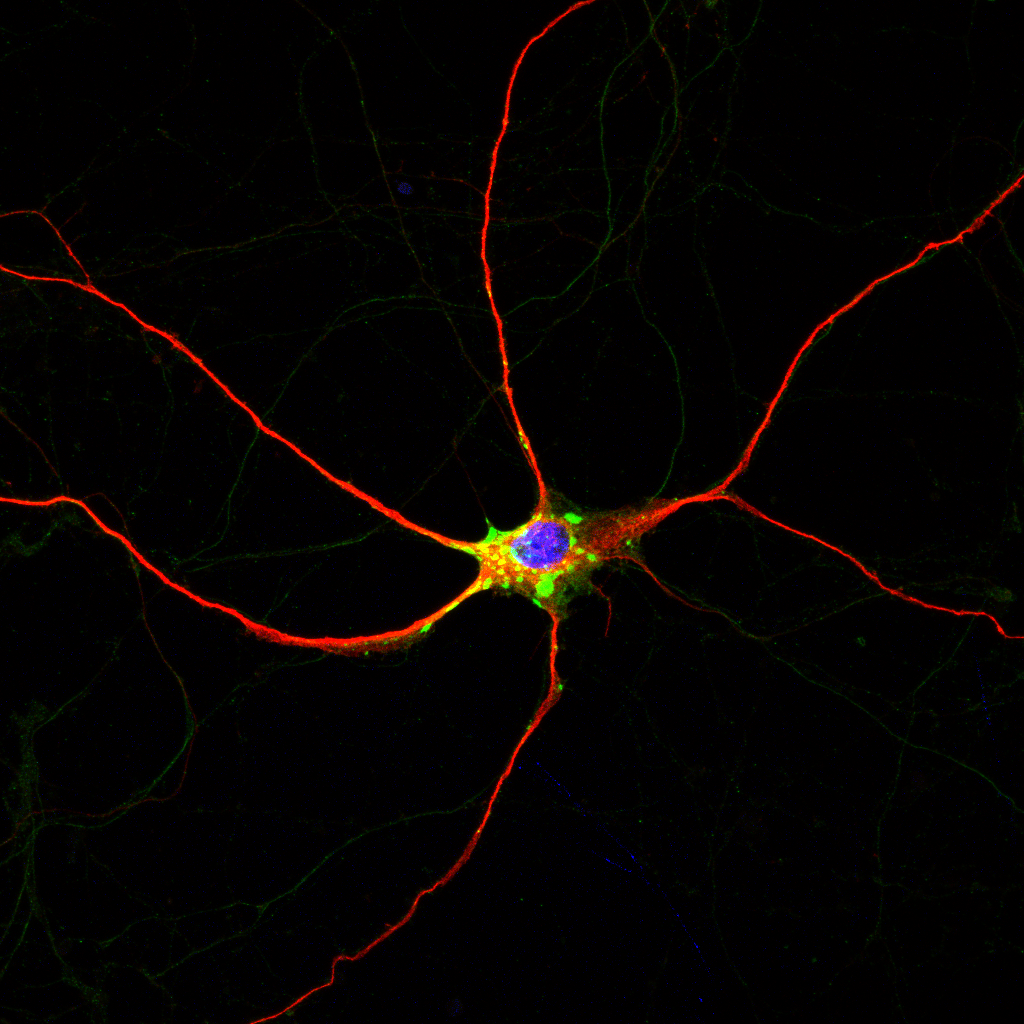

Supplement: Supplementary file 2 — Source data Fig. 1 [file 44319_2026_766_MOESM2_ESM.zip › 1C/G3BP1_MAP2_Tau_Fig1002_4. S.A004_Processed001.tif]

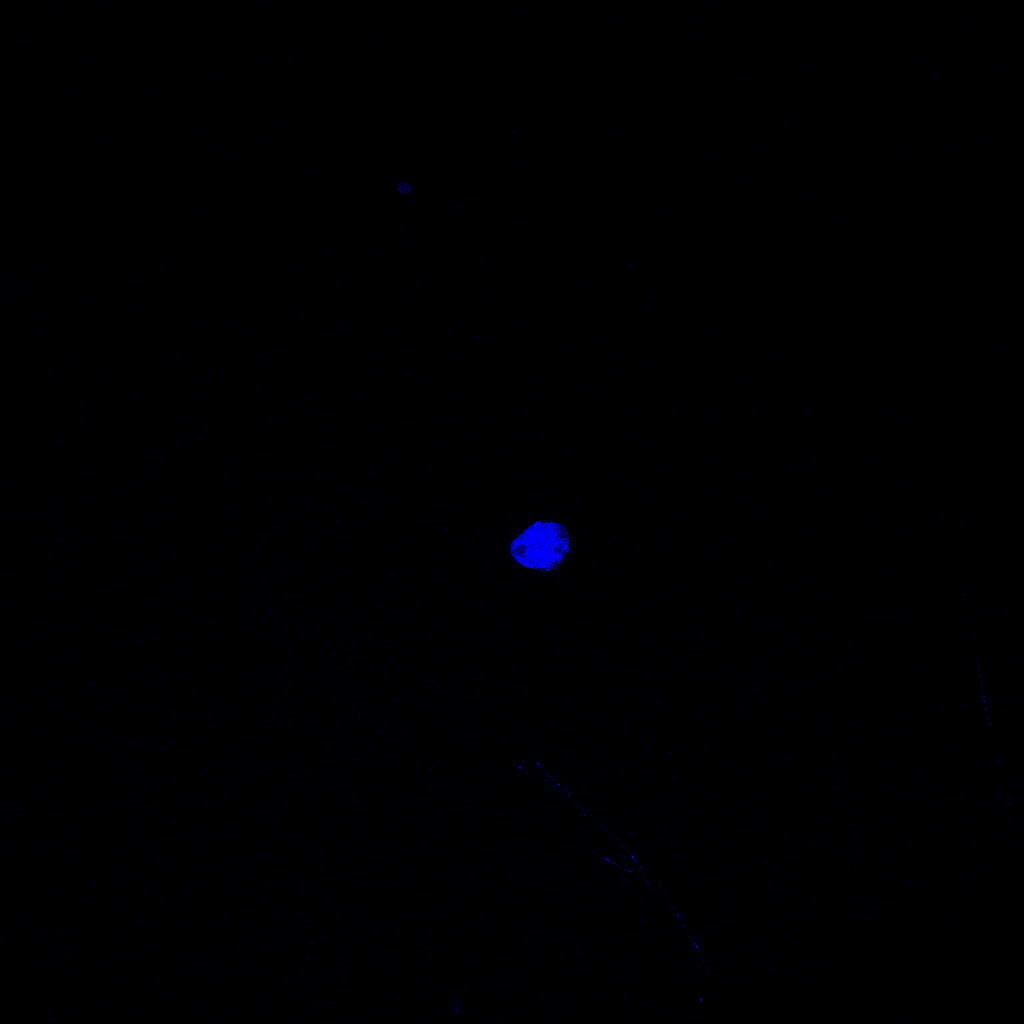

Supplement: Supplementary file 2 — Source data Fig. 1 [file 44319_2026_766_MOESM2_ESM.zip › 1C/G3BP1_MAP2_Tau_Fig1002_4. S.A004_Processed001_ch00.tif]

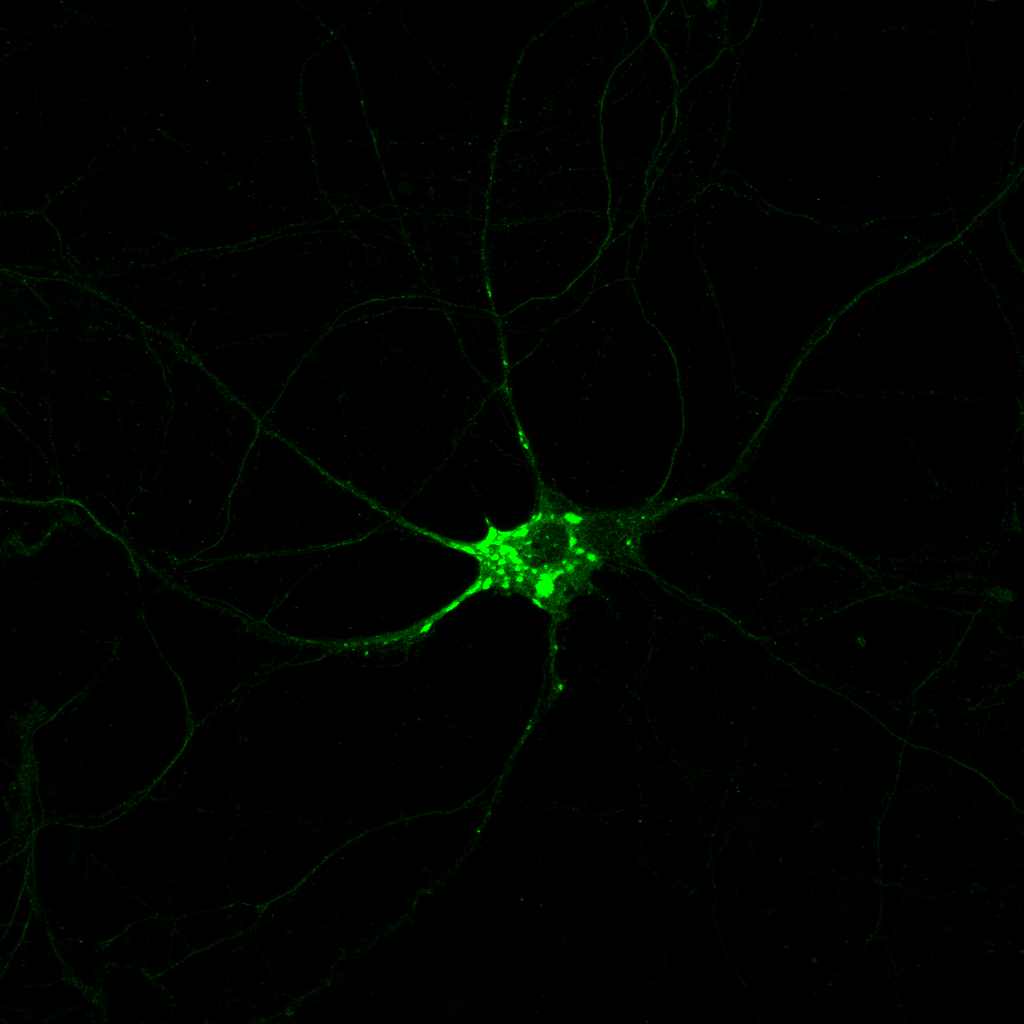

Supplement: Supplementary file 2 — Source data Fig. 1 [file 44319_2026_766_MOESM2_ESM.zip › 1C/G3BP1_MAP2_Tau_Fig1002_4. S.A004_Processed001_ch01.tif]

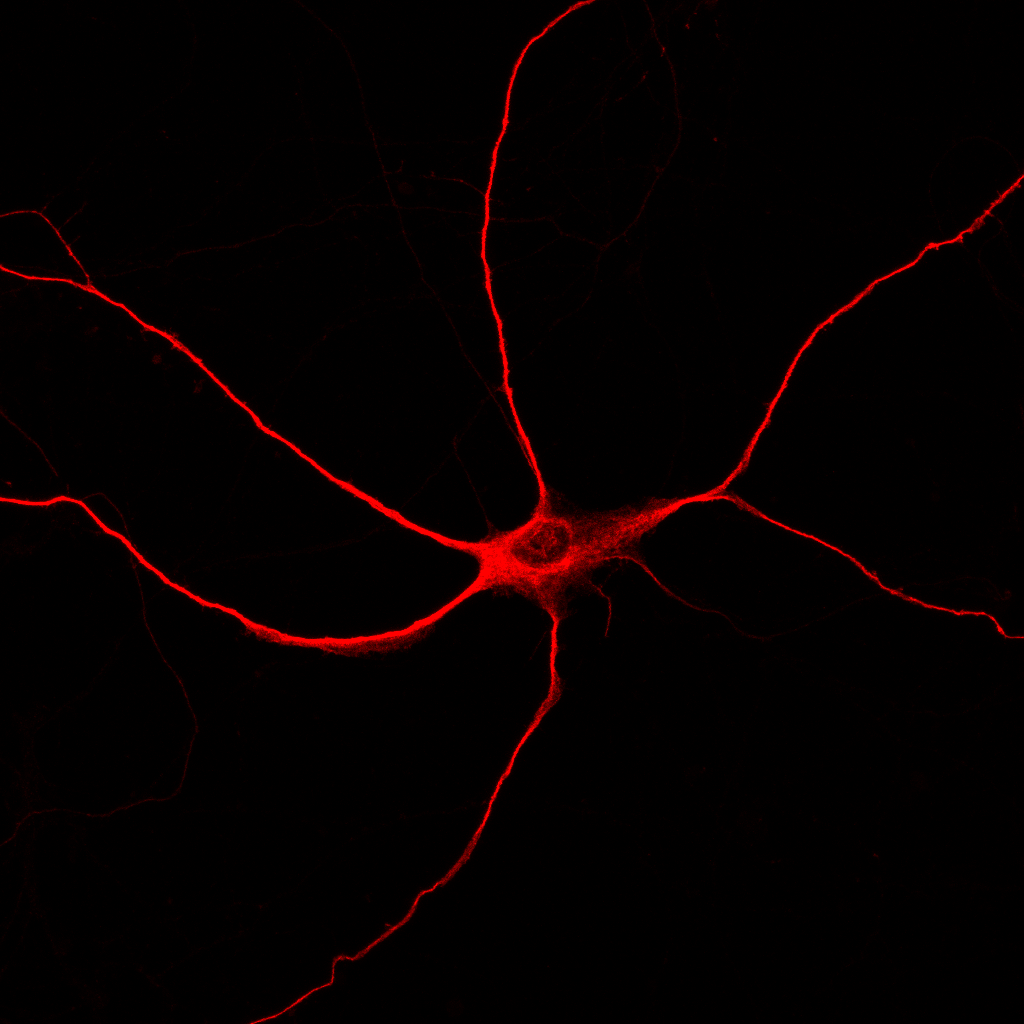

Supplement: Supplementary file 2 — Source data Fig. 1 [file 44319_2026_766_MOESM2_ESM.zip › 1C/G3BP1_MAP2_Tau_Fig1002_4. S.A004_Processed001_ch02.tif]

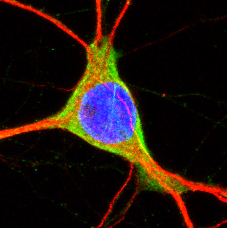

Supplement: Supplementary file 2 — Source data Fig. 1 [file 44319_2026_766_MOESM2_ESM.zip › 1C/Inset, Cell body/1_0000_G3BP1_MAP2_Tau_Fig1002_1. Basal003_Processed001.tif.tif]

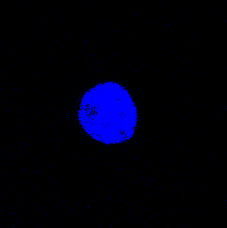

Supplement: Supplementary file 2 — Source data Fig. 1 [file 44319_2026_766_MOESM2_ESM.zip › 1C/Inset, Cell body/1_0001_G3BP1_MAP2_Tau_Fig1002_1. Basal003_Processed001_ch00.tif.tif]

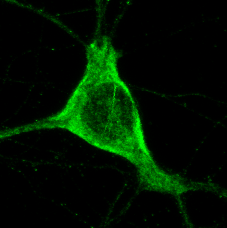

Supplement: Supplementary file 2 — Source data Fig. 1 [file 44319_2026_766_MOESM2_ESM.zip › 1C/Inset, Cell body/1_0002_G3BP1_MAP2_Tau_Fig1002_1. Basal003_Processed001_ch01.tif.tif]

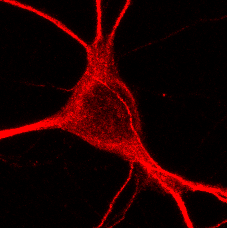

Supplement: Supplementary file 2 — Source data Fig. 1 [file 44319_2026_766_MOESM2_ESM.zip › 1C/Inset, Cell body/1_0003_G3BP1_MAP2_Tau_Fig1002_1. Basal003_Processed001_ch02.tif.tif]

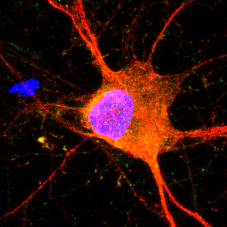

Supplement: Supplementary file 2 — Source data Fig. 1 [file 44319_2026_766_MOESM2_ESM.zip › 1C/Inset, Cell body/3_0000_G3BP1_MAP2_Tau_Fig1002_3. L-glu004_Processed001.tif.tif]

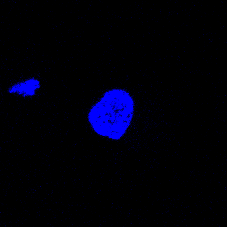

Supplement: Supplementary file 2 — Source data Fig. 1 [file 44319_2026_766_MOESM2_ESM.zip › 1C/Inset, Cell body/3_0001_G3BP1_MAP2_Tau_Fig1002_3. L-glu004_Processed001_ch00.tif.tif]

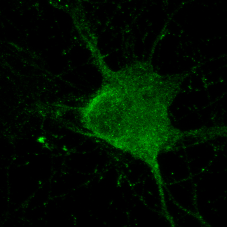

Supplement: Supplementary file 2 — Source data Fig. 1 [file 44319_2026_766_MOESM2_ESM.zip › 1C/Inset, Cell body/3_0002_G3BP1_MAP2_Tau_Fig1002_3. L-glu004_Processed001_ch01.tif.tif]

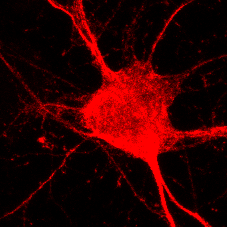

Supplement: Supplementary file 2 — Source data Fig. 1 [file 44319_2026_766_MOESM2_ESM.zip › 1C/Inset, Cell body/3_0003_G3BP1_MAP2_Tau_Fig1002_3. L-glu004_Processed001_ch02.tif.tif]

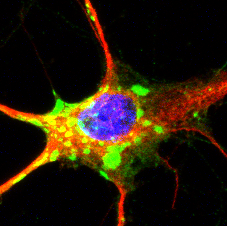

Supplement: Supplementary file 2 — Source data Fig. 1 [file 44319_2026_766_MOESM2_ESM.zip › 1C/Inset, Cell body/4_0000_G3BP1_MAP2_Tau_Fig1002_4. S.A004_Processed001.tif.tif]

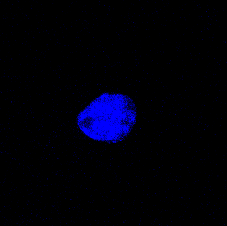

Supplement: Supplementary file 2 — Source data Fig. 1 [file 44319_2026_766_MOESM2_ESM.zip › 1C/Inset, Cell body/4_0001_G3BP1_MAP2_Tau_Fig1002_4. S.A004_Processed001_ch00.tif.tif]

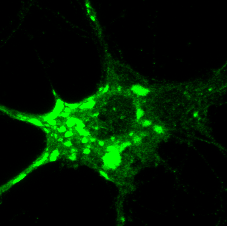

Supplement: Supplementary file 2 — Source data Fig. 1 [file 44319_2026_766_MOESM2_ESM.zip › 1C/Inset, Cell body/4_0002_G3BP1_MAP2_Tau_Fig1002_4. S.A004_Processed001_ch01.tif.tif]

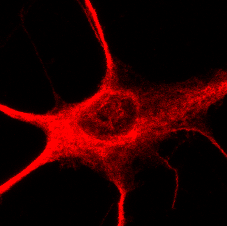

Supplement: Supplementary file 2 — Source data Fig. 1 [file 44319_2026_766_MOESM2_ESM.zip › 1C/Inset, Cell body/4_0003_G3BP1_MAP2_Tau_Fig1002_4. S.A004_Processed001_ch02.tif.tif]

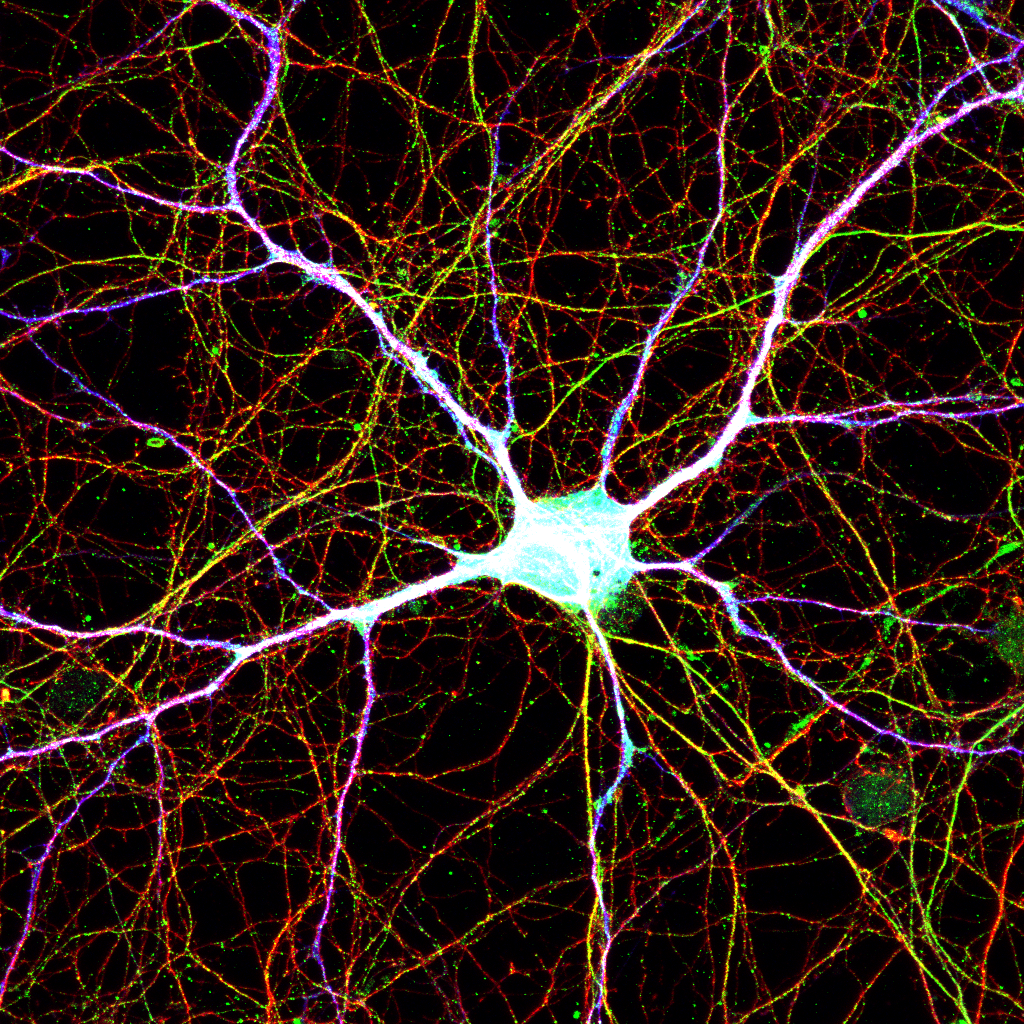

Supplement: Supplementary file 2 — Source data Fig. 1 [file 44319_2026_766_MOESM2_ESM.zip › 1D/G3BP1_MAP2_Tau_Fig1002_1. Basal001_Processed001.tif]

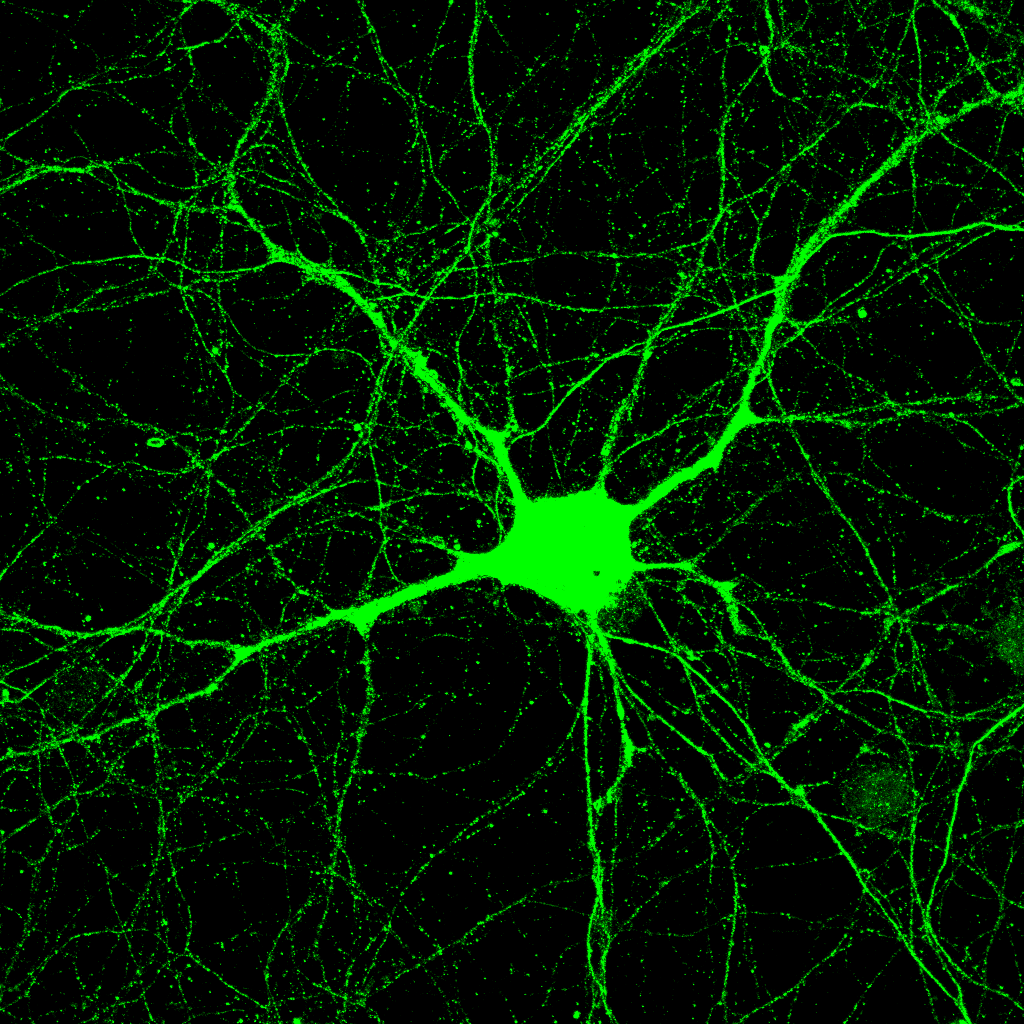

Supplement: Supplementary file 2 — Source data Fig. 1 [file 44319_2026_766_MOESM2_ESM.zip › 1D/G3BP1_MAP2_Tau_Fig1002_1. Basal001_Processed001_ch01.tif]

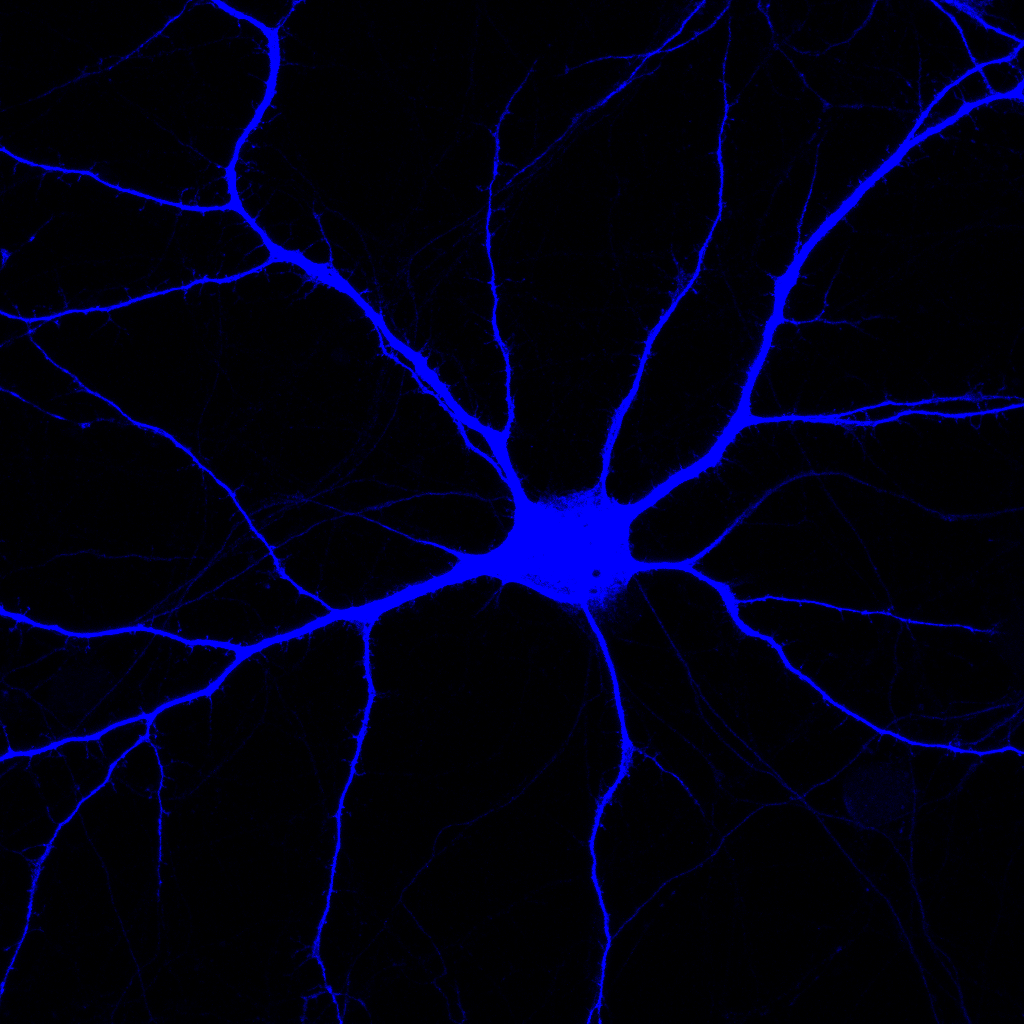

Supplement: Supplementary file 2 — Source data Fig. 1 [file 44319_2026_766_MOESM2_ESM.zip › 1D/G3BP1_MAP2_Tau_Fig1002_1. Basal001_Processed001_ch02.tif]

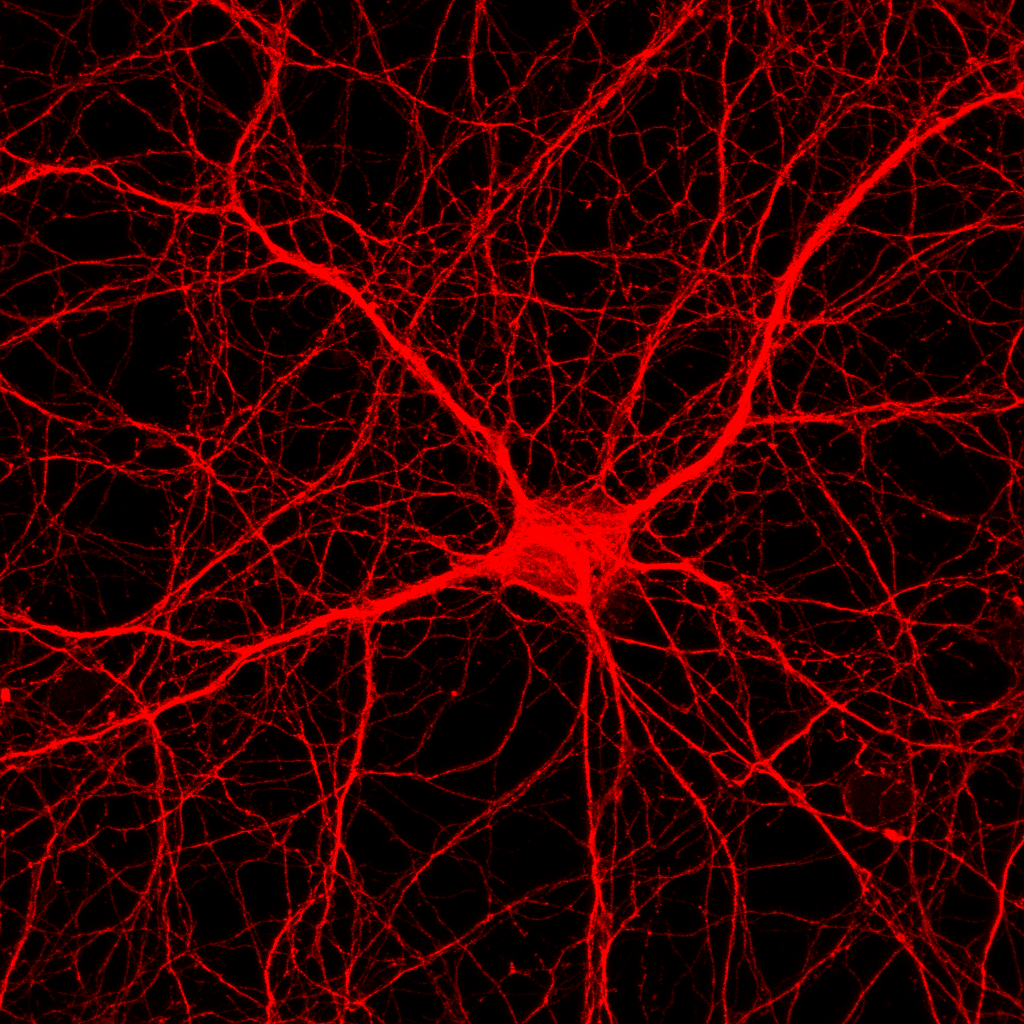

Supplement: Supplementary file 2 — Source data Fig. 1 [file 44319_2026_766_MOESM2_ESM.zip › 1D/G3BP1_MAP2_Tau_Fig1002_1. Basal001_Processed001_ch03.tif]

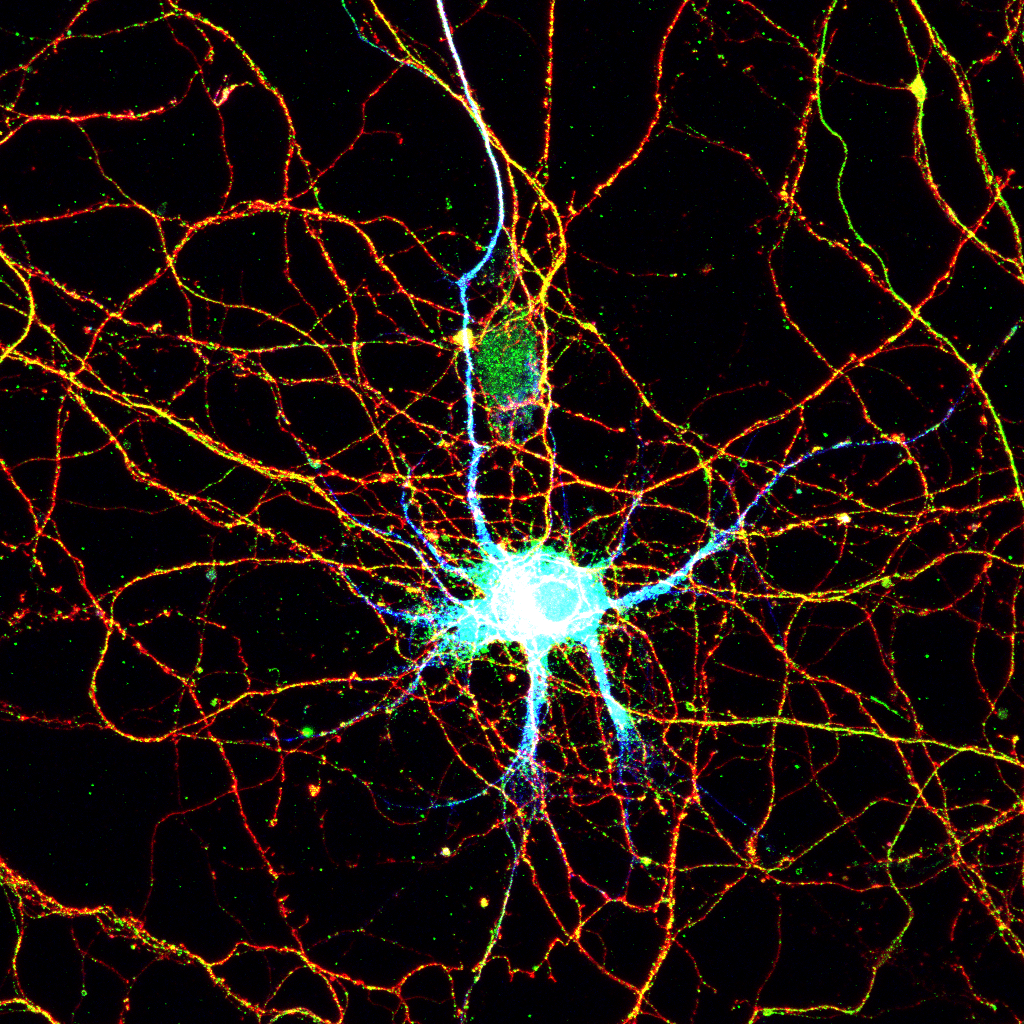

Supplement: Supplementary file 2 — Source data Fig. 1 [file 44319_2026_766_MOESM2_ESM.zip › 1D/G3BP1_MAP2_Tau_Fig1002_3. L-glu003_Processed001.tif]

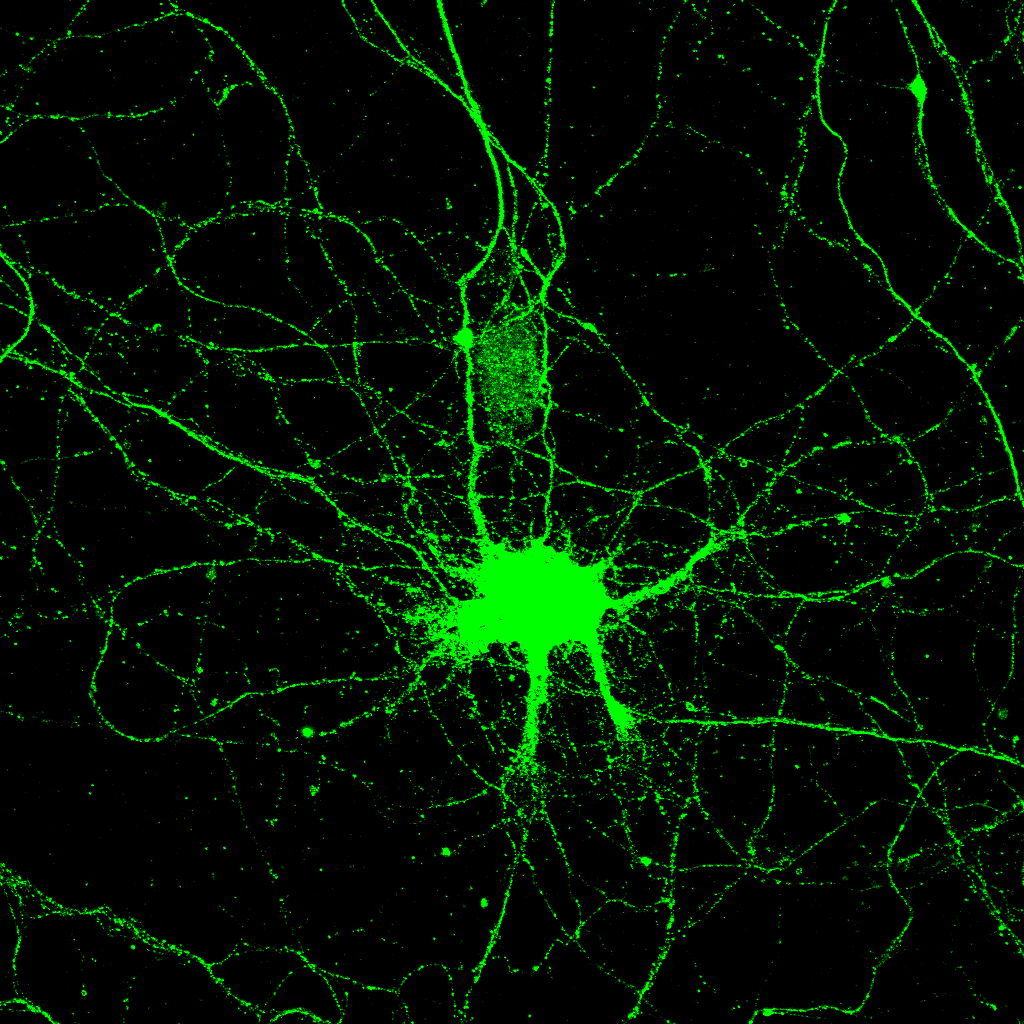

Supplement: Supplementary file 2 — Source data Fig. 1 [file 44319_2026_766_MOESM2_ESM.zip › 1D/G3BP1_MAP2_Tau_Fig1002_3. L-glu003_Processed001_ch01.tif]

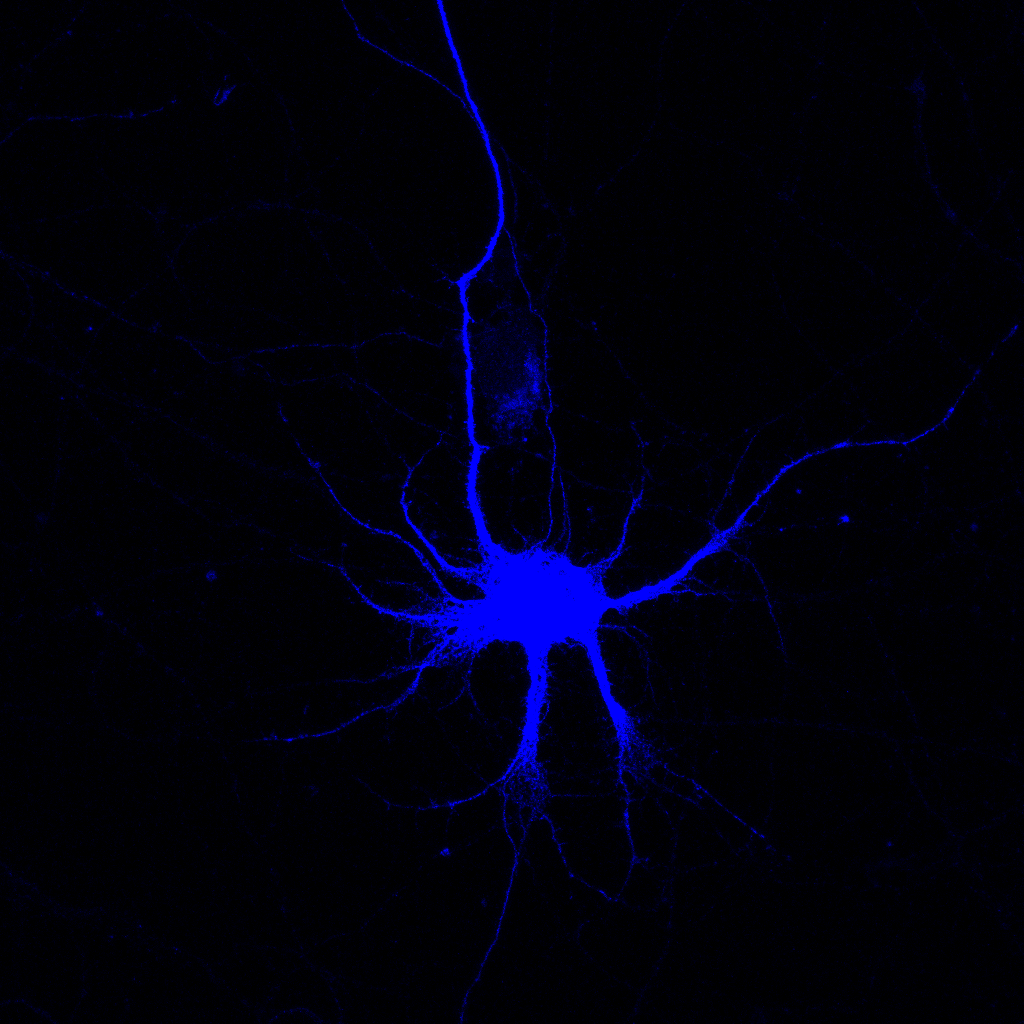

Supplement: Supplementary file 2 — Source data Fig. 1 [file 44319_2026_766_MOESM2_ESM.zip › 1D/G3BP1_MAP2_Tau_Fig1002_3. L-glu003_Processed001_ch02.tif]

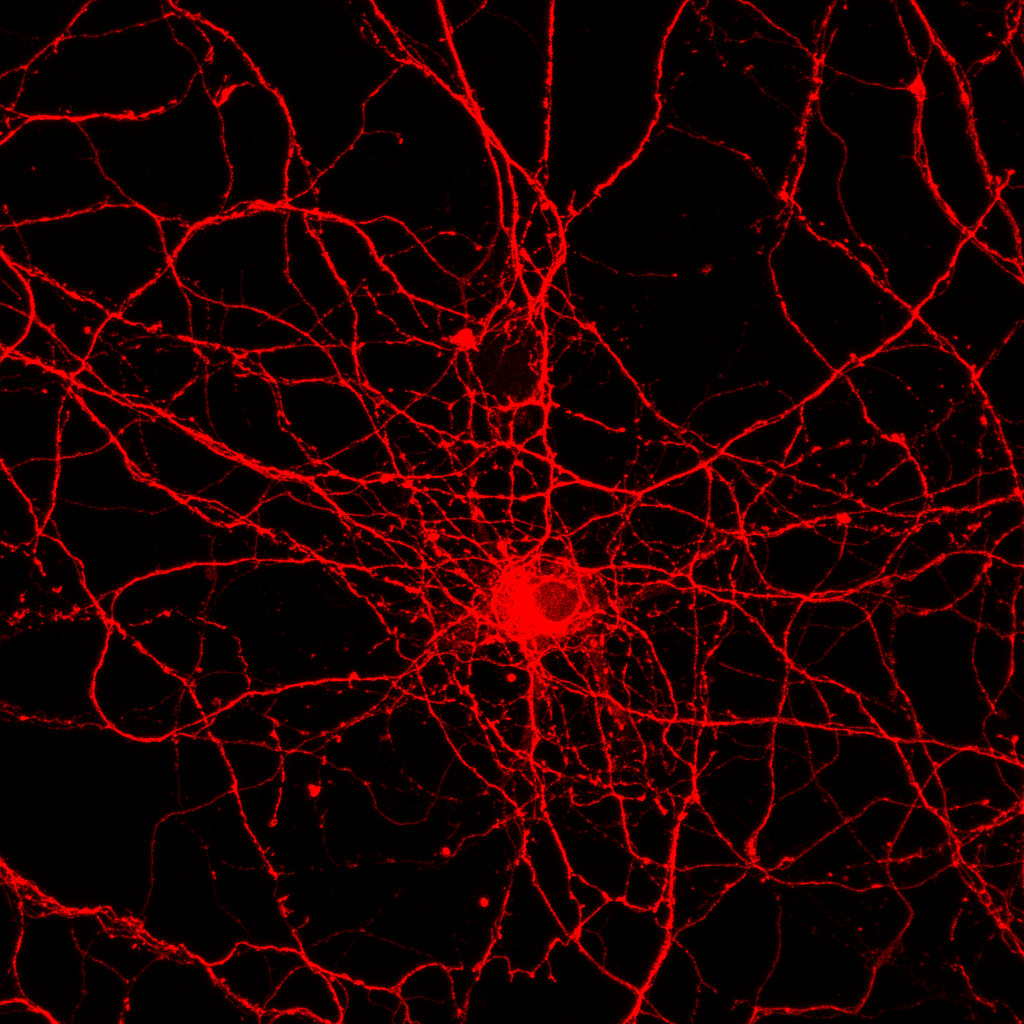

Supplement: Supplementary file 2 — Source data Fig. 1 [file 44319_2026_766_MOESM2_ESM.zip › 1D/G3BP1_MAP2_Tau_Fig1002_3. L-glu003_Processed001_ch03.tif]

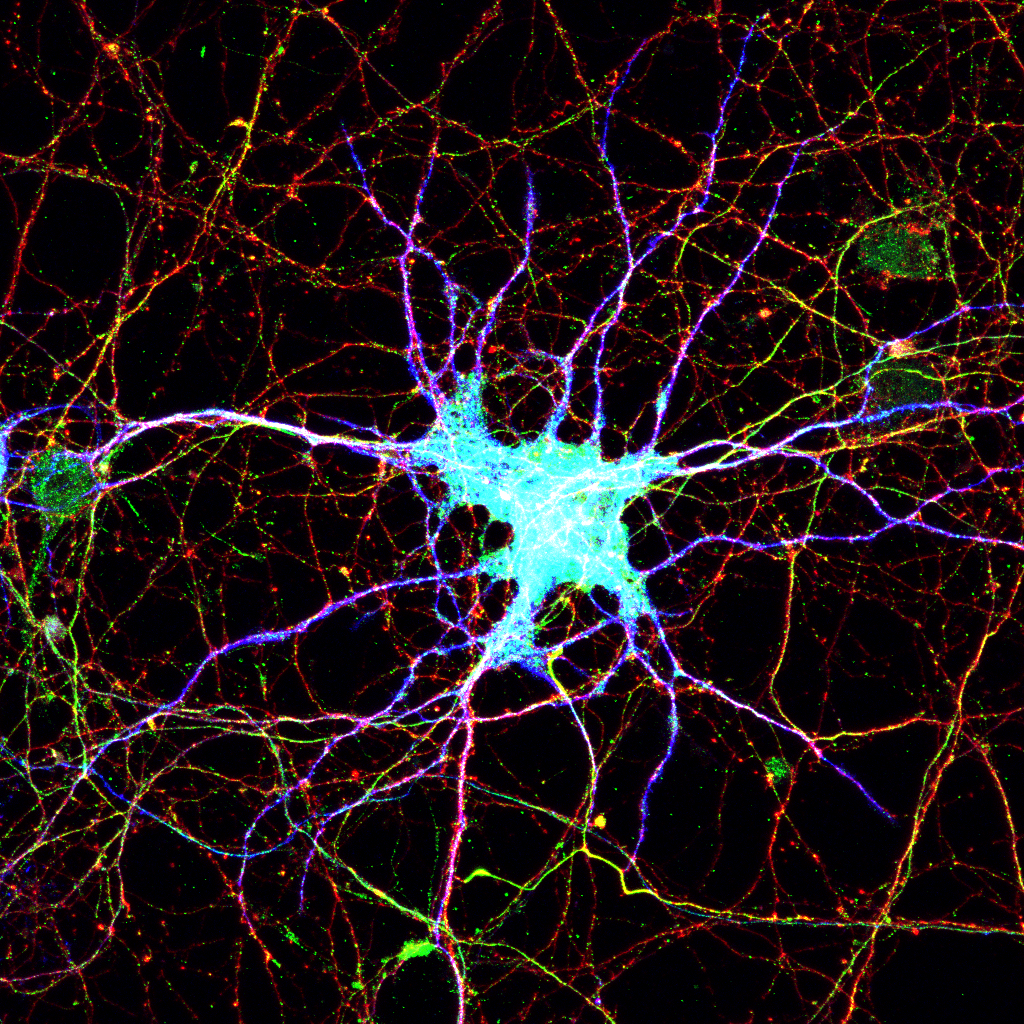

Supplement: Supplementary file 2 — Source data Fig. 1 [file 44319_2026_766_MOESM2_ESM.zip › 1D/G3BP1_MAP2_Tau_Fig1002_4. S.A003_Processed001.tif]

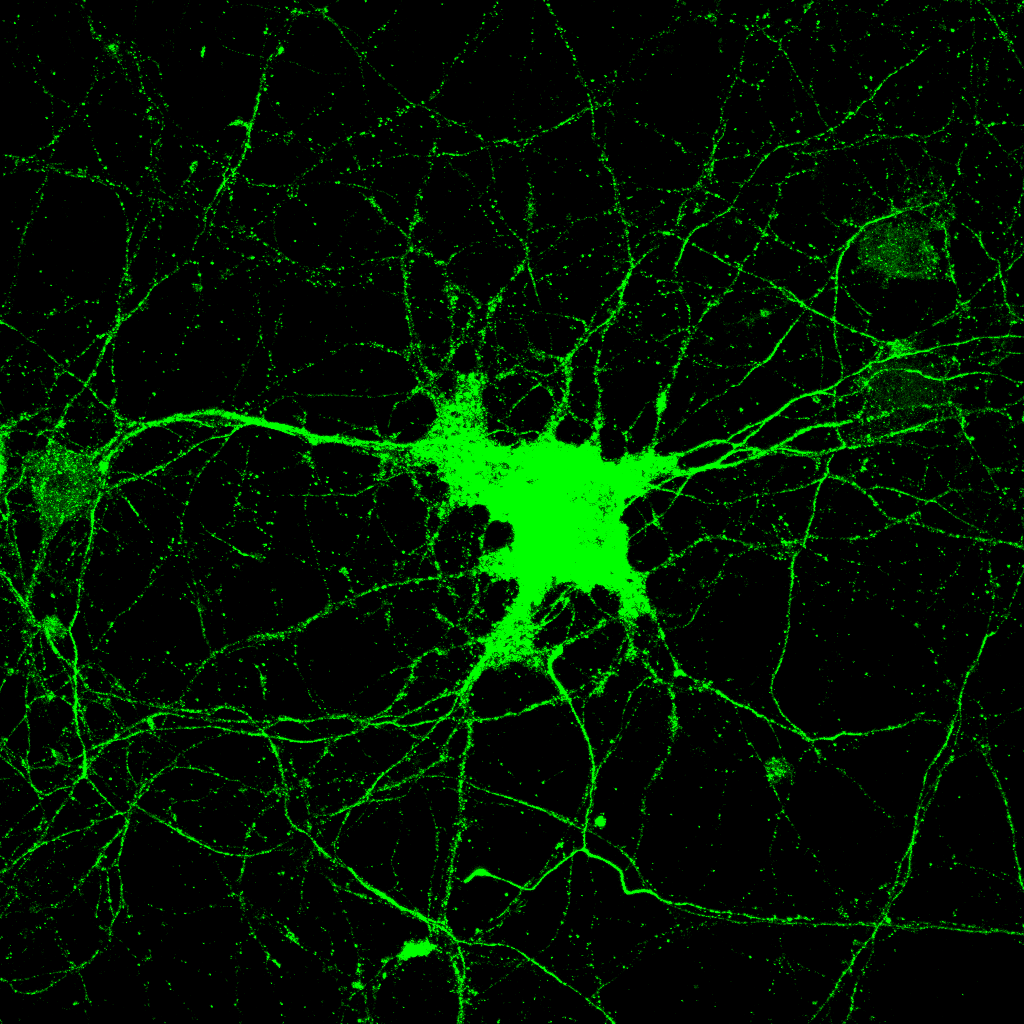

Supplement: Supplementary file 2 — Source data Fig. 1 [file 44319_2026_766_MOESM2_ESM.zip › 1D/G3BP1_MAP2_Tau_Fig1002_4. S.A003_Processed001_ch01.tif]

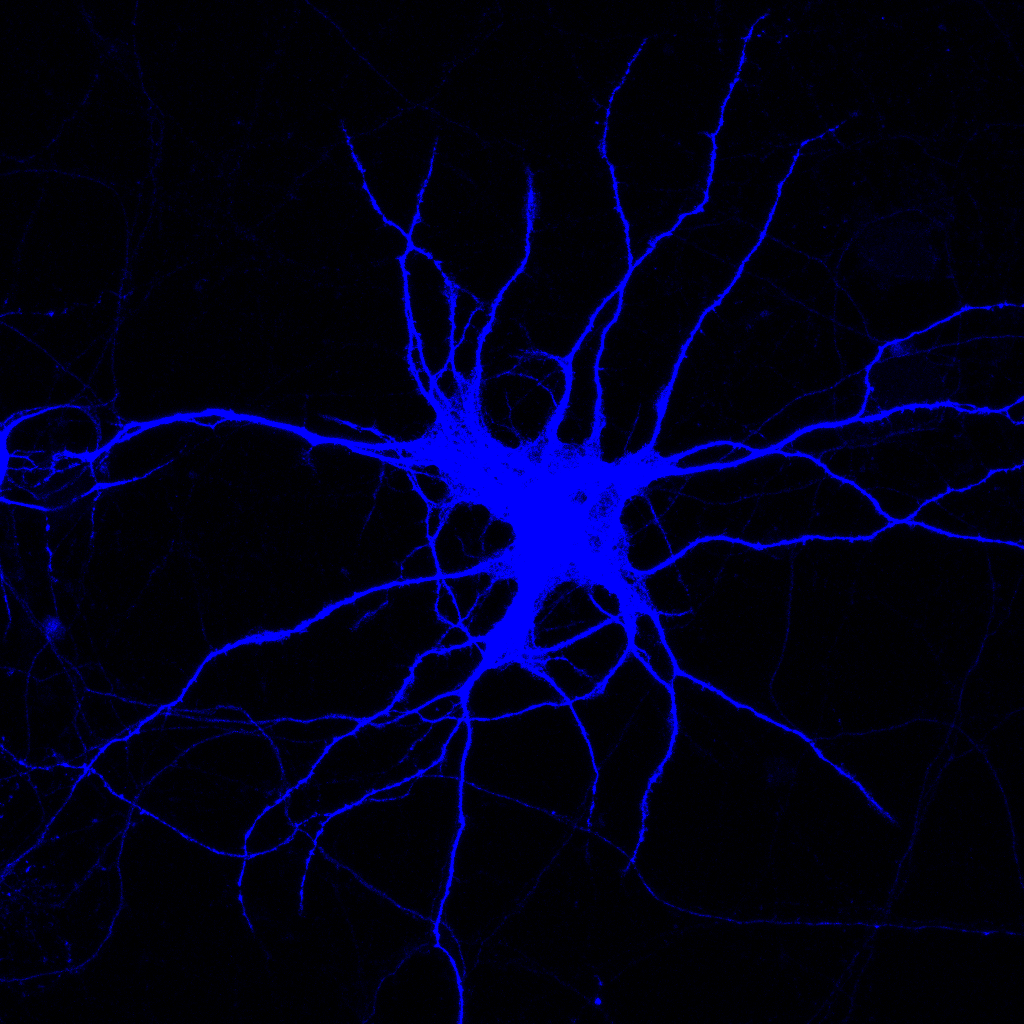

Supplement: Supplementary file 2 — Source data Fig. 1 [file 44319_2026_766_MOESM2_ESM.zip › 1D/G3BP1_MAP2_Tau_Fig1002_4. S.A003_Processed001_ch02.tif]

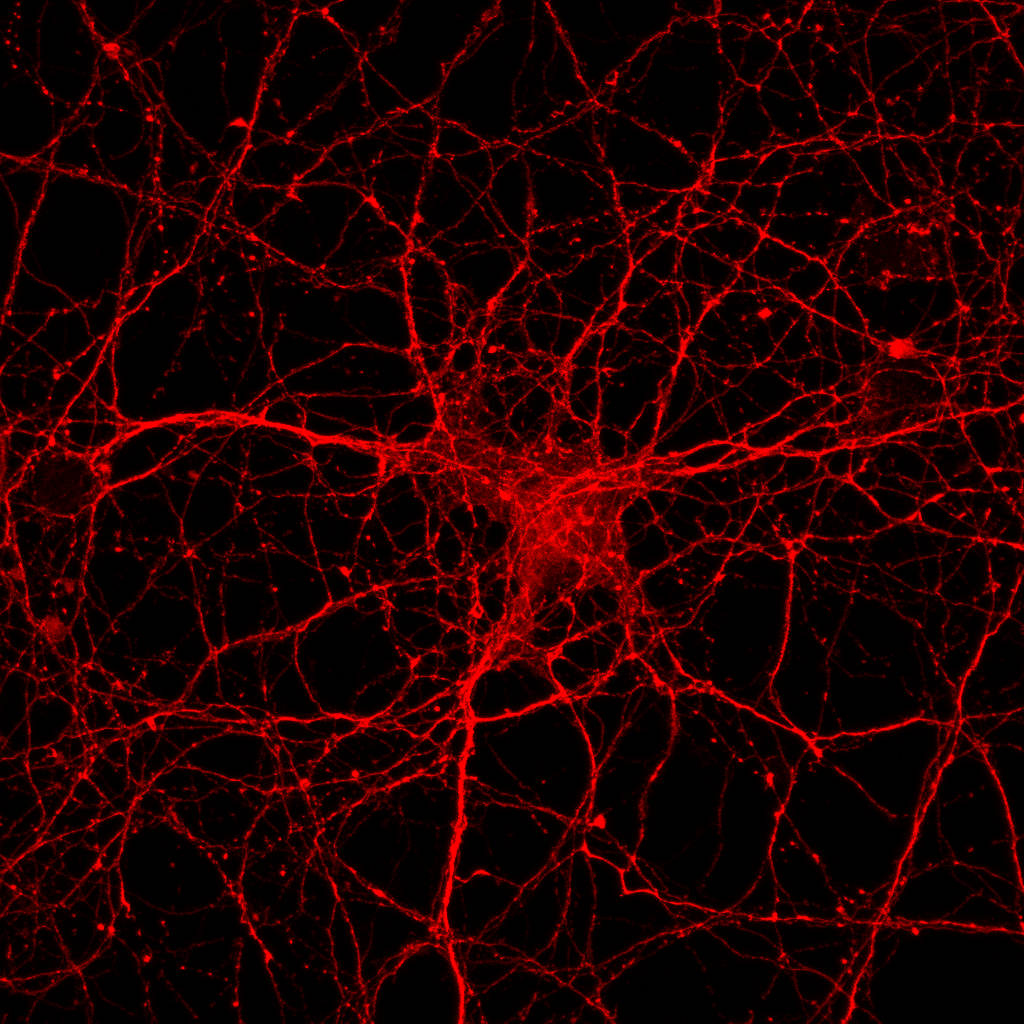

Supplement: Supplementary file 2 — Source data Fig. 1 [file 44319_2026_766_MOESM2_ESM.zip › 1D/G3BP1_MAP2_Tau_Fig1002_4. S.A003_Processed001_ch03.tif]

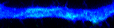

Supplement: Supplementary file 2 — Source data Fig. 1 [file 44319_2026_766_MOESM2_ESM.zip › 1E/1_0000_G3BP1_MAP2_Tau_Fig1002_1. Basal001_Processed001.tif.tif]

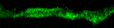

Supplement: Supplementary file 2 — Source data Fig. 1 [file 44319_2026_766_MOESM2_ESM.zip › 1E/1_0001_G3BP1_MAP2_Tau_Fig1002_1. Basal001_Processed001_ch01.tif.tif]

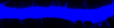

Supplement: Supplementary file 2 — Source data Fig. 1 [file 44319_2026_766_MOESM2_ESM.zip › 1E/1_0002_G3BP1_MAP2_Tau_Fig1002_1. Basal001_Processed001_ch02.tif.tif]

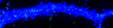

Supplement: Supplementary file 2 — Source data Fig. 1 [file 44319_2026_766_MOESM2_ESM.zip › 1E/2_0000_G3BP1_MAP2_Tau_Fig1002_3. L-glu003_Processed001.tif.tif]

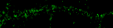

Supplement: Supplementary file 2 — Source data Fig. 1 [file 44319_2026_766_MOESM2_ESM.zip › 1E/2_0002_G3BP1_MAP2_Tau_Fig1002_3. L-glu003_Processed001_ch01.tif.tif]

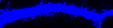

Supplement: Supplementary file 2 — Source data Fig. 1 [file 44319_2026_766_MOESM2_ESM.zip › 1E/2_0003_G3BP1_MAP2_Tau_Fig1002_3. L-glu003_Processed001_ch02.tif.tif]

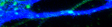

Supplement: Supplementary file 2 — Source data Fig. 1 [file 44319_2026_766_MOESM2_ESM.zip › 1E/4_0000_G3BP1_MAP2_Tau_Fig1002_4. S.A003_Processed001.tif.tif]

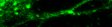

Supplement: Supplementary file 2 — Source data Fig. 1 [file 44319_2026_766_MOESM2_ESM.zip › 1E/4_0001_G3BP1_MAP2_Tau_Fig1002_4. S.A003_Processed001_ch01.tif.tif]

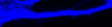

Supplement: Supplementary file 2 — Source data Fig. 1 [file 44319_2026_766_MOESM2_ESM.zip › 1E/4_0002_G3BP1_MAP2_Tau_Fig1002_4. S.A003_Processed001_ch02.tif.tif]

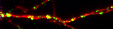

Supplement: Supplementary file 2 — Source data Fig. 1 [file 44319_2026_766_MOESM2_ESM.zip › 1G/1_0000_G3BP1_MAP2_Tau_Fig1002_1. Basal001_Processed001.tif.tif]

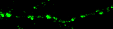

Supplement: Supplementary file 2 — Source data Fig. 1 [file 44319_2026_766_MOESM2_ESM.zip › 1G/1_0001_G3BP1_MAP2_Tau_Fig1002_1. Basal001_Processed001_ch01.tif.tif]

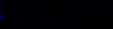

Supplement: Supplementary file 2 — Source data Fig. 1 [file 44319_2026_766_MOESM2_ESM.zip › 1G/1_0002_G3BP1_MAP2_Tau_Fig1002_1. Basal001_Processed001_ch02.tif.tif]

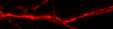

Supplement: Supplementary file 2 — Source data Fig. 1 [file 44319_2026_766_MOESM2_ESM.zip › 1G/1_0003_G3BP1_MAP2_Tau_Fig1002_1. Basal001_Processed001_ch03.tif.tif]

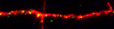

Supplement: Supplementary file 2 — Source data Fig. 1 [file 44319_2026_766_MOESM2_ESM.zip › 1G/3-2_0000_G3BP1_MAP2_Tau_Fig1002_3. L-glu003_Processed001.tif.tif]

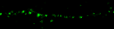

Supplement: Supplementary file 2 — Source data Fig. 1 [file 44319_2026_766_MOESM2_ESM.zip › 1G/3-2_0001_G3BP1_MAP2_Tau_Fig1002_3. L-glu003_Processed001_ch01.tif.tif]

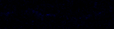

Supplement: Supplementary file 2 — Source data Fig. 1 [file 44319_2026_766_MOESM2_ESM.zip › 1G/3-2_0002_G3BP1_MAP2_Tau_Fig1002_3. L-glu003_Processed001_ch02.tif.tif]

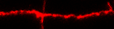

Supplement: Supplementary file 2 — Source data Fig. 1 [file 44319_2026_766_MOESM2_ESM.zip › 1G/3-2_0003_G3BP1_MAP2_Tau_Fig1002_3. L-glu003_Processed001_ch03.tif.tif]

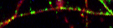

Supplement: Supplementary file 2 — Source data Fig. 1 [file 44319_2026_766_MOESM2_ESM.zip › 1G/4_1_0000_G3BP1_MAP2_Tau_Fig1002_4. S.A003_Processed001.tif.tif]

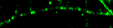

Supplement: Supplementary file 2 — Source data Fig. 1 [file 44319_2026_766_MOESM2_ESM.zip › 1G/4_1_0001_G3BP1_MAP2_Tau_Fig1002_4. S.A003_Processed001_ch01.tif.tif]

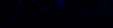

Supplement: Supplementary file 2 — Source data Fig. 1 [file 44319_2026_766_MOESM2_ESM.zip › 1G/4_1_0002_G3BP1_MAP2_Tau_Fig1002_4. S.A003_Processed001_ch02.tif.tif]

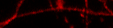

Supplement: Supplementary file 2 — Source data Fig. 1 [file 44319_2026_766_MOESM2_ESM.zip › 1G/4_1_0003_G3BP1_MAP2_Tau_Fig1002_4. S.A003_Processed001_ch03.tif.tif]

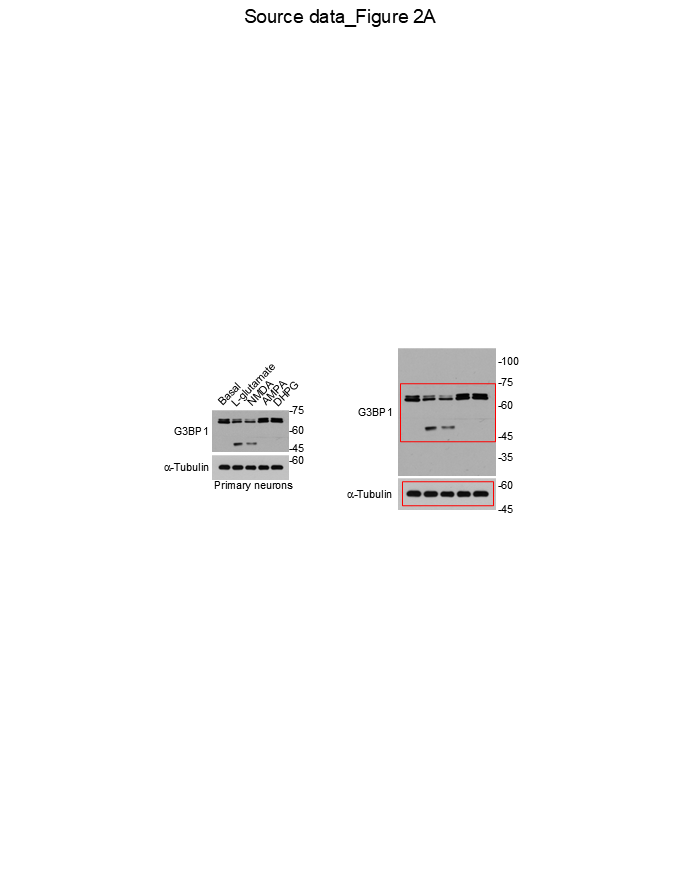

Supplement: Supplementary file 3 — Source data Fig. 2 [file 44319_2026_766_MOESM3_ESM.zip › 2A/Figure2A_Blots.TIF]

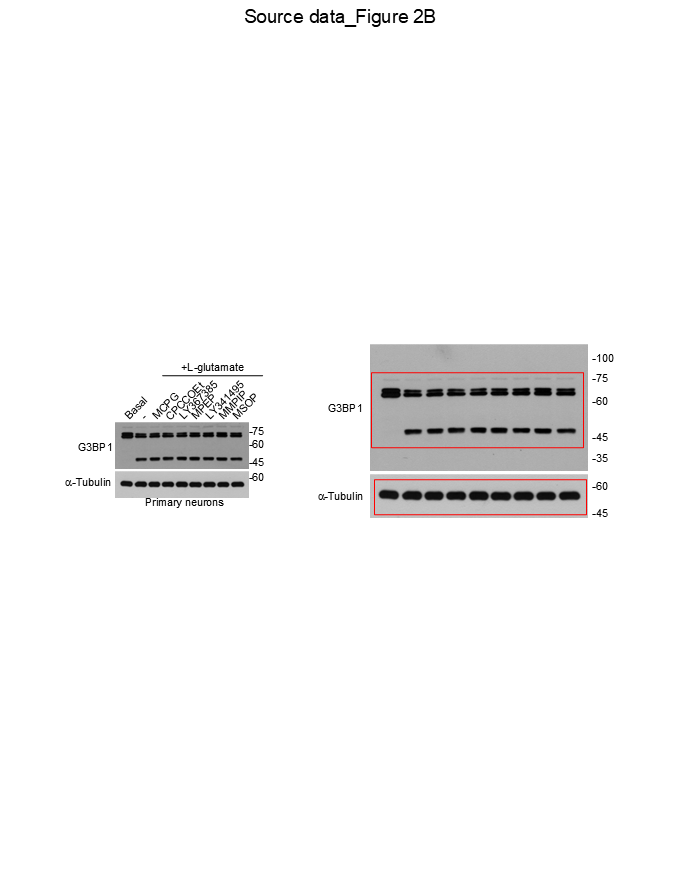

Supplement: Supplementary file 3 — Source data Fig. 2 [file 44319_2026_766_MOESM3_ESM.zip › 2B/Figure2B_Blots.TIF]

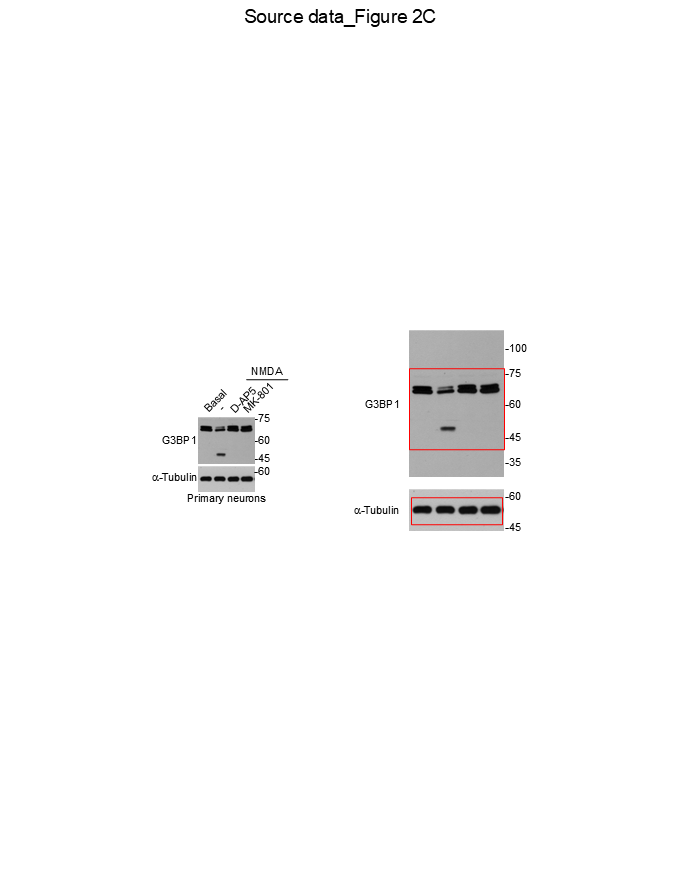

Supplement: Supplementary file 3 — Source data Fig. 2 [file 44319_2026_766_MOESM3_ESM.zip › 2C/Figure2C_Blots.TIF]

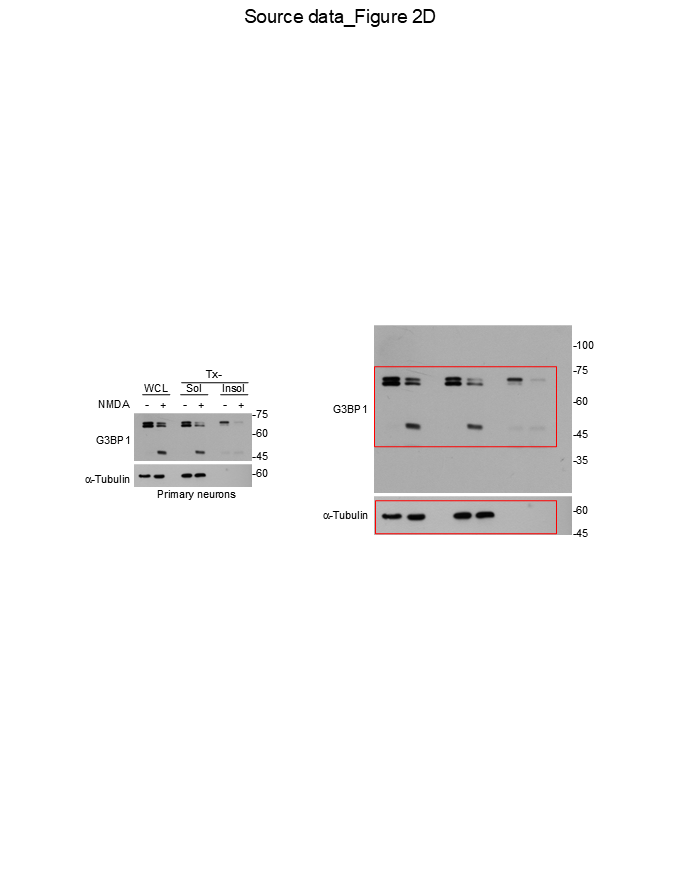

Supplement: Supplementary file 3 — Source data Fig. 2 [file 44319_2026_766_MOESM3_ESM.zip › 2D/Figure2D_Blots.TIF]

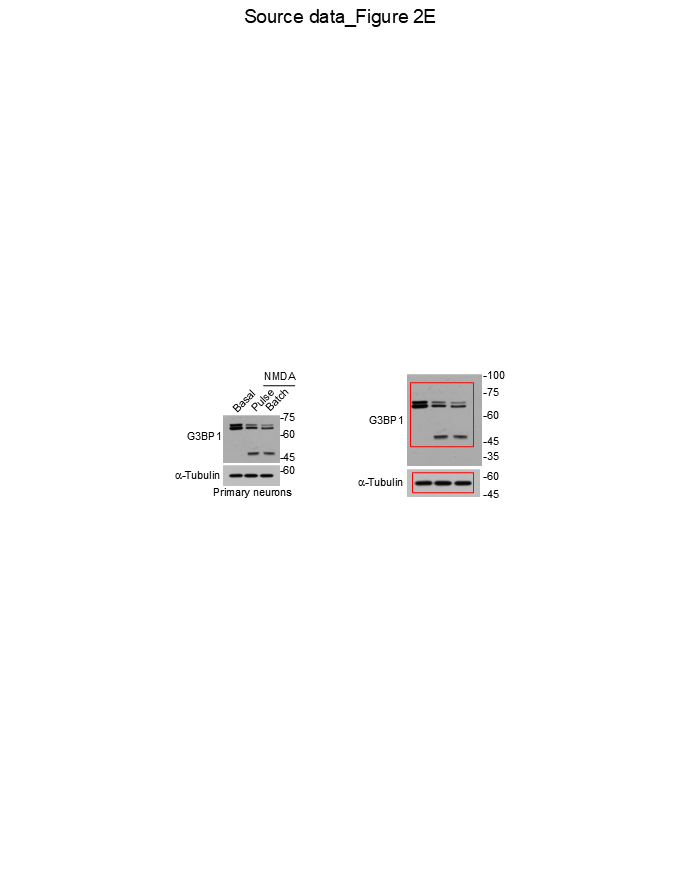

Supplement: Supplementary file 3 — Source data Fig. 2 [file 44319_2026_766_MOESM3_ESM.zip › 2E/Figure2E_Blots.TIF]

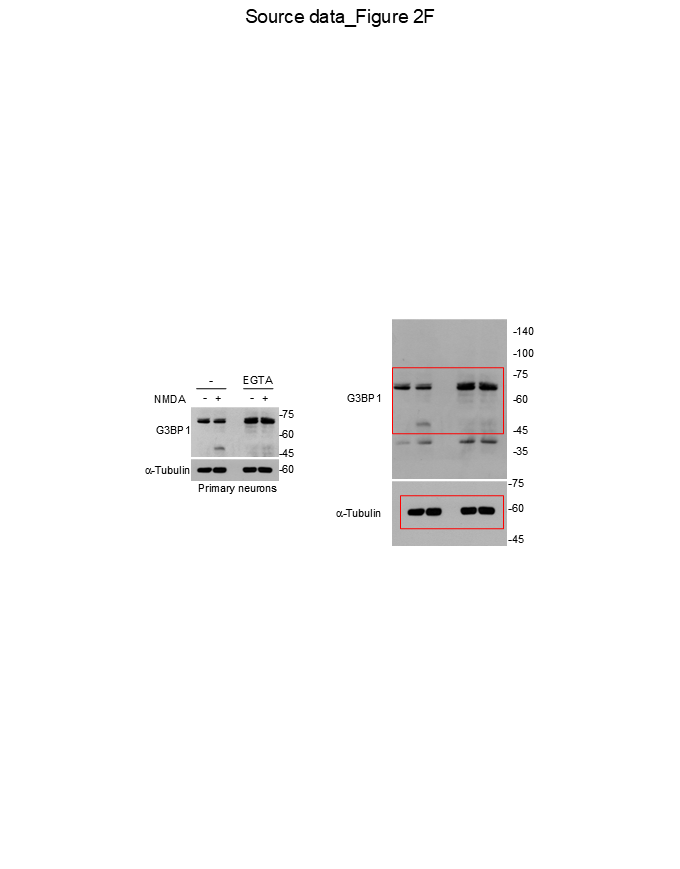

Supplement: Supplementary file 3 — Source data Fig. 2 [file 44319_2026_766_MOESM3_ESM.zip › 2F/Figure2F_Blots.TIF]

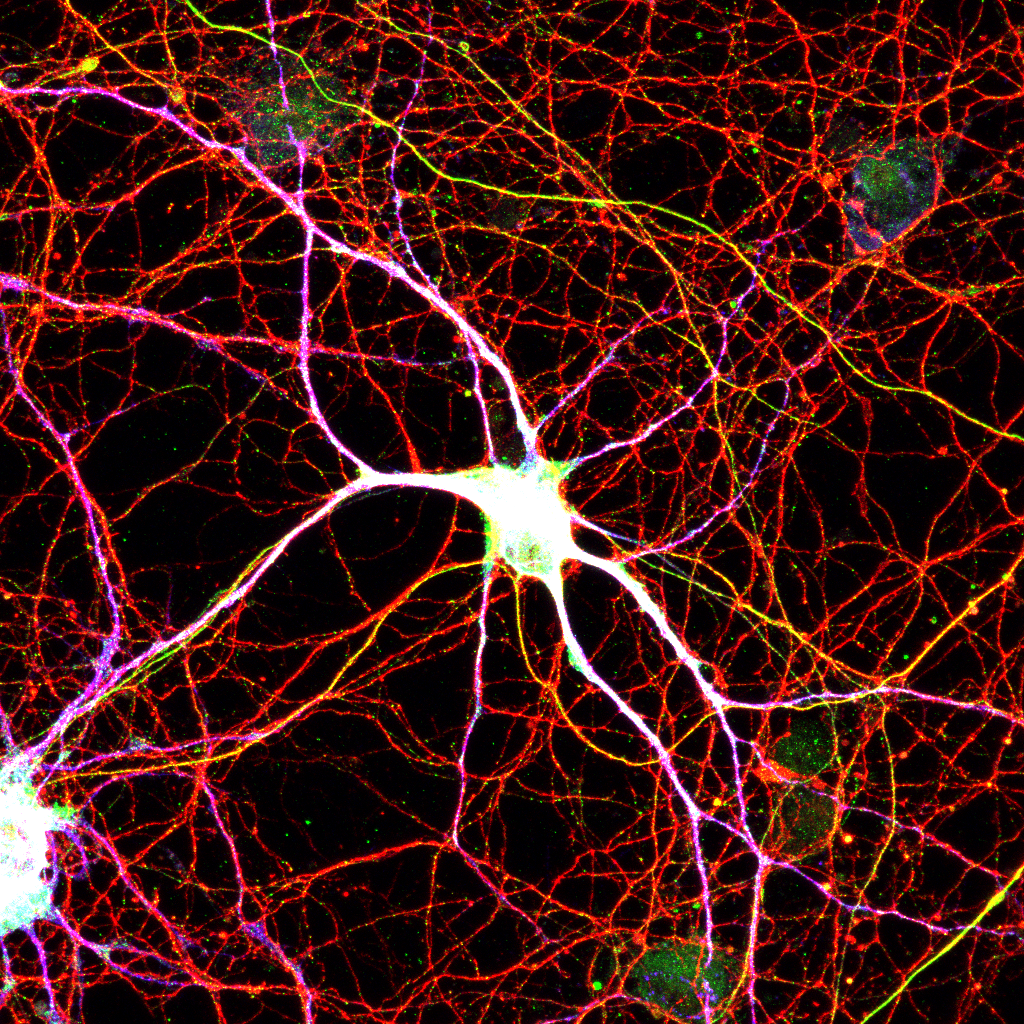

Supplement: Supplementary file 3 — Source data Fig. 2 [file 44319_2026_766_MOESM3_ESM.zip › 2G/Project001_1. Basal002_Processed001green.tif]

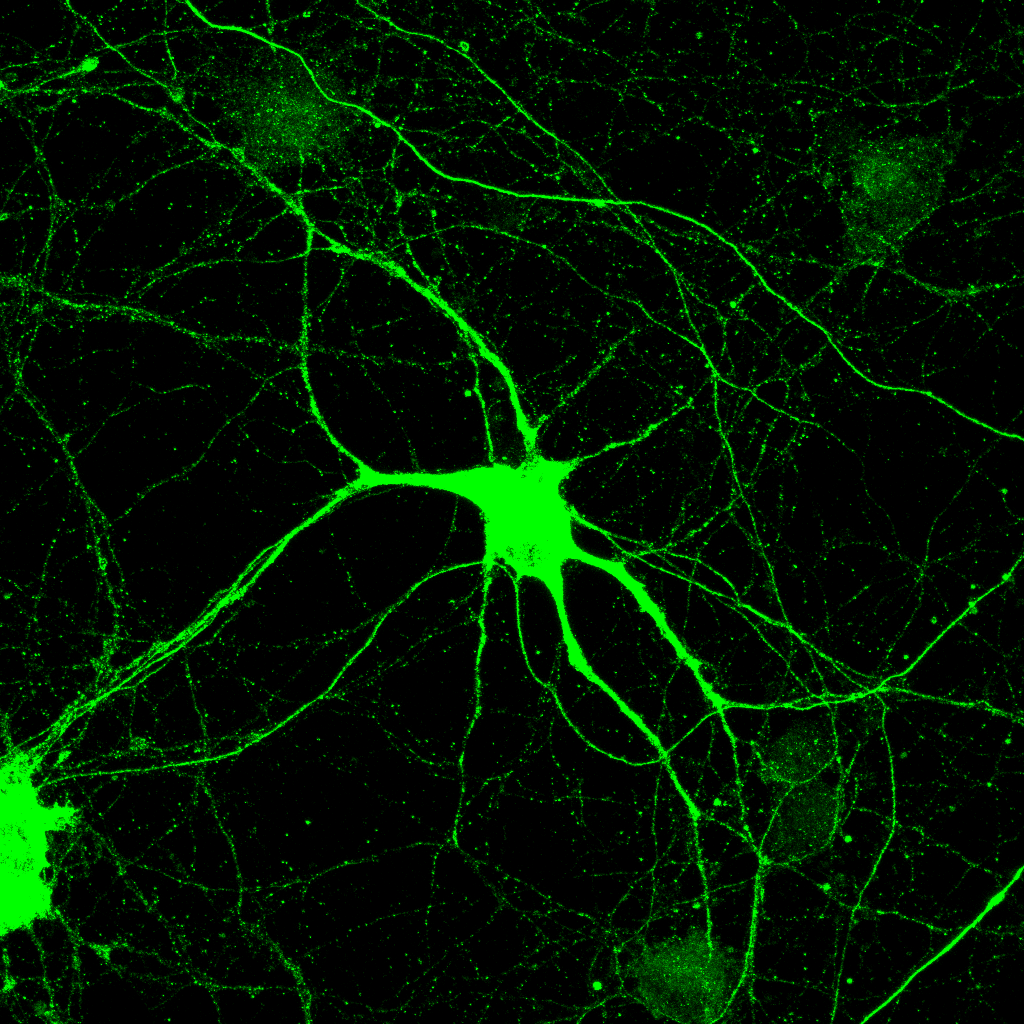

Supplement: Supplementary file 3 — Source data Fig. 2 [file 44319_2026_766_MOESM3_ESM.zip › 2G/Project001_1. Basal002_Processed001_ch01.tif]

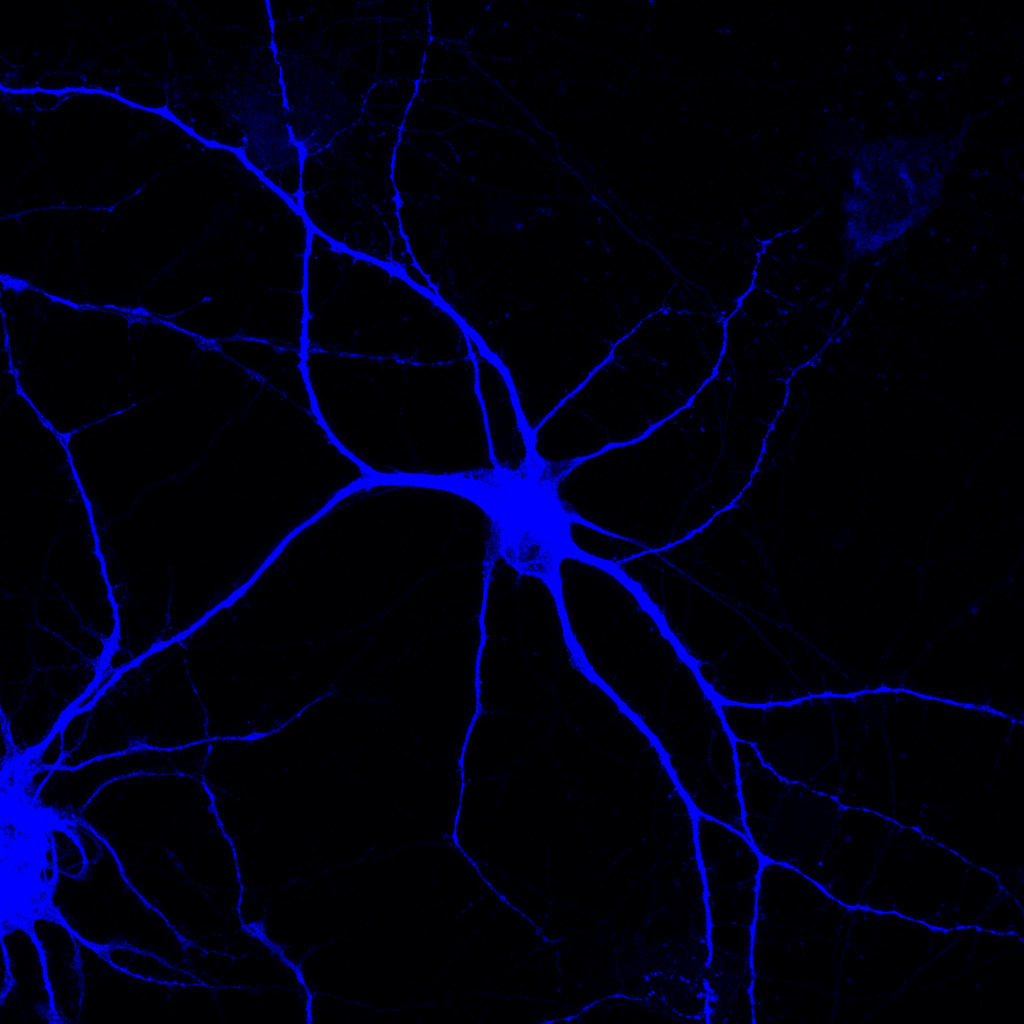

Supplement: Supplementary file 3 — Source data Fig. 2 [file 44319_2026_766_MOESM3_ESM.zip › 2G/Project001_1. Basal002_Processed001_ch02.tif]

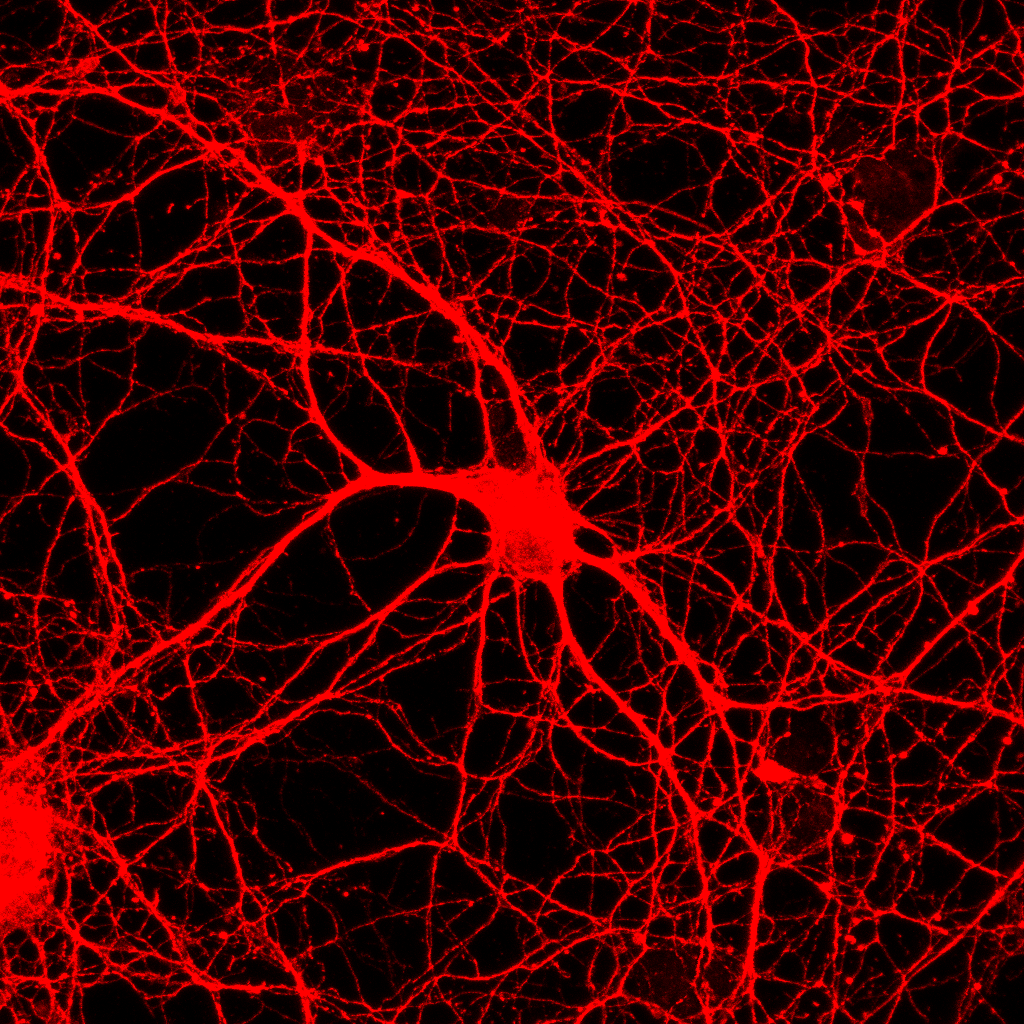

Supplement: Supplementary file 3 — Source data Fig. 2 [file 44319_2026_766_MOESM3_ESM.zip › 2G/Project001_1. Basal002_Processed001_ch03.tif]

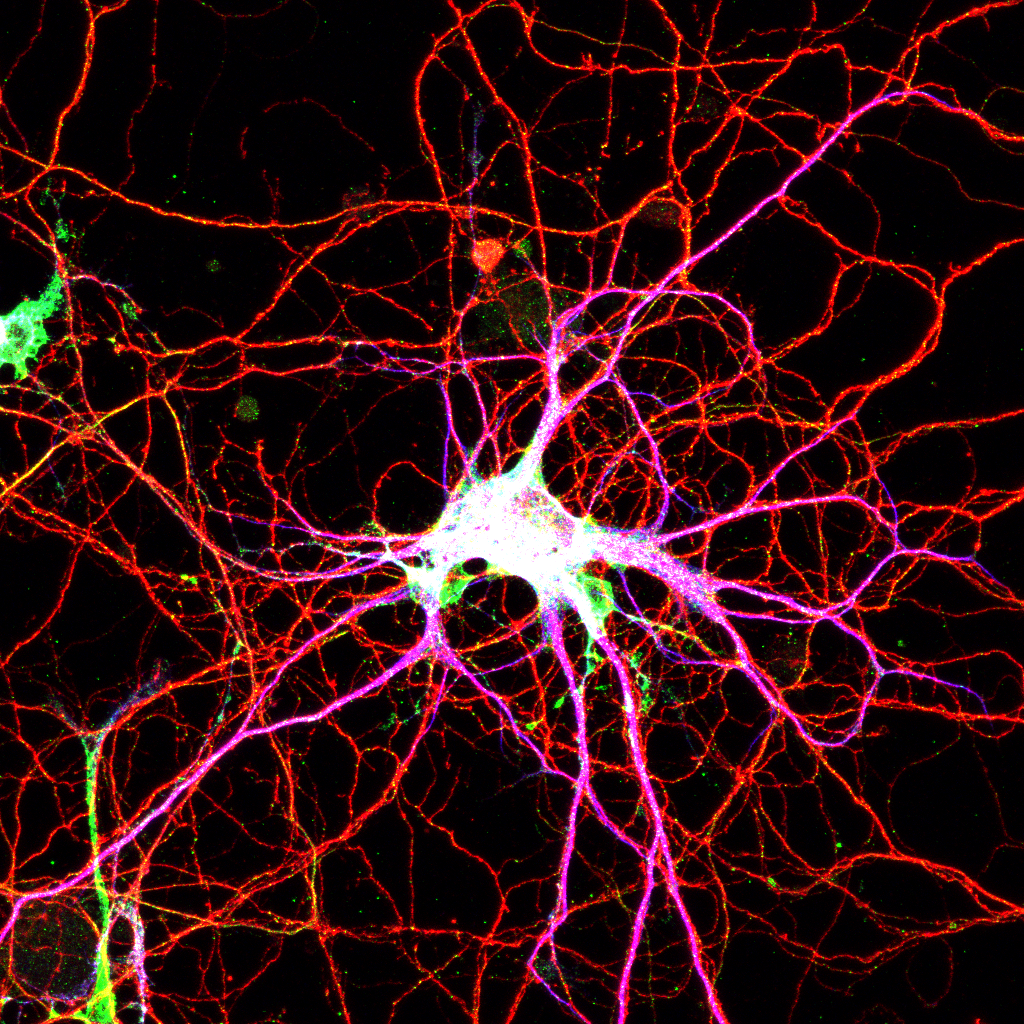

Supplement: Supplementary file 3 — Source data Fig. 2 [file 44319_2026_766_MOESM3_ESM.zip › 2G/Project001_2. NMDA002_Processed001green.tif]

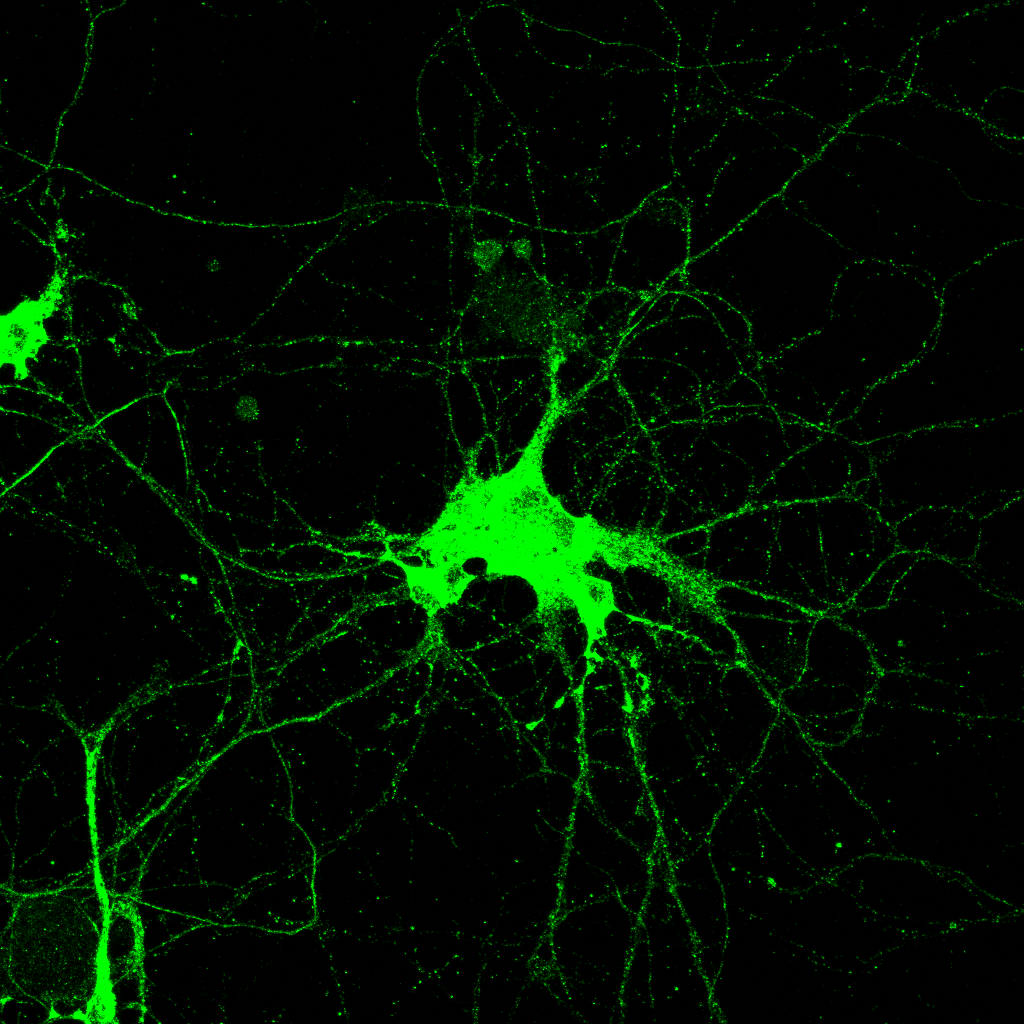

Supplement: Supplementary file 3 — Source data Fig. 2 [file 44319_2026_766_MOESM3_ESM.zip › 2G/Project001_2. NMDA002_Processed001_ch01.tif]

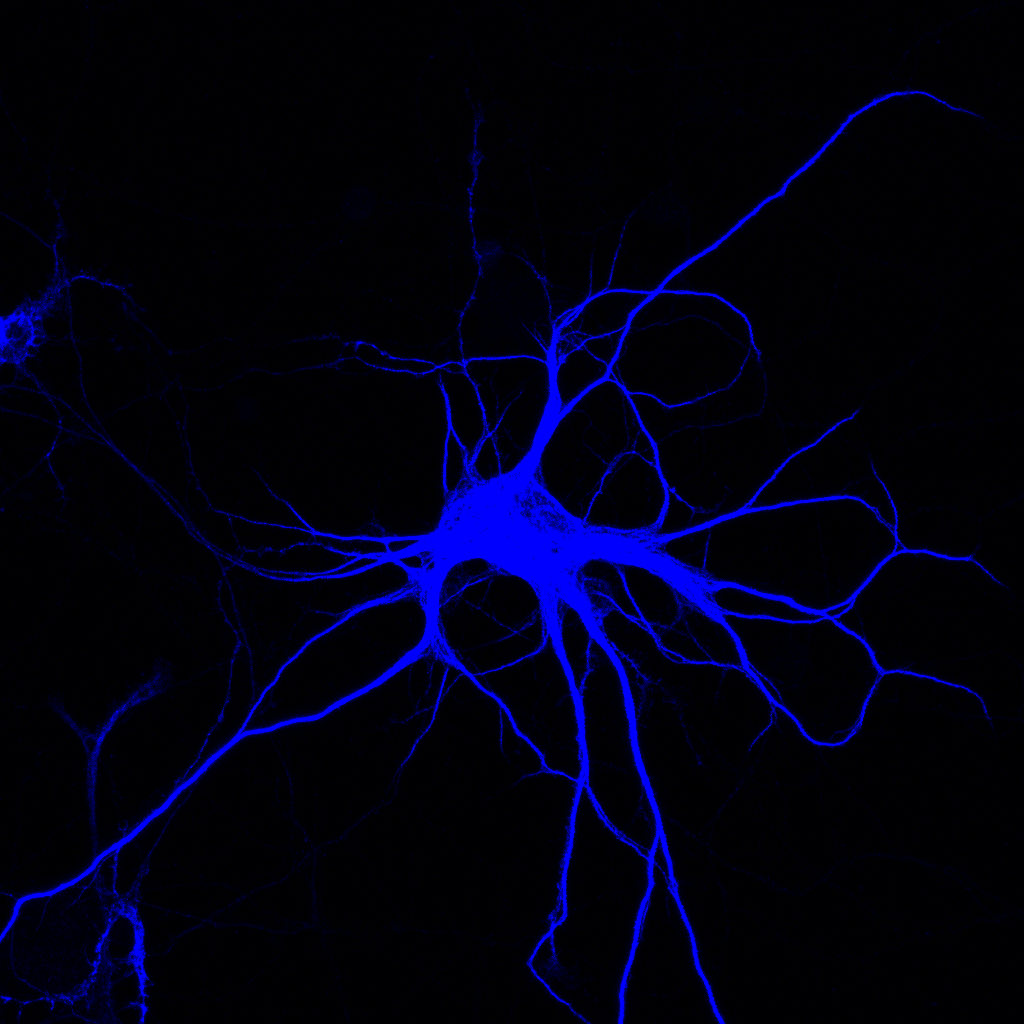

Supplement: Supplementary file 3 — Source data Fig. 2 [file 44319_2026_766_MOESM3_ESM.zip › 2G/Project001_2. NMDA002_Processed001_ch02.tif]

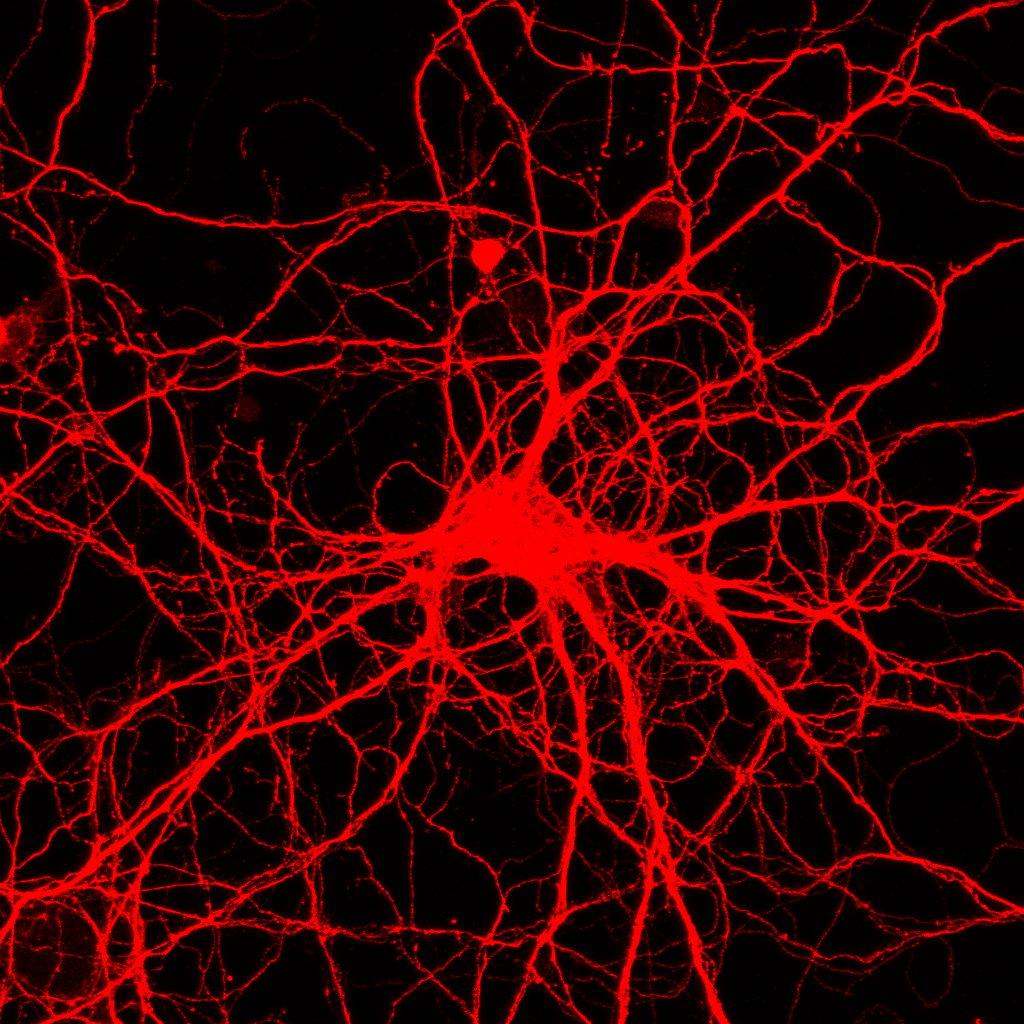

Supplement: Supplementary file 3 — Source data Fig. 2 [file 44319_2026_766_MOESM3_ESM.zip › 2G/Project001_2. NMDA002_Processed001_ch03.tif]

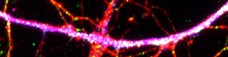

Supplement: Supplementary file 3 — Source data Fig. 2 [file 44319_2026_766_MOESM3_ESM.zip › 2H/1. Den_0000_Project001_1. Basal002_Processed001green.tif.tif]

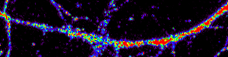

Supplement: Supplementary file 3 — Source data Fig. 2 [file 44319_2026_766_MOESM3_ESM.zip › 2H/1. Den_0001_Project001_1. Basal002_Processed001.tif.tif]

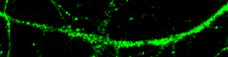

Supplement: Supplementary file 3 — Source data Fig. 2 [file 44319_2026_766_MOESM3_ESM.zip › 2H/1. Den_0002_Project001_1. Basal002_Processed001_ch01.tif.tif]

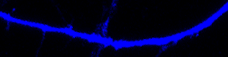

Supplement: Supplementary file 3 — Source data Fig. 2 [file 44319_2026_766_MOESM3_ESM.zip › 2H/1. Den_0003_Project001_1. Basal002_Processed001_ch02.tif.tif]

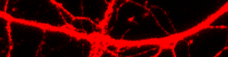

Supplement: Supplementary file 3 — Source data Fig. 2 [file 44319_2026_766_MOESM3_ESM.zip › 2H/1. Den_0004_Project001_1. Basal002_Processed001_ch03.tif.tif]

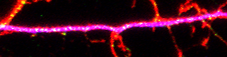

Supplement: Supplementary file 3 — Source data Fig. 2 [file 44319_2026_766_MOESM3_ESM.zip › 2H/2. Den_0000_Project001_2. NMDA002_Processed001green.tif.tif]

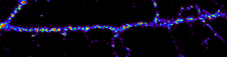

Supplement: Supplementary file 3 — Source data Fig. 2 [file 44319_2026_766_MOESM3_ESM.zip › 2H/2. Den_0001_Project001_2. NMDA002_Processed001.tif.tif]

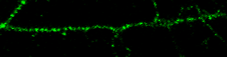

Supplement: Supplementary file 3 — Source data Fig. 2 [file 44319_2026_766_MOESM3_ESM.zip › 2H/2. Den_0002_Project001_2. NMDA002_Processed001_ch01.tif.tif]

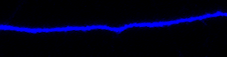

Supplement: Supplementary file 3 — Source data Fig. 2 [file 44319_2026_766_MOESM3_ESM.zip › 2H/2. Den_0003_Project001_2. NMDA002_Processed001_ch02.tif.tif]

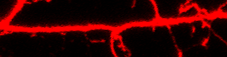

Supplement: Supplementary file 3 — Source data Fig. 2 [file 44319_2026_766_MOESM3_ESM.zip › 2H/2. Den_0004_Project001_2. NMDA002_Processed001_ch03.tif.tif]

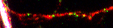

Supplement: Supplementary file 3 — Source data Fig. 2 [file 44319_2026_766_MOESM3_ESM.zip › 2I/1. Axon_0000_Project001_1. Basal002_Processed001green.tif.tif]

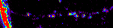

Supplement: Supplementary file 3 — Source data Fig. 2 [file 44319_2026_766_MOESM3_ESM.zip › 2I/1. Axon_0001_Project001_1. Basal002_Processed001.tif.tif]

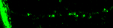

Supplement: Supplementary file 3 — Source data Fig. 2 [file 44319_2026_766_MOESM3_ESM.zip › 2I/1. Axon_0002_Project001_1. Basal002_Processed001_ch01.tif.tif]

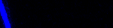

Supplement: Supplementary file 3 — Source data Fig. 2 [file 44319_2026_766_MOESM3_ESM.zip › 2I/1. Axon_0003_Project001_1. Basal002_Processed001_ch02.tif.tif]

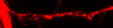

Supplement: Supplementary file 3 — Source data Fig. 2 [file 44319_2026_766_MOESM3_ESM.zip › 2I/1. Axon_0004_Project001_1. Basal002_Processed001_ch03.tif.tif]

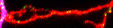

Supplement: Supplementary file 3 — Source data Fig. 2 [file 44319_2026_766_MOESM3_ESM.zip › 2I/2. axon_0000_Project001_2. NMDA002_Processed001green.tif.tif]

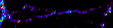

Supplement: Supplementary file 3 — Source data Fig. 2 [file 44319_2026_766_MOESM3_ESM.zip › 2I/2. axon_0001_Project001_2. NMDA002_Processed001.tif.tif]

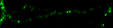

Supplement: Supplementary file 3 — Source data Fig. 2 [file 44319_2026_766_MOESM3_ESM.zip › 2I/2. axon_0002_Project001_2. NMDA002_Processed001_ch01.tif.tif]

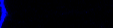

Supplement: Supplementary file 3 — Source data Fig. 2 [file 44319_2026_766_MOESM3_ESM.zip › 2I/2. axon_0003_Project001_2. NMDA002_Processed001_ch02.tif.tif]

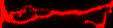

Supplement: Supplementary file 3 — Source data Fig. 2 [file 44319_2026_766_MOESM3_ESM.zip › 2I/2. axon_0004_Project001_2. NMDA002_Processed001_ch03.tif.tif]

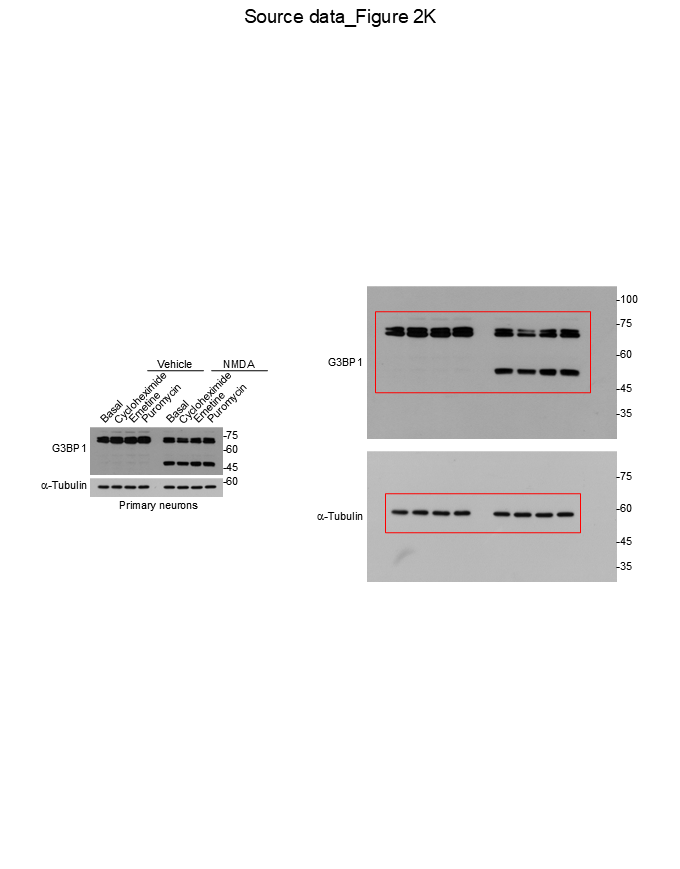

Supplement: Supplementary file 3 — Source data Fig. 2 [file 44319_2026_766_MOESM3_ESM.zip › 2K/Figure2K_Blots.TIF]

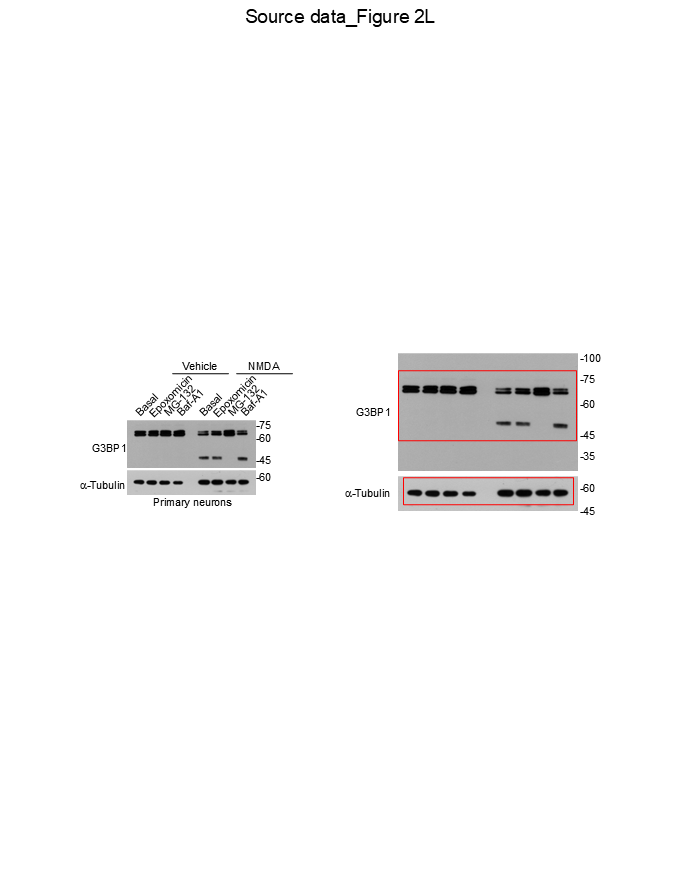

Supplement: Supplementary file 3 — Source data Fig. 2 [file 44319_2026_766_MOESM3_ESM.zip › 2L/Figure2L_Blots.TIF]

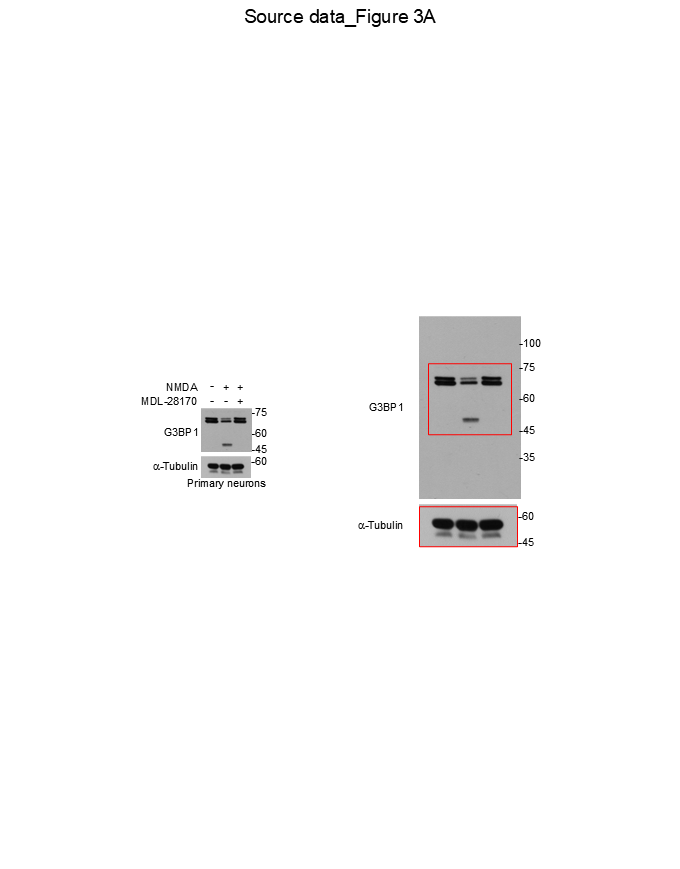

Supplement: Supplementary file 4 — Source data Fig. 3 [file 44319_2026_766_MOESM4_ESM.zip › 3A/Figure3A_Blots.TIF]

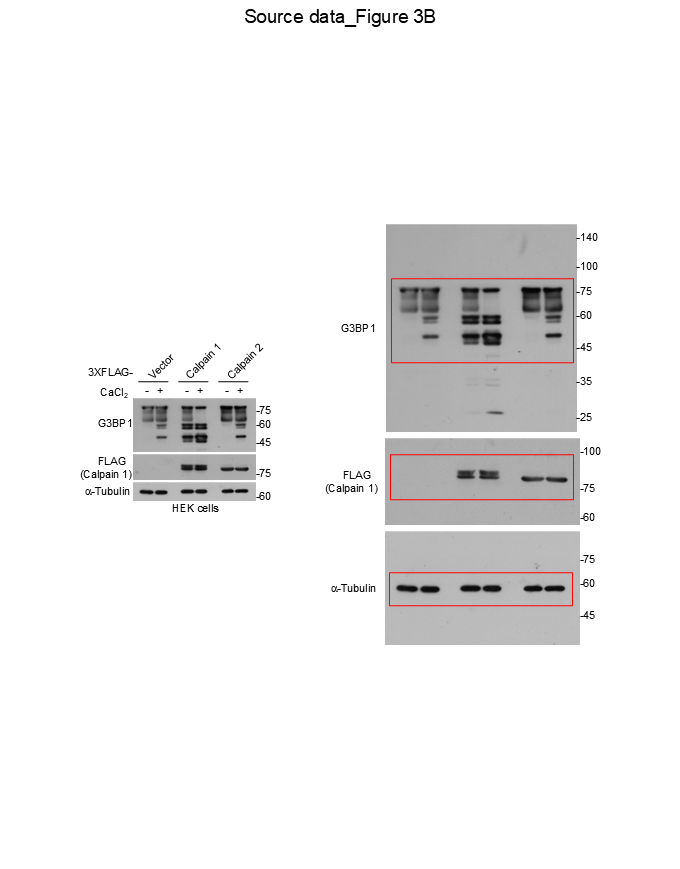

Supplement: Supplementary file 4 — Source data Fig. 3 [file 44319_2026_766_MOESM4_ESM.zip › 3B/Figure3B_Blots.TIF]

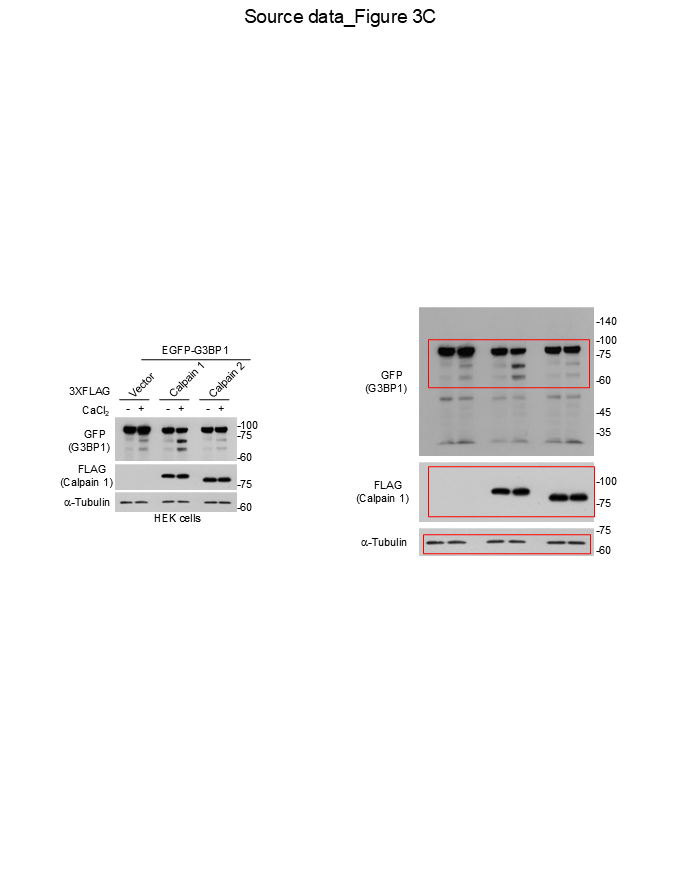

Supplement: Supplementary file 4 — Source data Fig. 3 [file 44319_2026_766_MOESM4_ESM.zip › 3C/Figure3C_Blots.TIF]

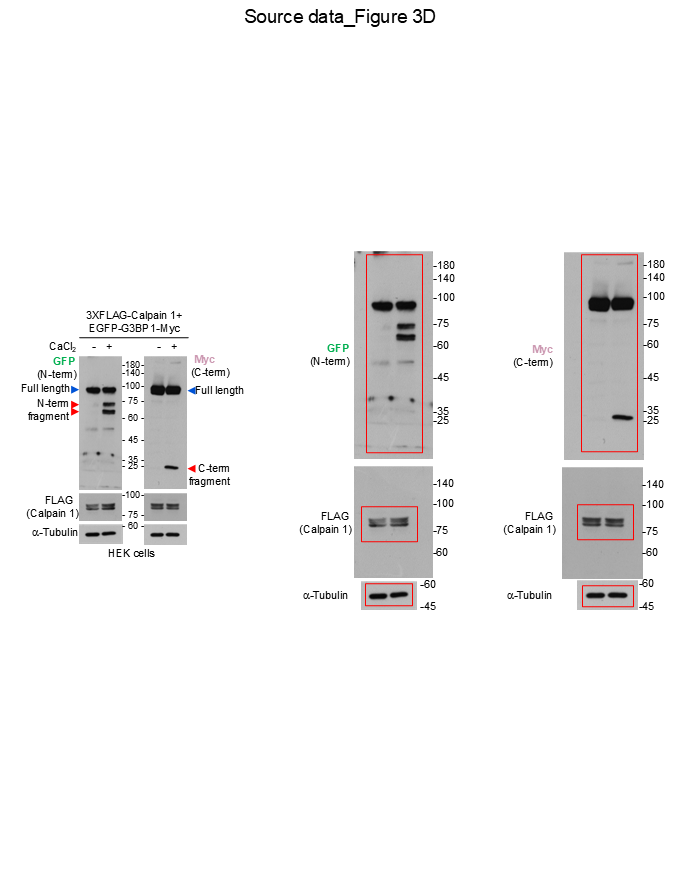

Supplement: Supplementary file 4 — Source data Fig. 3 [file 44319_2026_766_MOESM4_ESM.zip › 3D/Figure3D_Blots.TIF]

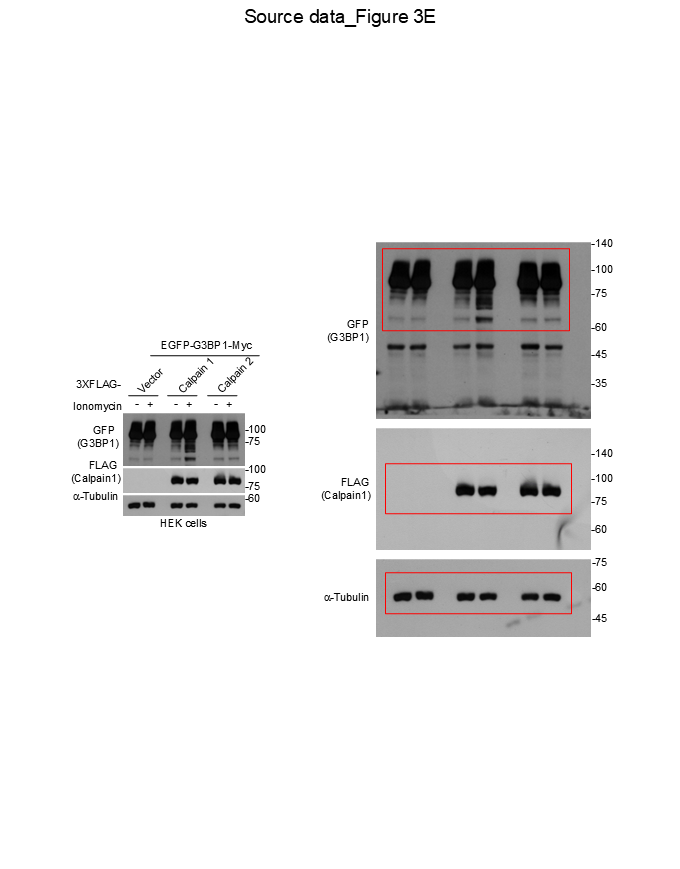

Supplement: Supplementary file 4 — Source data Fig. 3 [file 44319_2026_766_MOESM4_ESM.zip › 3E/Figure3E_Blots.TIF]

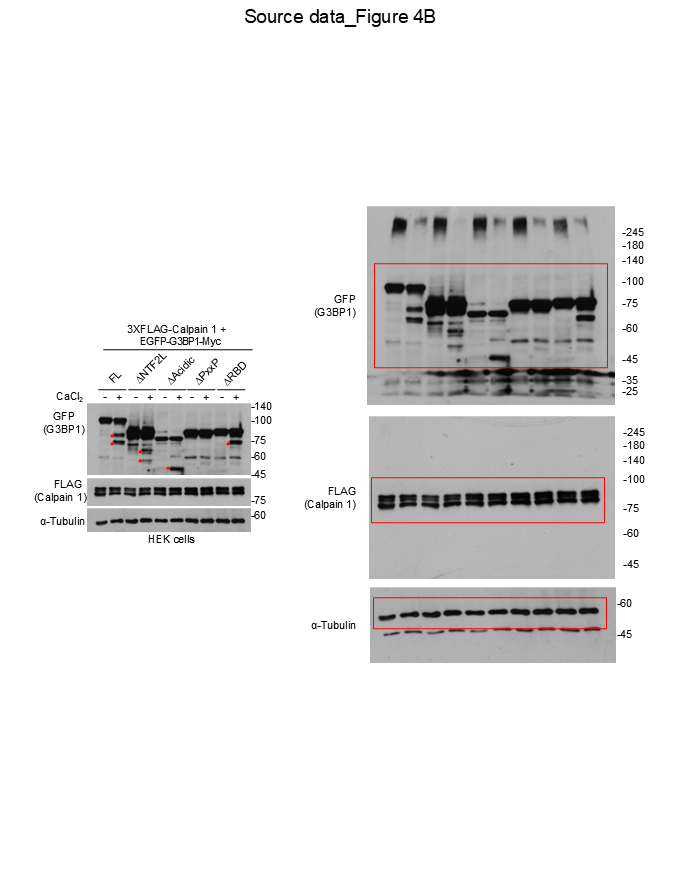

Supplement: Supplementary file 5 — Source data Fig. 4 [file 44319_2026_766_MOESM5_ESM.zip › 4B/Figure4B_Blots.TIF]
