# Supplementary figures and images for: Proteolytic cleavage of G3BP1 by calpain 1 couples NMDAR activation to mTOR-dependent local translation (part 2 of 2)
Source: EMBO Rep. 2026 Apr 4;27(10):2749–71. doi: 10.1038/s44319-026-00766-9 (PMC13219515; doi:10.1038/s44319-026-00766-9)

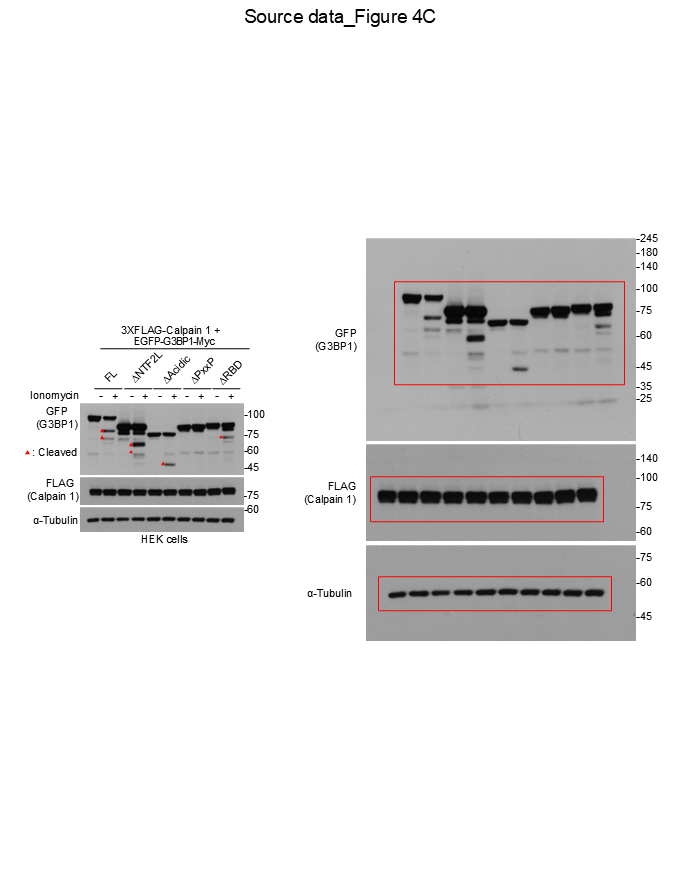

Supplement: Supplementary file 5 — Source data Fig. 4 [file 44319_2026_766_MOESM5_ESM.zip › 4C/Figure4C_Blots.TIF]

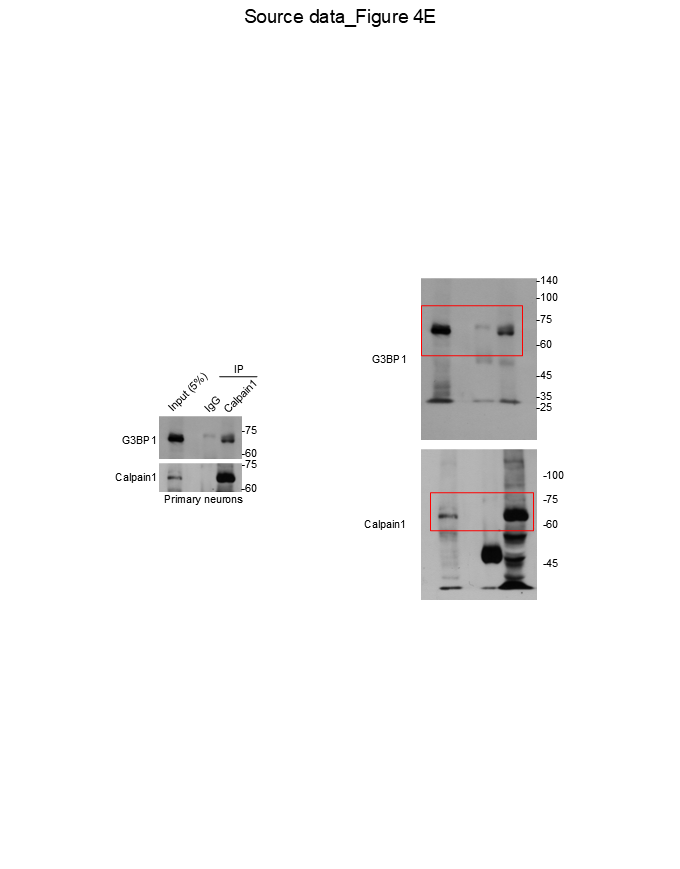

Supplement: Supplementary file 5 — Source data Fig. 4 [file 44319_2026_766_MOESM5_ESM.zip › 4E/Figure4E_Blots.TIF]

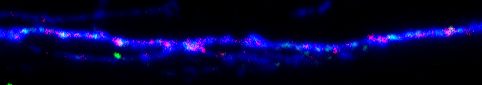

Supplement: Supplementary file 5 — Source data Fig. 4 [file 44319_2026_766_MOESM5_ESM.zip › 4F/1_0000_251128 Cal G3BP coloc002MERGED_1. Cal1012_Processed001.tif.tif]

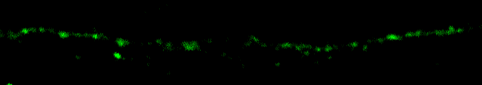

Supplement: Supplementary file 5 — Source data Fig. 4 [file 44319_2026_766_MOESM5_ESM.zip › 4F/1_0001_251128 Cal G3BP coloc002MERGED_1. Cal1012_Processed001_ch00.tif.tif]

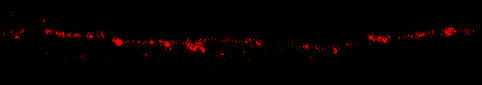

Supplement: Supplementary file 5 — Source data Fig. 4 [file 44319_2026_766_MOESM5_ESM.zip › 4F/1_0002_251128 Cal G3BP coloc002MERGED_1. Cal1012_Processed001_ch01.tif.tif]

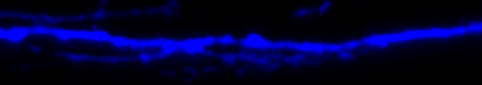

Supplement: Supplementary file 5 — Source data Fig. 4 [file 44319_2026_766_MOESM5_ESM.zip › 4F/1_0003_251128 Cal G3BP coloc002MERGED_1. Cal1012_Processed001_ch02.tif.tif]

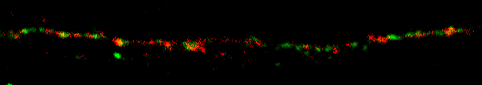

Supplement: Supplementary file 5 — Source data Fig. 4 [file 44319_2026_766_MOESM5_ESM.zip › 4F/1_0004_251128 Cal G3BP coloc002MERGED_1. Cal1012_Processed001GR.tif.tif]

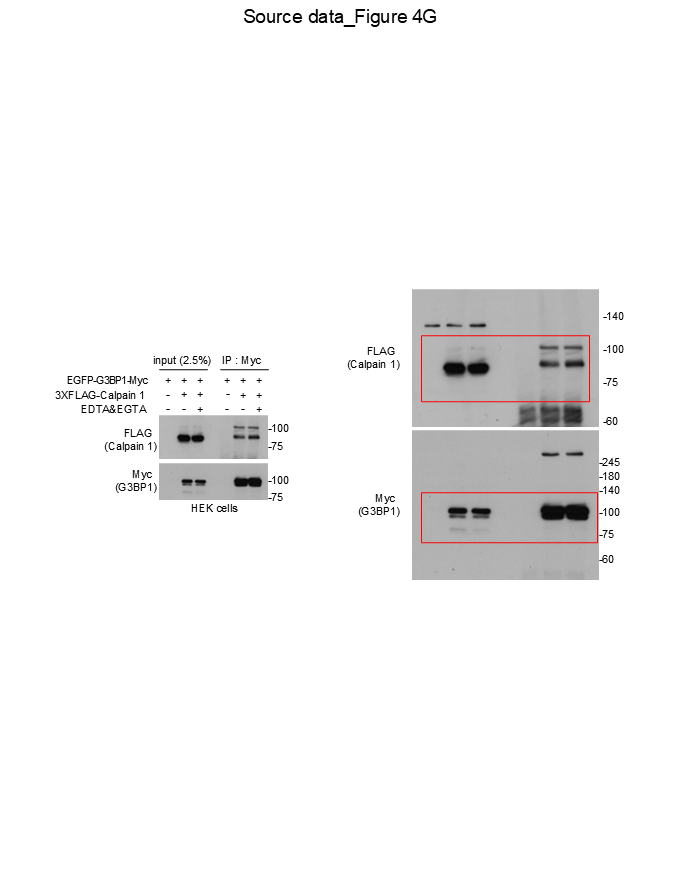

Supplement: Supplementary file 5 — Source data Fig. 4 [file 44319_2026_766_MOESM5_ESM.zip › 4G/Figure4G_Blots.TIF]

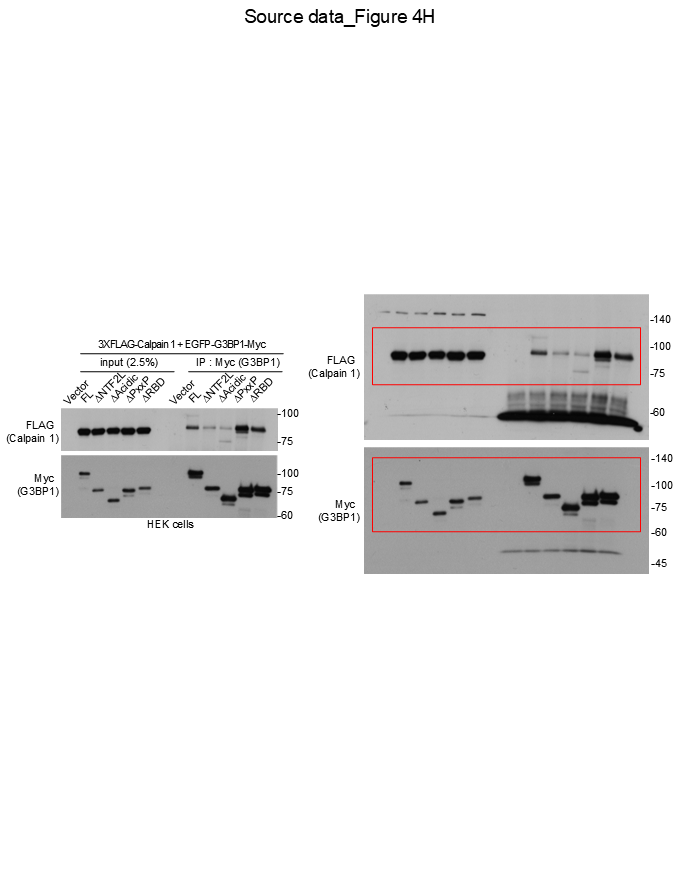

Supplement: Supplementary file 5 — Source data Fig. 4 [file 44319_2026_766_MOESM5_ESM.zip › 4H/Figure4H_Blots.TIF]

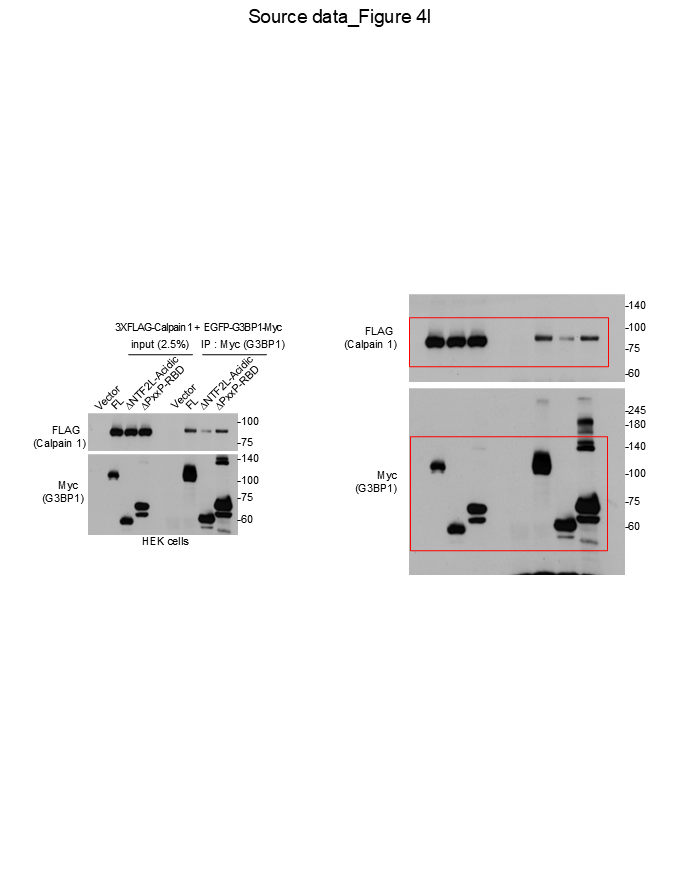

Supplement: Supplementary file 5 — Source data Fig. 4 [file 44319_2026_766_MOESM5_ESM.zip › 4I/Figure4I_Blots.TIF]

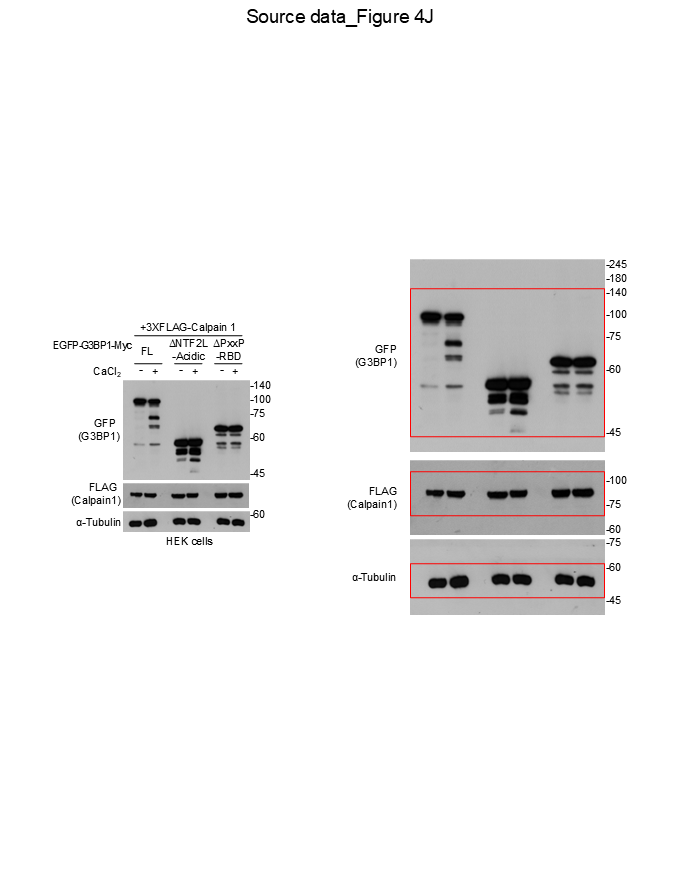

Supplement: Supplementary file 5 — Source data Fig. 4 [file 44319_2026_766_MOESM5_ESM.zip › 4J/Figure4J_Blots.TIF]

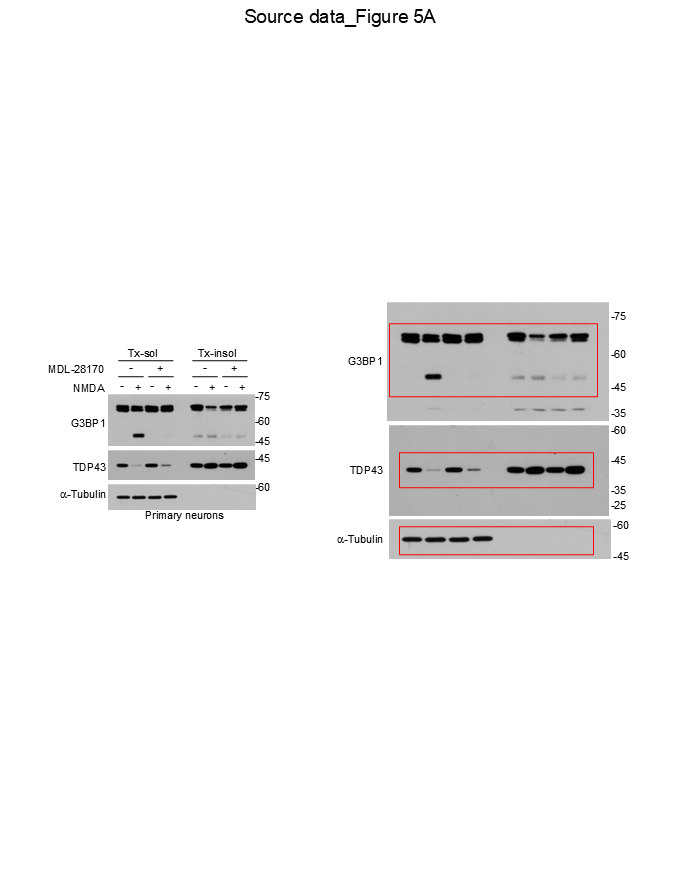

Supplement: Supplementary file 6 — Source data Fig. 5 [file 44319_2026_766_MOESM6_ESM.zip › 5A/Figure5A_Blots.TIF]

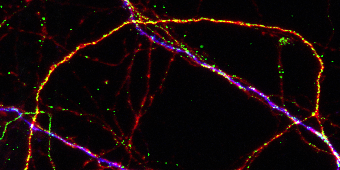

Supplement: Supplementary file 6 — Source data Fig. 5 [file 44319_2026_766_MOESM6_ESM.zip › 5B/1_1_0000_Project001_1. Basal003_Processed001.tif.tif]

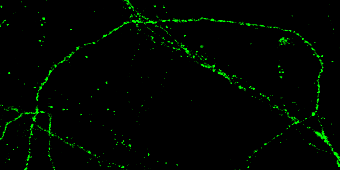

Supplement: Supplementary file 6 — Source data Fig. 5 [file 44319_2026_766_MOESM6_ESM.zip › 5B/1_1_0002_Project001_1. Basal003_Processed001_ch01.tif.tif]

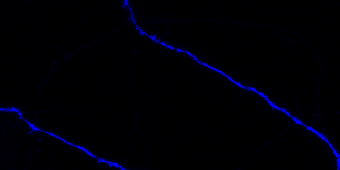

Supplement: Supplementary file 6 — Source data Fig. 5 [file 44319_2026_766_MOESM6_ESM.zip › 5B/1_1_0003_Project001_1. Basal003_Processed001_ch02.tif.tif]

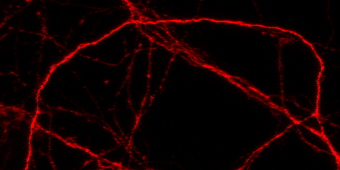

Supplement: Supplementary file 6 — Source data Fig. 5 [file 44319_2026_766_MOESM6_ESM.zip › 5B/1_1_0004_Project001_1. Basal003_Processed001_ch03.tif.tif]

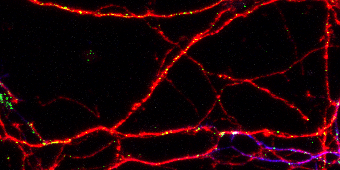

Supplement: Supplementary file 6 — Source data Fig. 5 [file 44319_2026_766_MOESM6_ESM.zip › 5B/2_1_0000_Project001_2. NMDA002_Processed001.tif.tif]

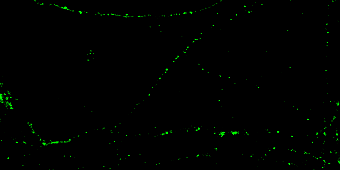

Supplement: Supplementary file 6 — Source data Fig. 5 [file 44319_2026_766_MOESM6_ESM.zip › 5B/2_1_0002_Project001_2. NMDA002_Processed001_ch01.tif.tif]

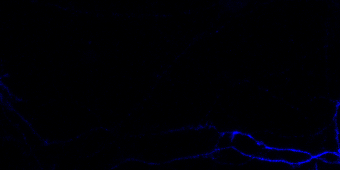

Supplement: Supplementary file 6 — Source data Fig. 5 [file 44319_2026_766_MOESM6_ESM.zip › 5B/2_1_0003_Project001_2. NMDA002_Processed001_ch02.tif.tif]

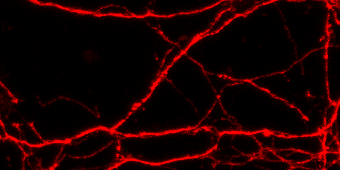

Supplement: Supplementary file 6 — Source data Fig. 5 [file 44319_2026_766_MOESM6_ESM.zip › 5B/2_1_0004_Project001_2. NMDA002_Processed001_ch03.tif.tif]

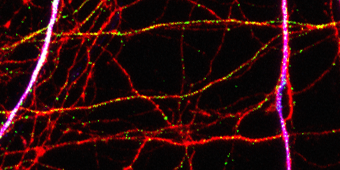

Supplement: Supplementary file 6 — Source data Fig. 5 [file 44319_2026_766_MOESM6_ESM.zip › 5B/3_2_0000_Project001_4. Calin+NMDA001_Processed001.tif.tif]

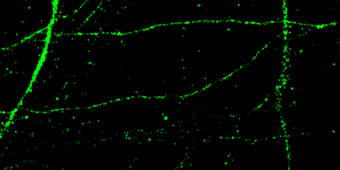

Supplement: Supplementary file 6 — Source data Fig. 5 [file 44319_2026_766_MOESM6_ESM.zip › 5B/3_2_0002_Project001_4. Calin+NMDA001_Processed001_ch01.tif.tif]

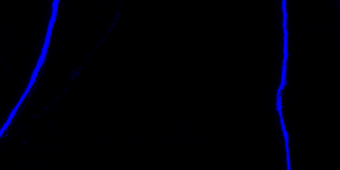

Supplement: Supplementary file 6 — Source data Fig. 5 [file 44319_2026_766_MOESM6_ESM.zip › 5B/3_2_0003_Project001_4. Calin+NMDA001_Processed001_ch02.tif.tif]

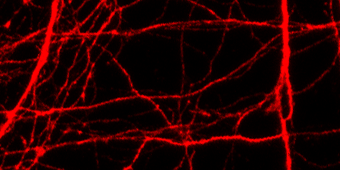

Supplement: Supplementary file 6 — Source data Fig. 5 [file 44319_2026_766_MOESM6_ESM.zip › 5B/3_2_0004_Project001_4. Calin+NMDA001_Processed001_ch03.tif.tif]

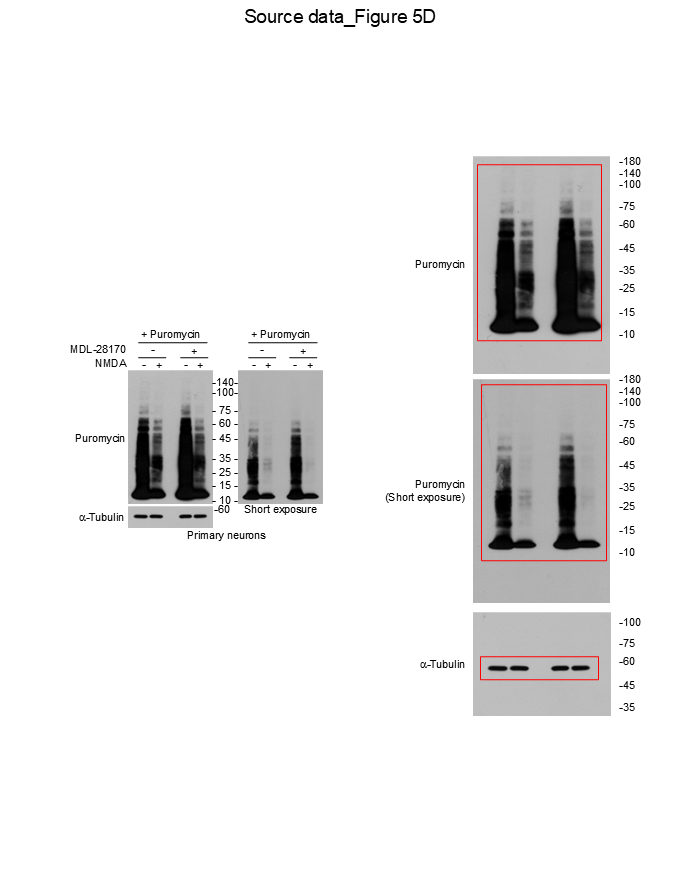

Supplement: Supplementary file 6 — Source data Fig. 5 [file 44319_2026_766_MOESM6_ESM.zip › 5D/Figure5D_Blots.TIF]

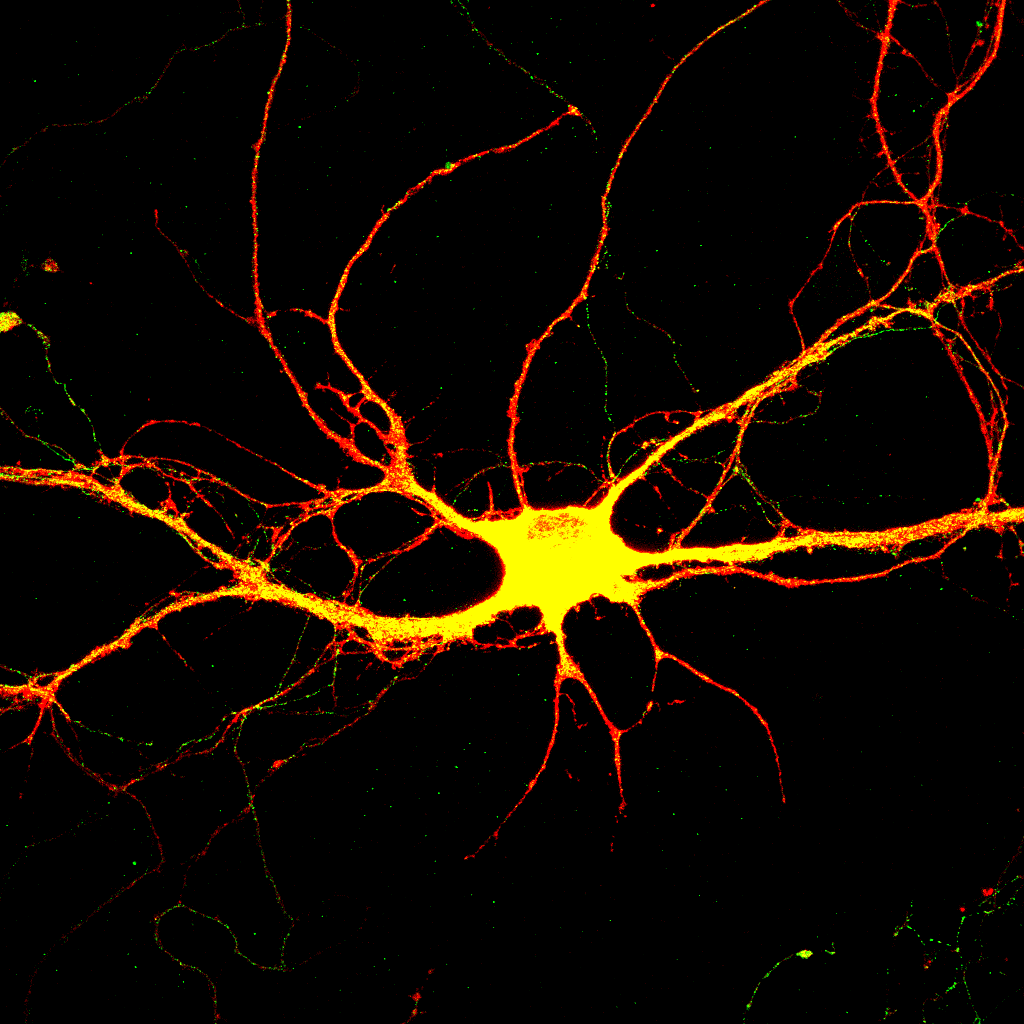

Supplement: Supplementary file 6 — Source data Fig. 5 [file 44319_2026_766_MOESM6_ESM.zip › 5E/122024NMDA RPM -+ Cal in002_1. Basal001_Processed001.tif]

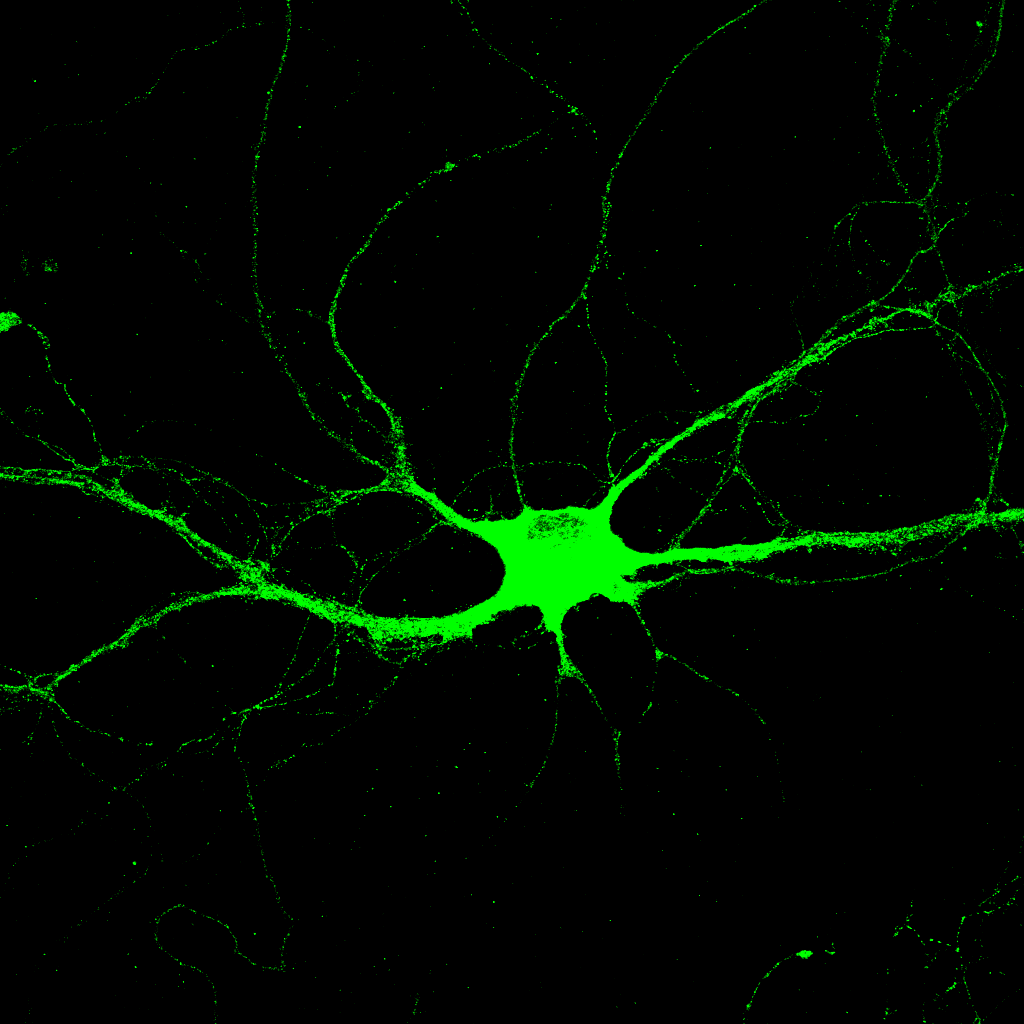

Supplement: Supplementary file 6 — Source data Fig. 5 [file 44319_2026_766_MOESM6_ESM.zip › 5E/122024NMDA RPM -+ Cal in002_1. Basal001_Processed001_ch00.tif]

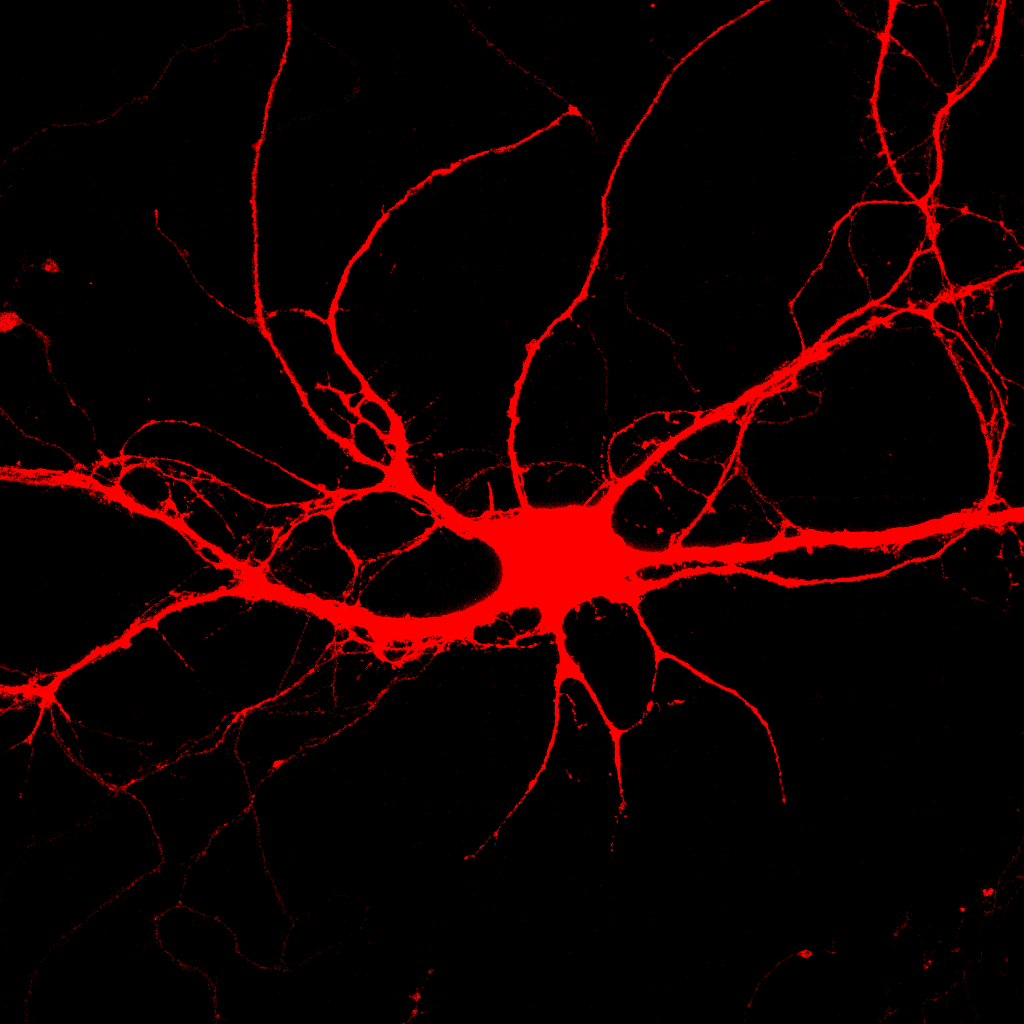

Supplement: Supplementary file 6 — Source data Fig. 5 [file 44319_2026_766_MOESM6_ESM.zip › 5E/122024NMDA RPM -+ Cal in002_1. Basal001_Processed001_ch01.tif]

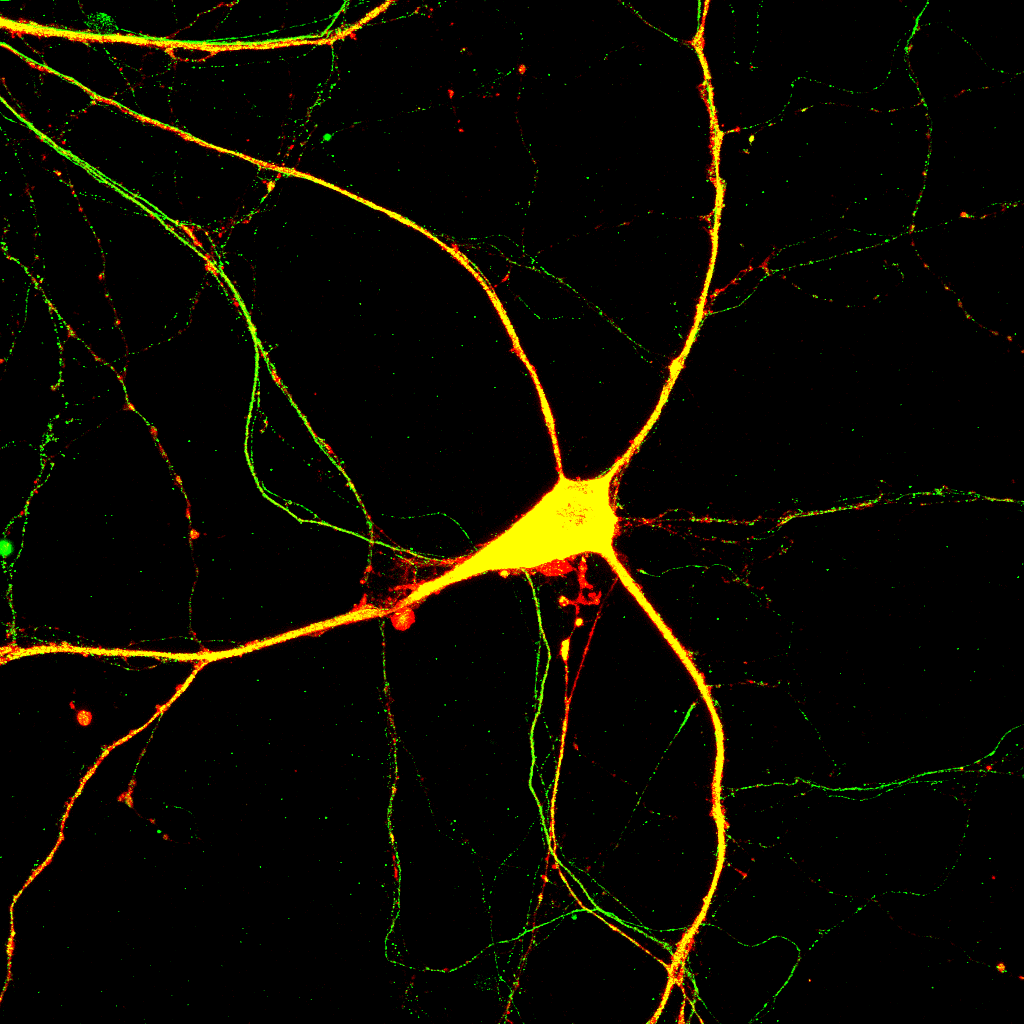

Supplement: Supplementary file 6 — Source data Fig. 5 [file 44319_2026_766_MOESM6_ESM.zip › 5E/122024NMDA RPM -+ Cal in002_3. NMDA004_Processed001.tif]

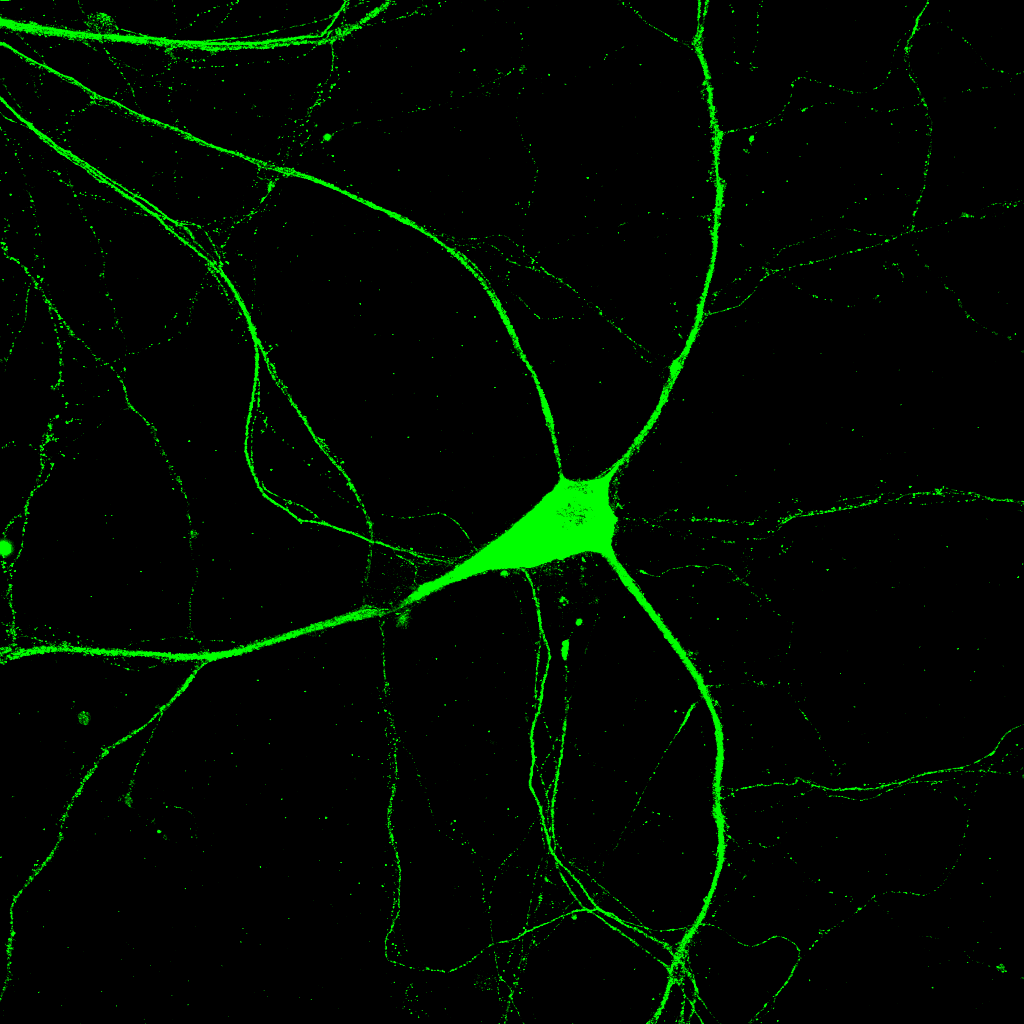

Supplement: Supplementary file 6 — Source data Fig. 5 [file 44319_2026_766_MOESM6_ESM.zip › 5E/122024NMDA RPM -+ Cal in002_3. NMDA004_Processed001_ch00.tif]

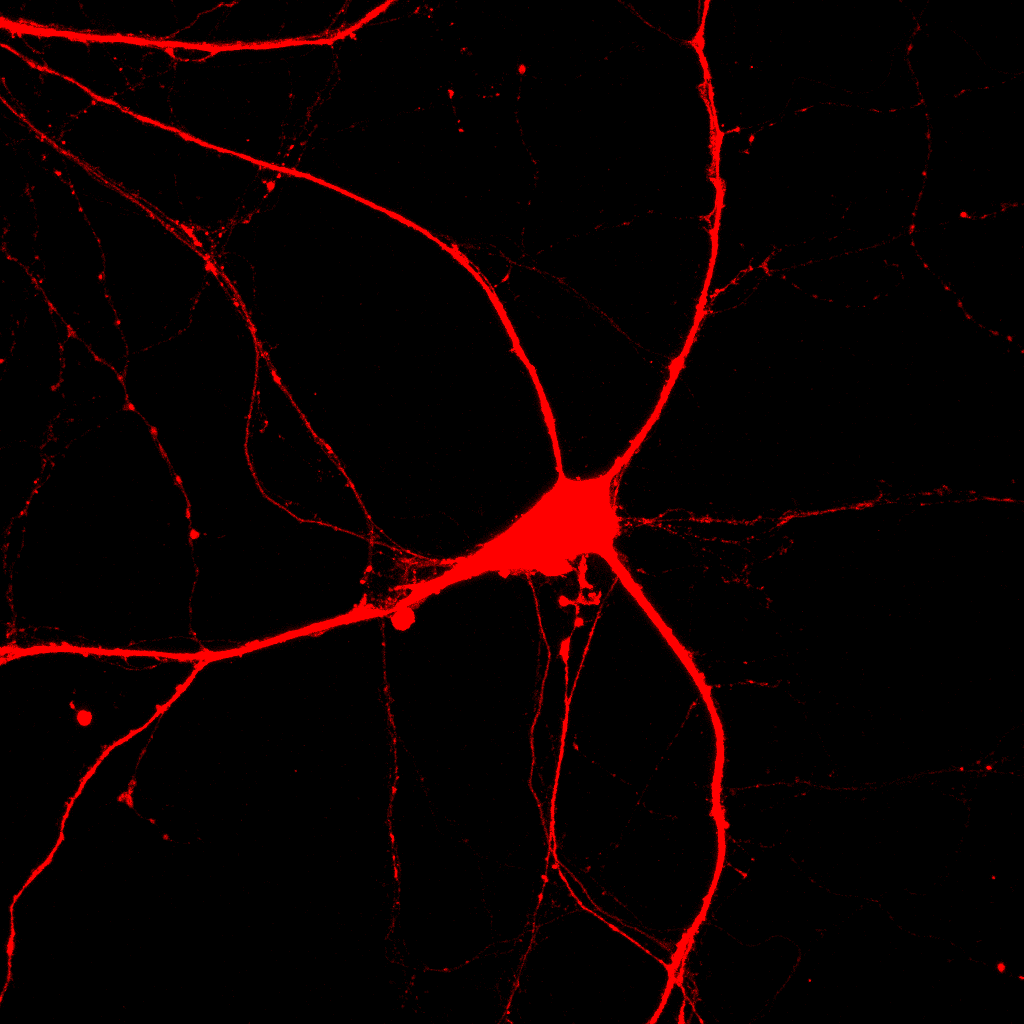

Supplement: Supplementary file 6 — Source data Fig. 5 [file 44319_2026_766_MOESM6_ESM.zip › 5E/122024NMDA RPM -+ Cal in002_3. NMDA004_Processed001_ch01.tif]

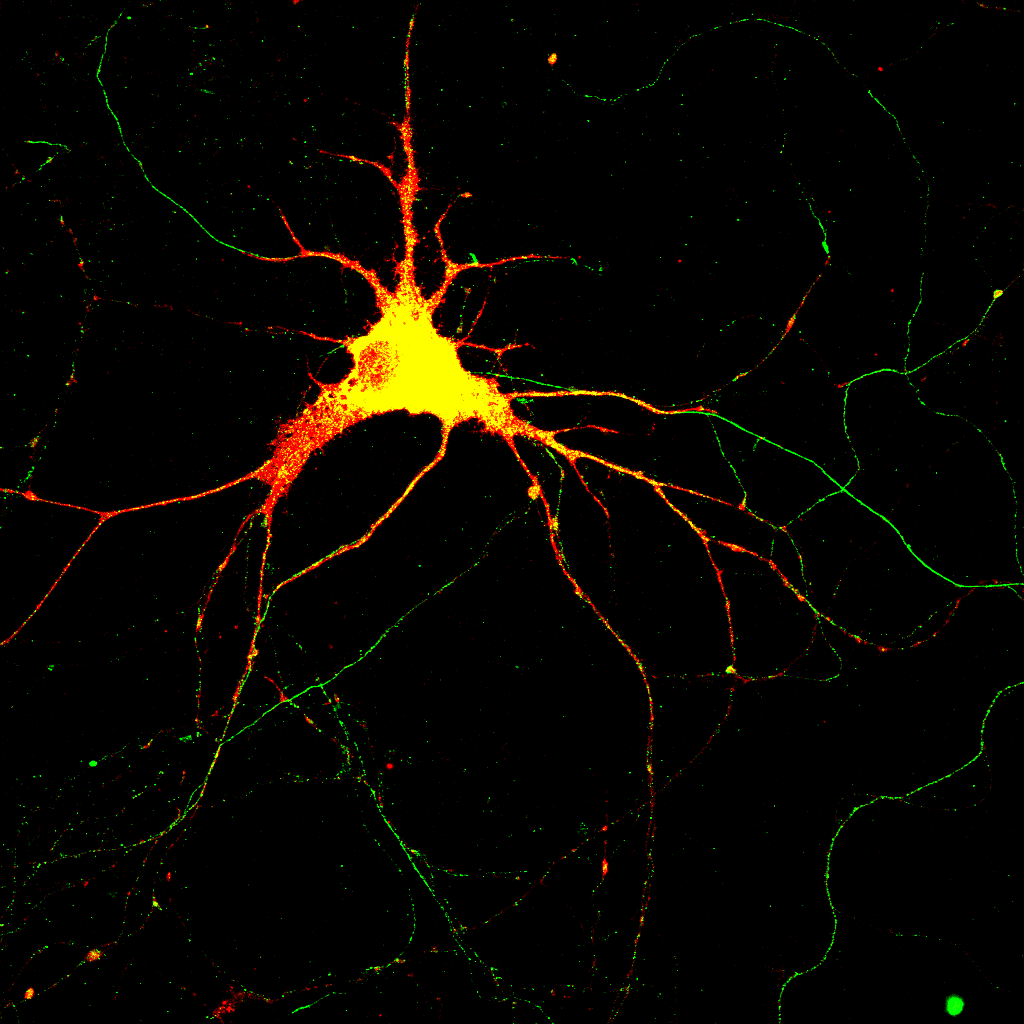

Supplement: Supplementary file 6 — Source data Fig. 5 [file 44319_2026_766_MOESM6_ESM.zip › 5E/122024NMDA RPM -+ Cal in002_4. NMDA+Cal in003_Processed001.tif]

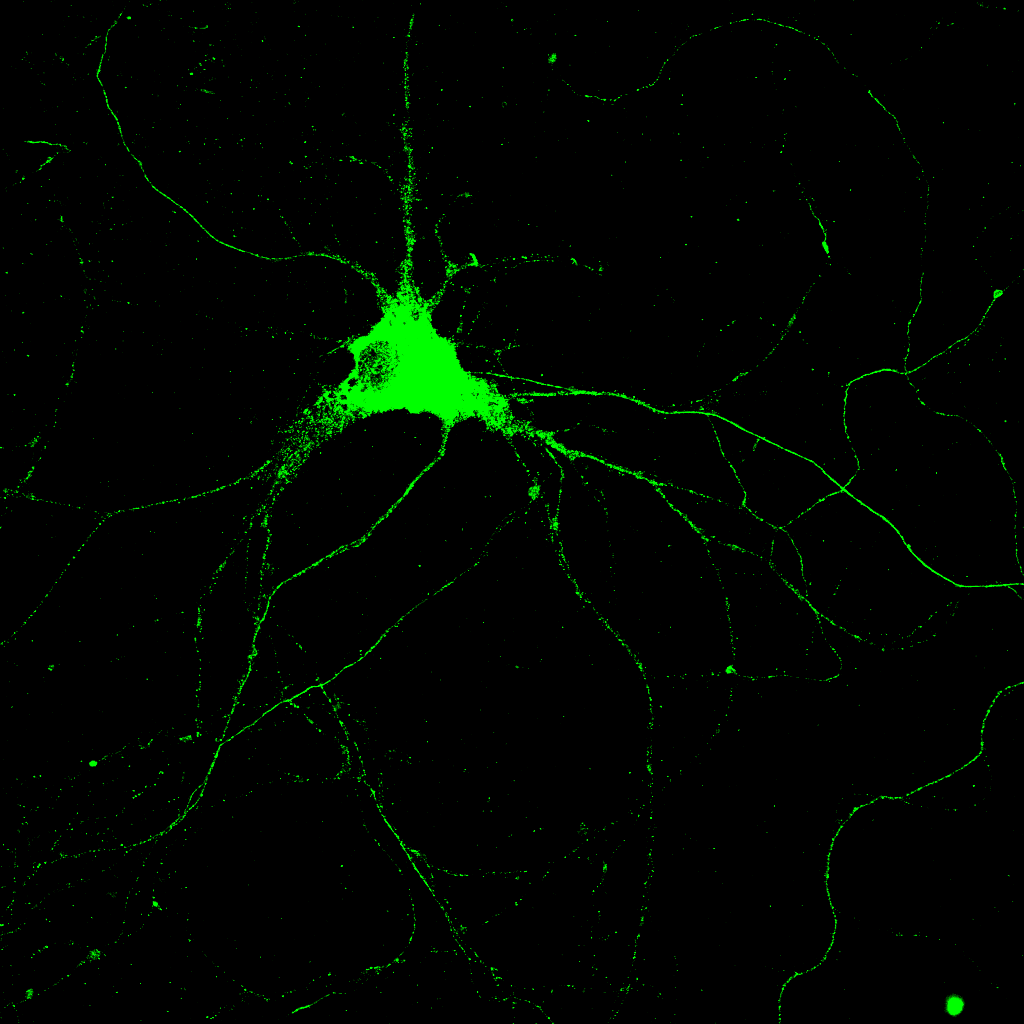

Supplement: Supplementary file 6 — Source data Fig. 5 [file 44319_2026_766_MOESM6_ESM.zip › 5E/122024NMDA RPM -+ Cal in002_4. NMDA+Cal in003_Processed001_ch00.tif]

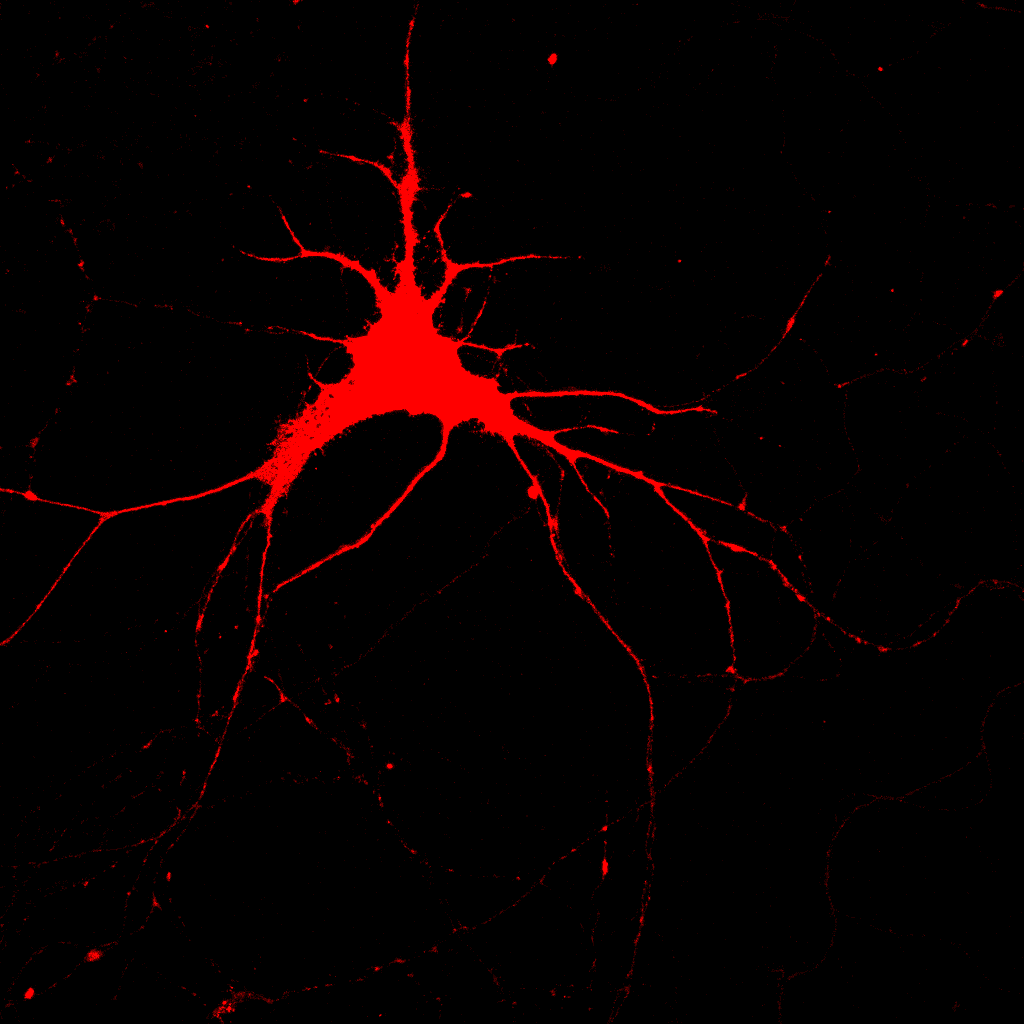

Supplement: Supplementary file 6 — Source data Fig. 5 [file 44319_2026_766_MOESM6_ESM.zip › 5E/122024NMDA RPM -+ Cal in002_4. NMDA+Cal in003_Processed001_ch01.tif]

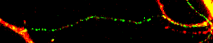

Supplement: Supplementary file 6 — Source data Fig. 5 [file 44319_2026_766_MOESM6_ESM.zip › 5E/Inset/1_1_0000_122024NMDA RPM -+ Cal in002_1. Basal001_Processed001.tif.tif]

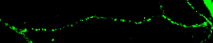

Supplement: Supplementary file 6 — Source data Fig. 5 [file 44319_2026_766_MOESM6_ESM.zip › 5E/Inset/1_1_0001_122024NMDA RPM -+ Cal in002_1. Basal001_Processed001_ch00.tif.tif]

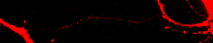

Supplement: Supplementary file 6 — Source data Fig. 5 [file 44319_2026_766_MOESM6_ESM.zip › 5E/Inset/1_1_0002_122024NMDA RPM -+ Cal in002_1. Basal001_Processed001_ch01.tif.tif]

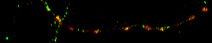

Supplement: Supplementary file 6 — Source data Fig. 5 [file 44319_2026_766_MOESM6_ESM.zip › 5E/Inset/2_new_0000_122024NMDA RPM -+ Cal in002_3. NMDA004_Processed001.tif.tif]

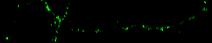

Supplement: Supplementary file 6 — Source data Fig. 5 [file 44319_2026_766_MOESM6_ESM.zip › 5E/Inset/2_new_0001_122024NMDA RPM -+ Cal in002_3. NMDA004_Processed001_ch00.tif.tif]

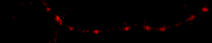

Supplement: Supplementary file 6 — Source data Fig. 5 [file 44319_2026_766_MOESM6_ESM.zip › 5E/Inset/2_new_0002_122024NMDA RPM -+ Cal in002_3. NMDA004_Processed001_ch01.tif.tif]

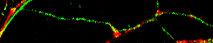

Supplement: Supplementary file 6 — Source data Fig. 5 [file 44319_2026_766_MOESM6_ESM.zip › 5E/Inset/4_1_0000_122024NMDA RPM -+ Cal in002_4. NMDA+Cal in003_Processed001.tif.tif]

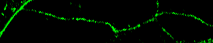

Supplement: Supplementary file 6 — Source data Fig. 5 [file 44319_2026_766_MOESM6_ESM.zip › 5E/Inset/4_1_0001_122024NMDA RPM -+ Cal in002_4. NMDA+Cal in003_Processed001_ch00.tif.tif]

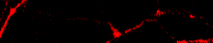

Supplement: Supplementary file 6 — Source data Fig. 5 [file 44319_2026_766_MOESM6_ESM.zip › 5E/Inset/4_1_0002_122024NMDA RPM -+ Cal in002_4. NMDA+Cal in003_Processed001_ch01.tif.tif]

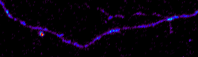

Supplement: Supplementary file 6 — Source data Fig. 5 [file 44319_2026_766_MOESM6_ESM.zip › 5G/1-1_0000_260115 WT vs del PxxP RPM assay002_1. WT_Basal003_Processed001.tif.tif]

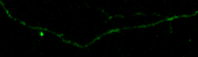

Supplement: Supplementary file 6 — Source data Fig. 5 [file 44319_2026_766_MOESM6_ESM.zip › 5G/1-1_0001_260115 WT vs del PxxP RPM assay002_1. WT_Basal003_Processed001 GREEN.tif.tif]

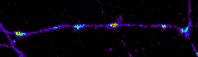

Supplement: Supplementary file 6 — Source data Fig. 5 [file 44319_2026_766_MOESM6_ESM.zip › 5G/2_0000_260115 WT vs del PxxP RPM assay002_1. WT_NMDA002_Processed001.tif.tif]

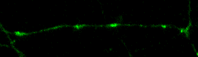

Supplement: Supplementary file 6 — Source data Fig. 5 [file 44319_2026_766_MOESM6_ESM.zip › 5G/2_0001_260115 WT vs del PxxP RPM assay002_1. WT_NMDA002_Processed001Green.tif.tif]

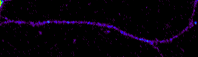

Supplement: Supplementary file 6 — Source data Fig. 5 [file 44319_2026_766_MOESM6_ESM.zip › 5G/3_0000_260115 WT vs del PxxP RPM assay002_2. DelPXXP_Basal002_Processed001.tif.tif]

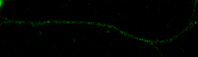

Supplement: Supplementary file 6 — Source data Fig. 5 [file 44319_2026_766_MOESM6_ESM.zip › 5G/3_0001_260115 WT vs del PxxP RPM assay002_2. DelPXXP_Basal002_Processed001G.tif.tif]

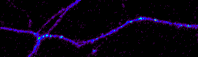

Supplement: Supplementary file 6 — Source data Fig. 5 [file 44319_2026_766_MOESM6_ESM.zip › 5G/4_0000_260115 WT vs del PxxP RPM assay002_2. DelPXXP_NMDA002_Processed001.tif.tif]

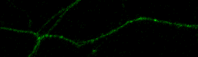

Supplement: Supplementary file 6 — Source data Fig. 5 [file 44319_2026_766_MOESM6_ESM.zip › 5G/4_0001_260115 WT vs del PxxP RPM assay002_2. DelPXXP_NMDA002_Processed001G.tif.tif]

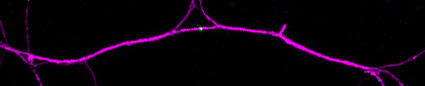

Supplement: Supplementary file 6 — Source data Fig. 5 [file 44319_2026_766_MOESM6_ESM.zip › 5I/Inset/0_1_0000_Puro-mTOR PLA_NMDA-cal_Axon only_0. No Puro001_Processed001.tif.tif]

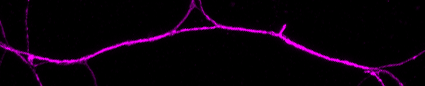

Supplement: Supplementary file 6 — Source data Fig. 5 [file 44319_2026_766_MOESM6_ESM.zip › 5I/Inset/0_1_0001_Puro-mTOR PLA_NMDA-cal_Axon only_0. No Puro001_Processed001_ch00.tif.tif]

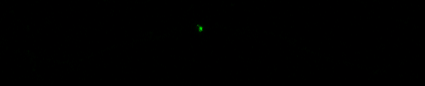

Supplement: Supplementary file 6 — Source data Fig. 5 [file 44319_2026_766_MOESM6_ESM.zip › 5I/Inset/0_1_0002_Puro-mTOR PLA_NMDA-cal_Axon only_0. No Puro001_Processed001_ch01.tif.tif]

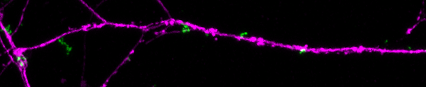

Supplement: Supplementary file 6 — Source data Fig. 5 [file 44319_2026_766_MOESM6_ESM.zip › 5I/Inset/1_1_0000_Puro-mTOR PLA_NMDA-cal_Axon only_1. Basal001_Processed001.tif.tif]

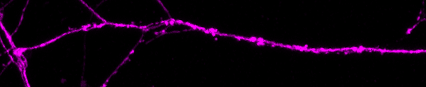

Supplement: Supplementary file 6 — Source data Fig. 5 [file 44319_2026_766_MOESM6_ESM.zip › 5I/Inset/1_1_0001_Puro-mTOR PLA_NMDA-cal_Axon only_1. Basal001_Processed001_ch00.tif.tif]

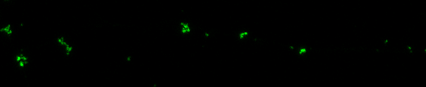

Supplement: Supplementary file 6 — Source data Fig. 5 [file 44319_2026_766_MOESM6_ESM.zip › 5I/Inset/1_1_0002_Puro-mTOR PLA_NMDA-cal_Axon only_1. Basal001_Processed001_ch01.tif.tif]

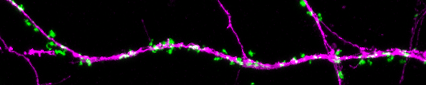

Supplement: Supplementary file 6 — Source data Fig. 5 [file 44319_2026_766_MOESM6_ESM.zip › 5I/Inset/2_0000_Puro-mTOR PLA_NMDA-cal_Axon only_2. NMDA001_Processed001.tif.tif]

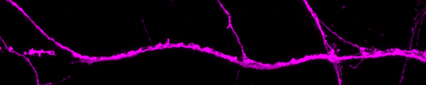

Supplement: Supplementary file 6 — Source data Fig. 5 [file 44319_2026_766_MOESM6_ESM.zip › 5I/Inset/2_0001_Puro-mTOR PLA_NMDA-cal_Axon only_2. NMDA001_Processed001_ch00.tif.tif]

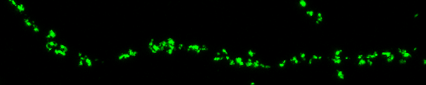

Supplement: Supplementary file 6 — Source data Fig. 5 [file 44319_2026_766_MOESM6_ESM.zip › 5I/Inset/2_0002_Puro-mTOR PLA_NMDA-cal_Axon only_2. NMDA001_Processed001_ch01.tif.tif]

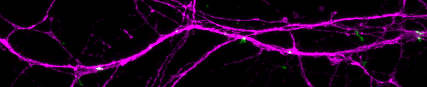

Supplement: Supplementary file 6 — Source data Fig. 5 [file 44319_2026_766_MOESM6_ESM.zip › 5I/Inset/3_0000_Puro-mTOR PLA_NMDA-cal_Axon only_4. CalIn_NMDA002_Processed001.tif.tif]

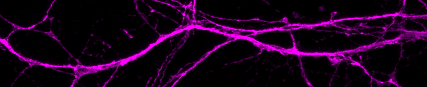

Supplement: Supplementary file 6 — Source data Fig. 5 [file 44319_2026_766_MOESM6_ESM.zip › 5I/Inset/3_0001_Puro-mTOR PLA_NMDA-cal_Axon only_4. CalIn_NMDA002_Processed001_ch00.tif.tif]

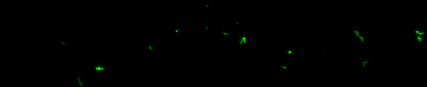

Supplement: Supplementary file 6 — Source data Fig. 5 [file 44319_2026_766_MOESM6_ESM.zip › 5I/Inset/3_0002_Puro-mTOR PLA_NMDA-cal_Axon only_4. CalIn_NMDA002_Processed001_ch01.tif.tif]

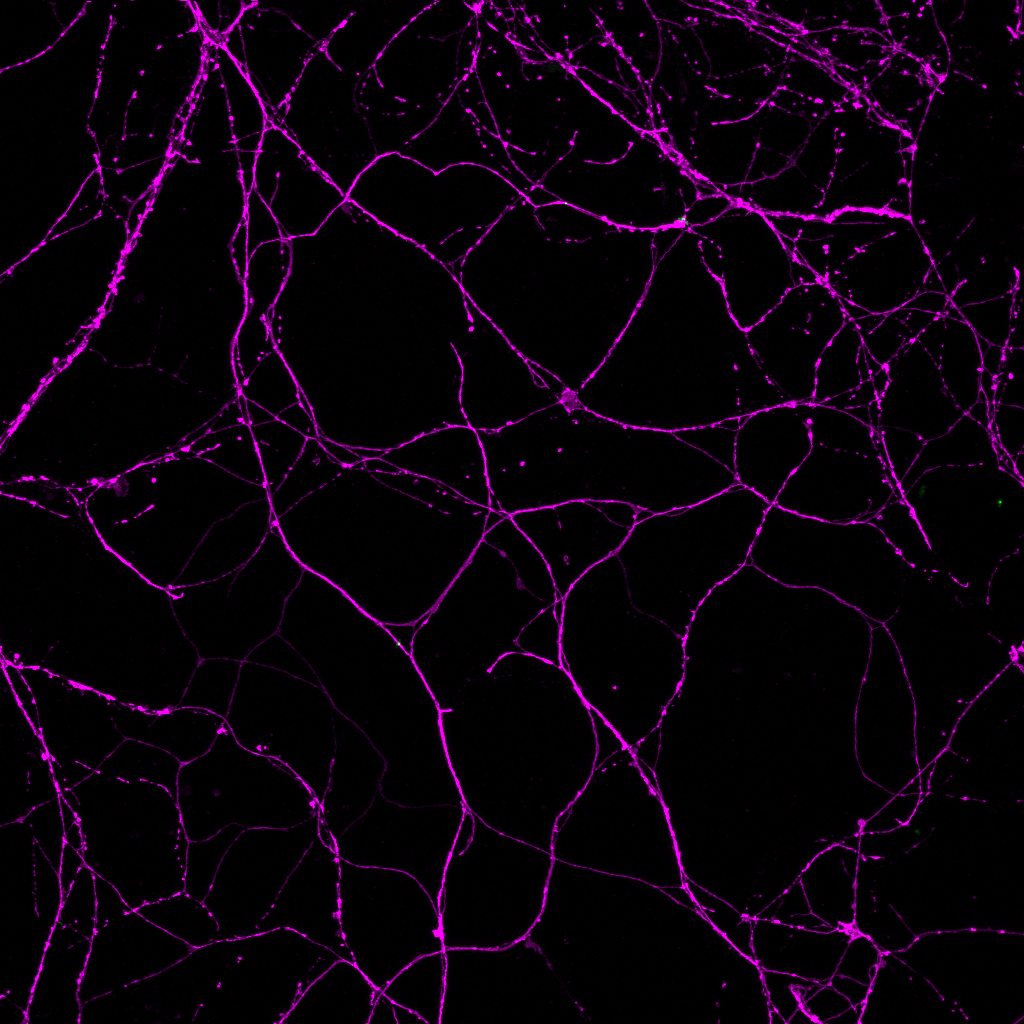

Supplement: Supplementary file 6 — Source data Fig. 5 [file 44319_2026_766_MOESM6_ESM.zip › 5I/Puro-mTOR PLA_NMDA-cal_Axon only_0. No Puro001_Processed001.tif]

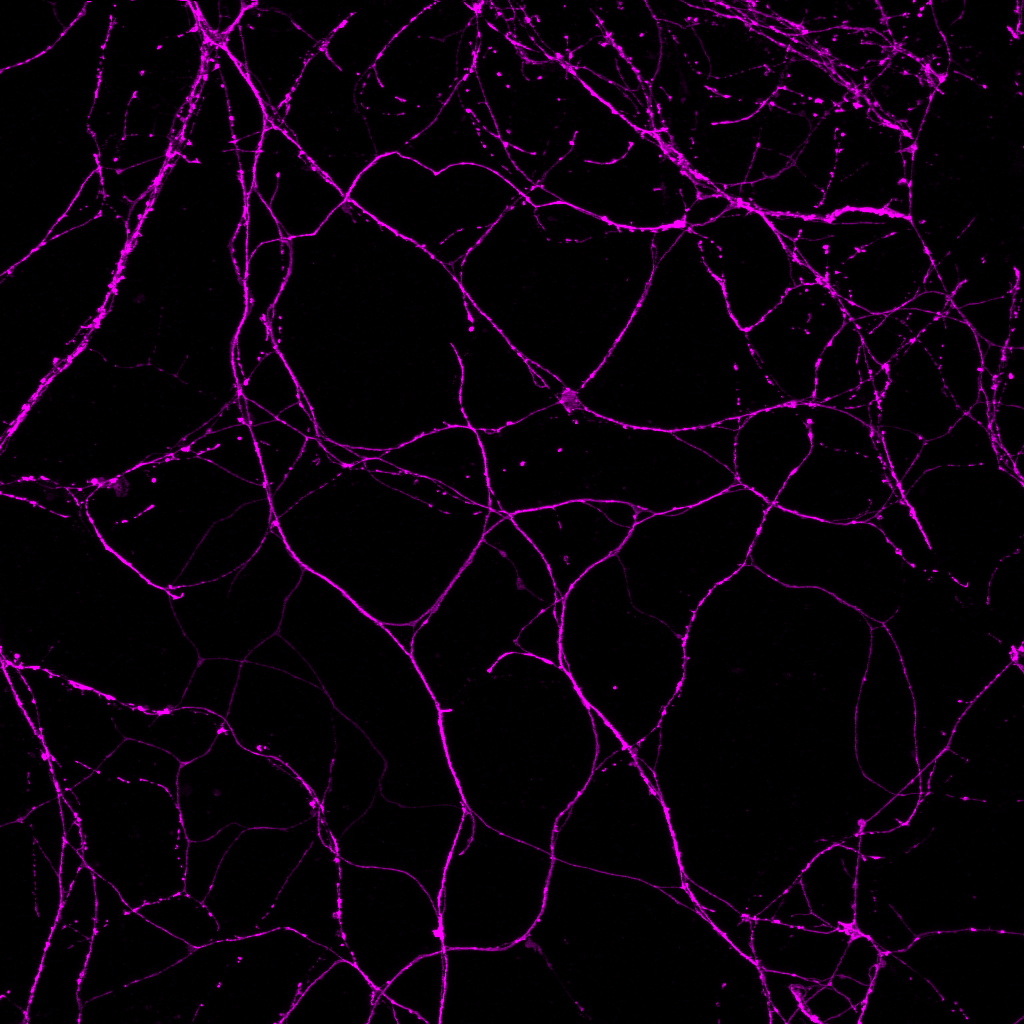

Supplement: Supplementary file 6 — Source data Fig. 5 [file 44319_2026_766_MOESM6_ESM.zip › 5I/Puro-mTOR PLA_NMDA-cal_Axon only_0. No Puro001_Processed001_ch00.tif]

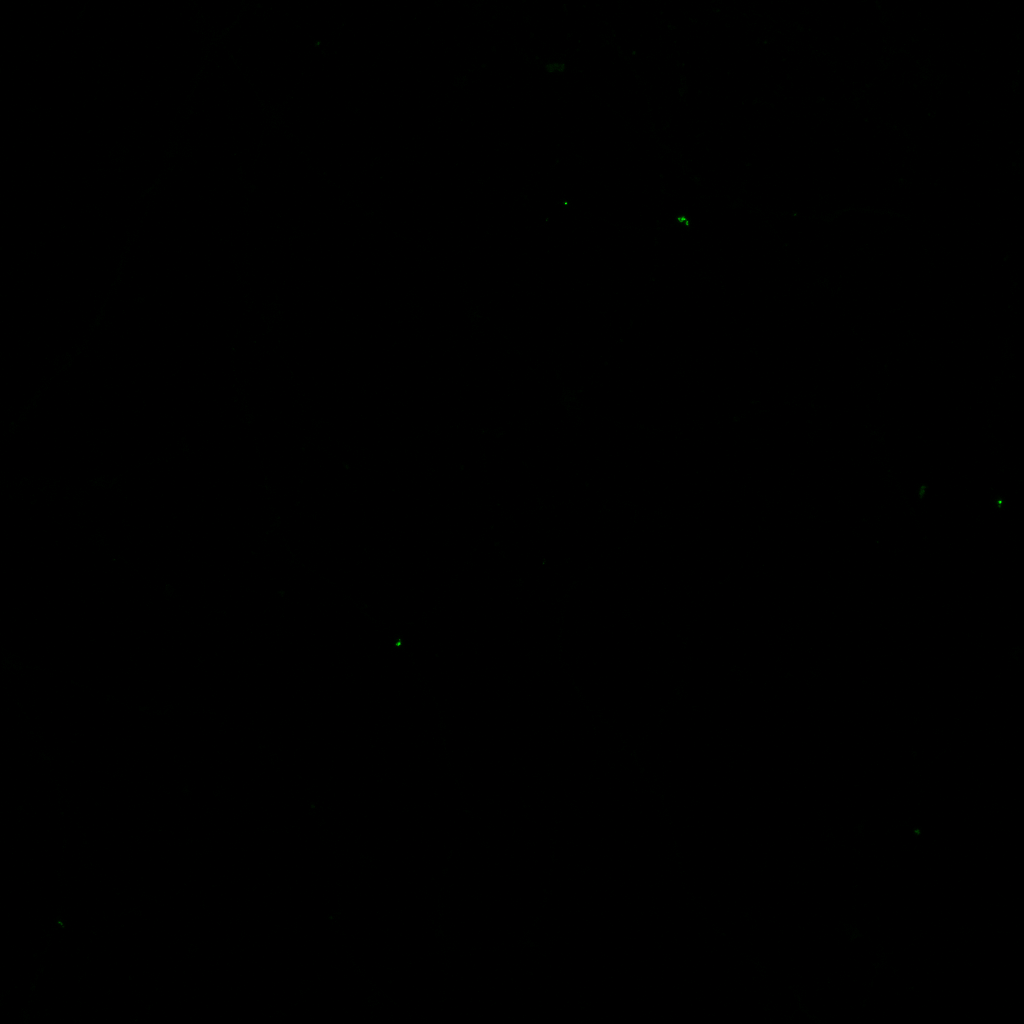

Supplement: Supplementary file 6 — Source data Fig. 5 [file 44319_2026_766_MOESM6_ESM.zip › 5I/Puro-mTOR PLA_NMDA-cal_Axon only_0. No Puro001_Processed001_ch01.tif]

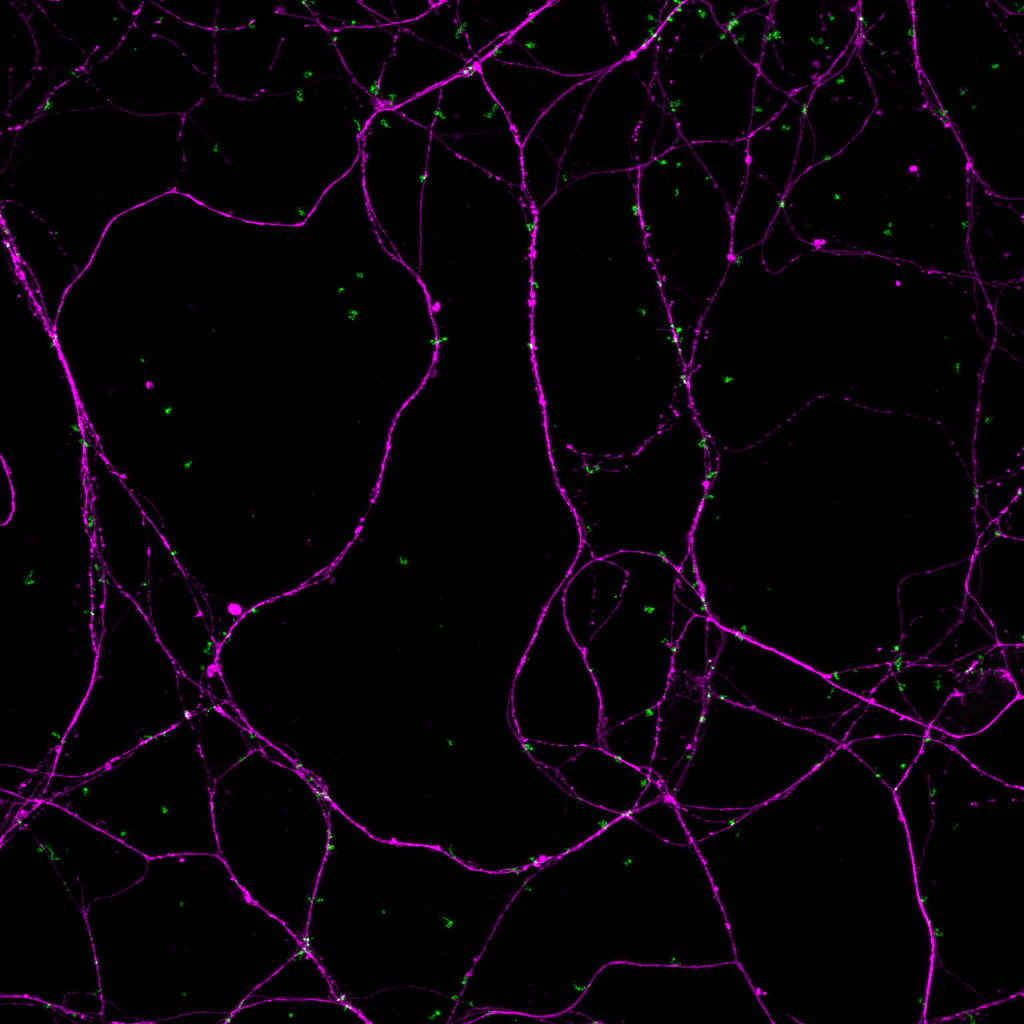

Supplement: Supplementary file 6 — Source data Fig. 5 [file 44319_2026_766_MOESM6_ESM.zip › 5I/Puro-mTOR PLA_NMDA-cal_Axon only_1. Basal001_Processed001.tif]

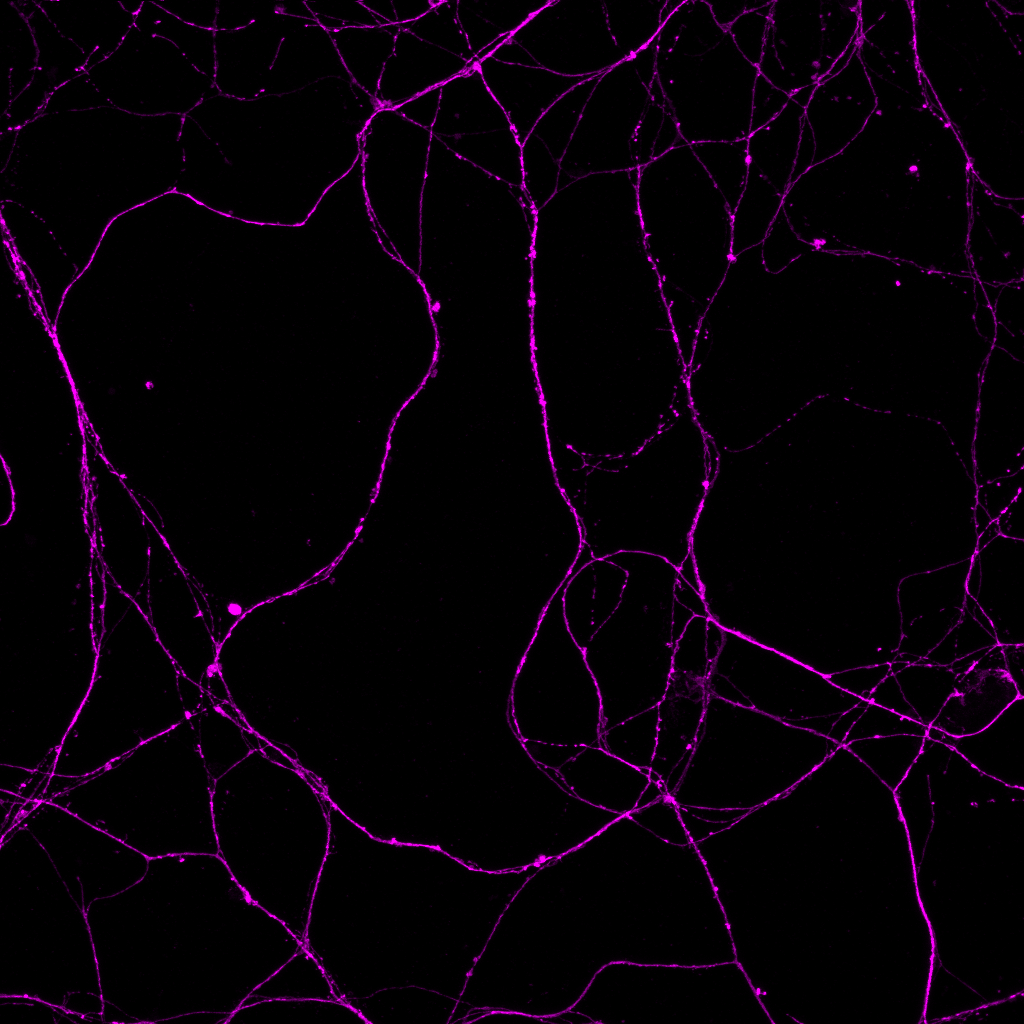

Supplement: Supplementary file 6 — Source data Fig. 5 [file 44319_2026_766_MOESM6_ESM.zip › 5I/Puro-mTOR PLA_NMDA-cal_Axon only_1. Basal001_Processed001_ch00.tif]

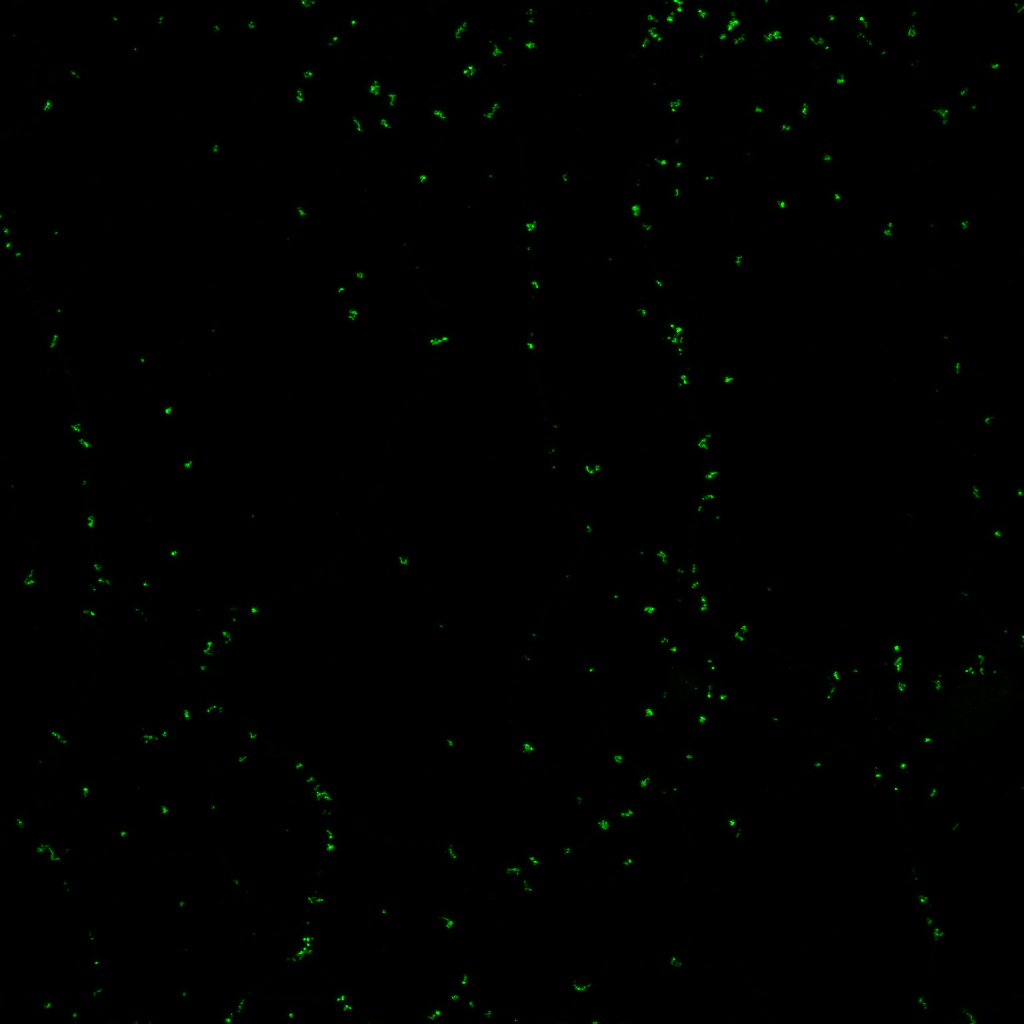

Supplement: Supplementary file 6 — Source data Fig. 5 [file 44319_2026_766_MOESM6_ESM.zip › 5I/Puro-mTOR PLA_NMDA-cal_Axon only_1. Basal001_Processed001_ch01.tif]

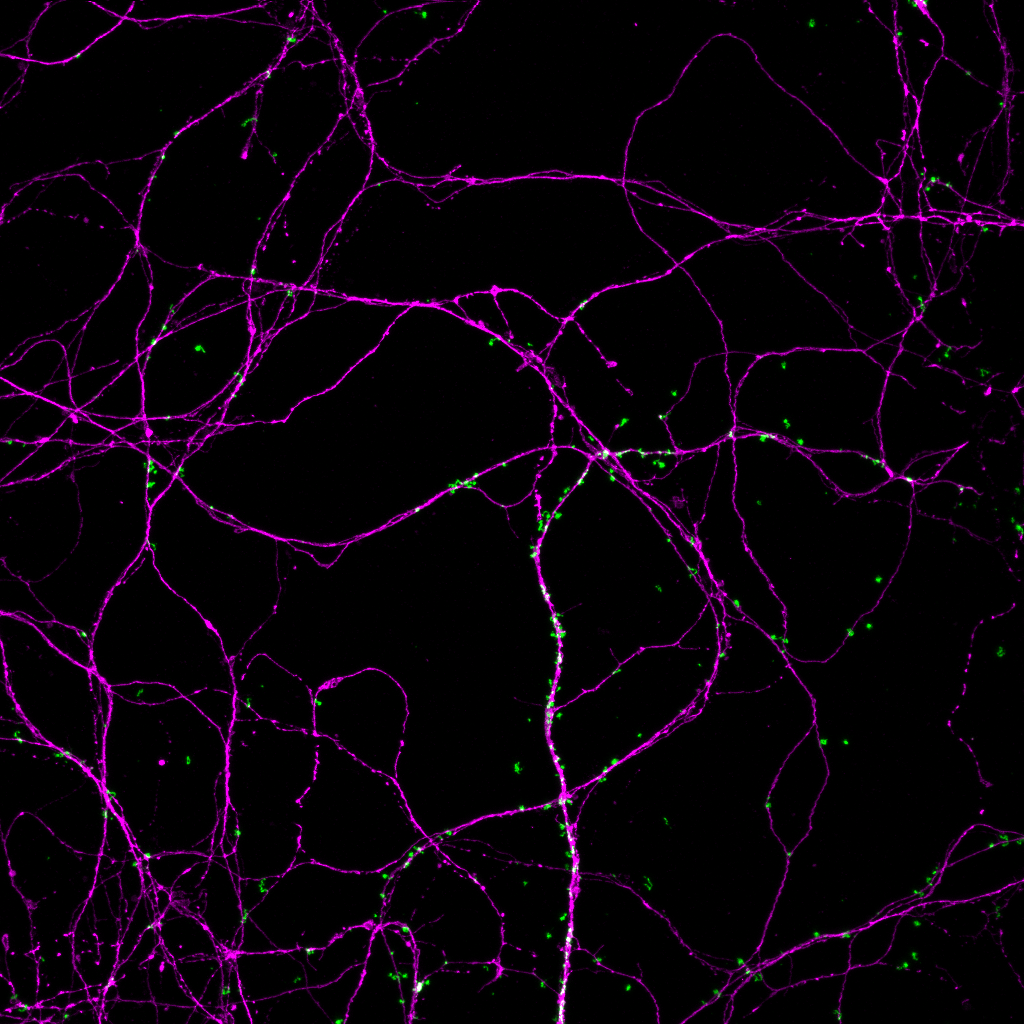

Supplement: Supplementary file 6 — Source data Fig. 5 [file 44319_2026_766_MOESM6_ESM.zip › 5I/Puro-mTOR PLA_NMDA-cal_Axon only_2. NMDA001_Processed001.tif]

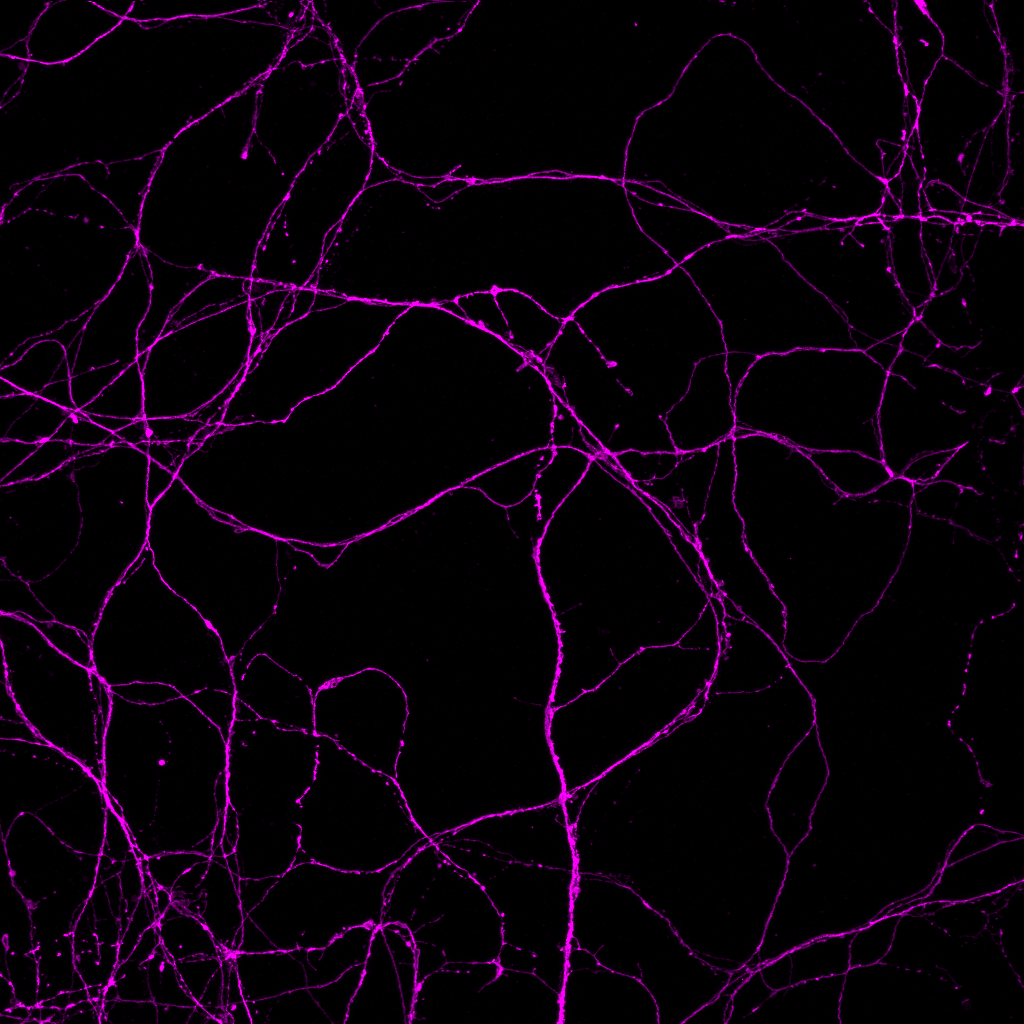

Supplement: Supplementary file 6 — Source data Fig. 5 [file 44319_2026_766_MOESM6_ESM.zip › 5I/Puro-mTOR PLA_NMDA-cal_Axon only_2. NMDA001_Processed001_ch00.tif]

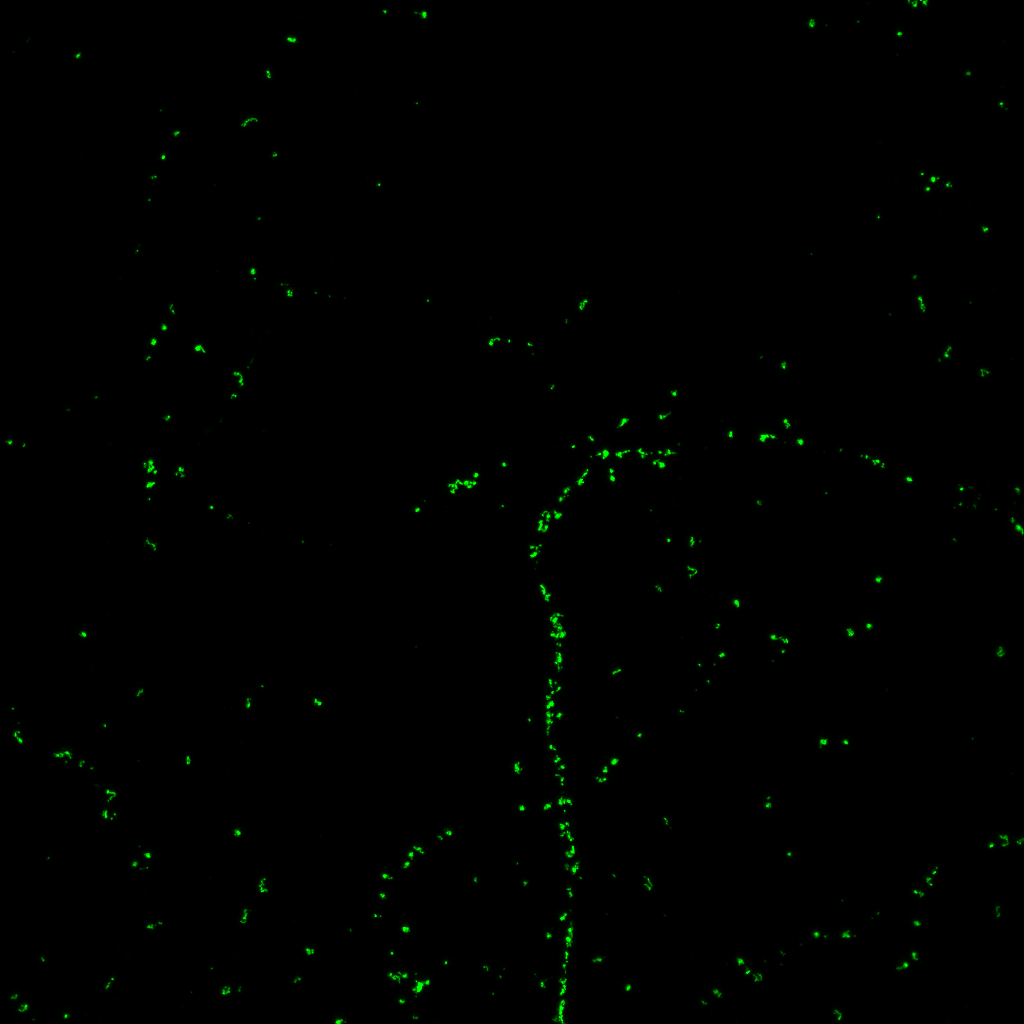

Supplement: Supplementary file 6 — Source data Fig. 5 [file 44319_2026_766_MOESM6_ESM.zip › 5I/Puro-mTOR PLA_NMDA-cal_Axon only_2. NMDA001_Processed001_ch01.tif]

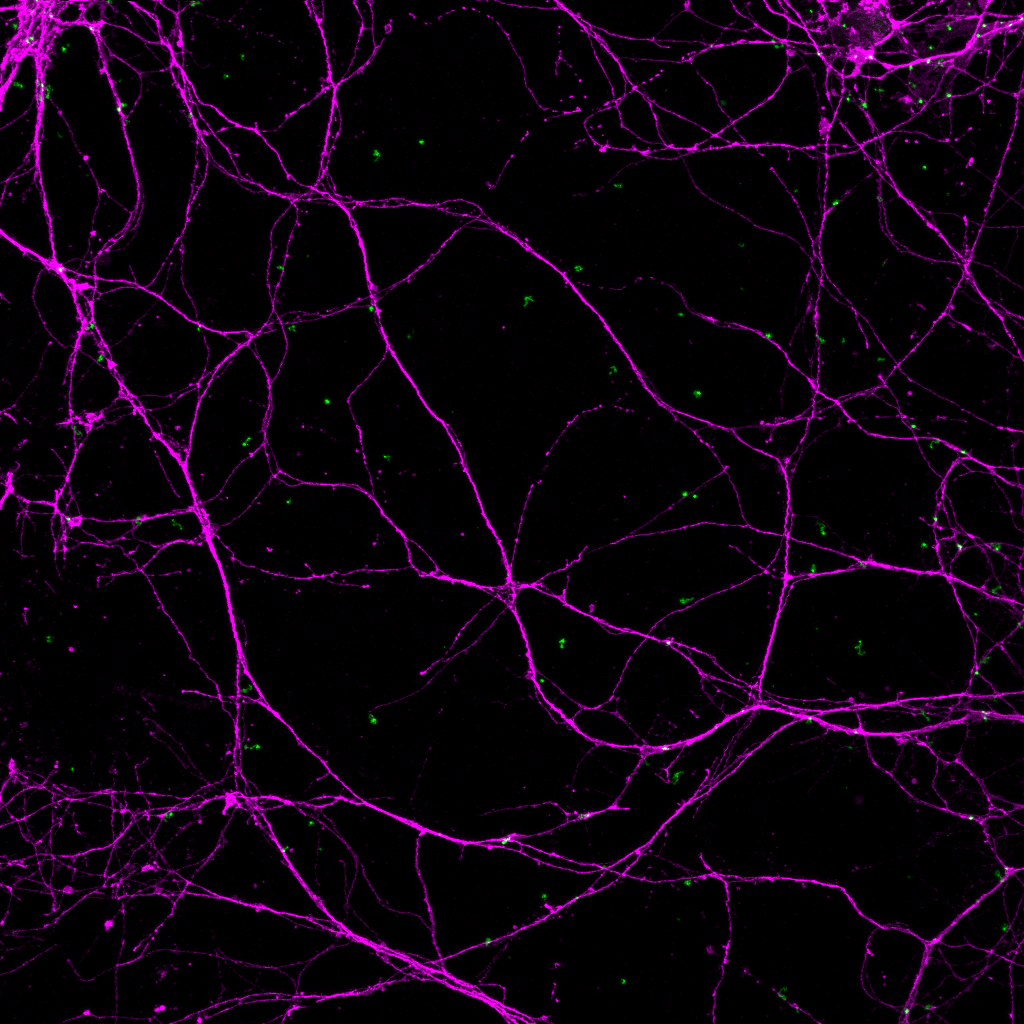

Supplement: Supplementary file 6 — Source data Fig. 5 [file 44319_2026_766_MOESM6_ESM.zip › 5I/Puro-mTOR PLA_NMDA-cal_Axon only_4. CalIn_NMDA002_Processed001.tif]

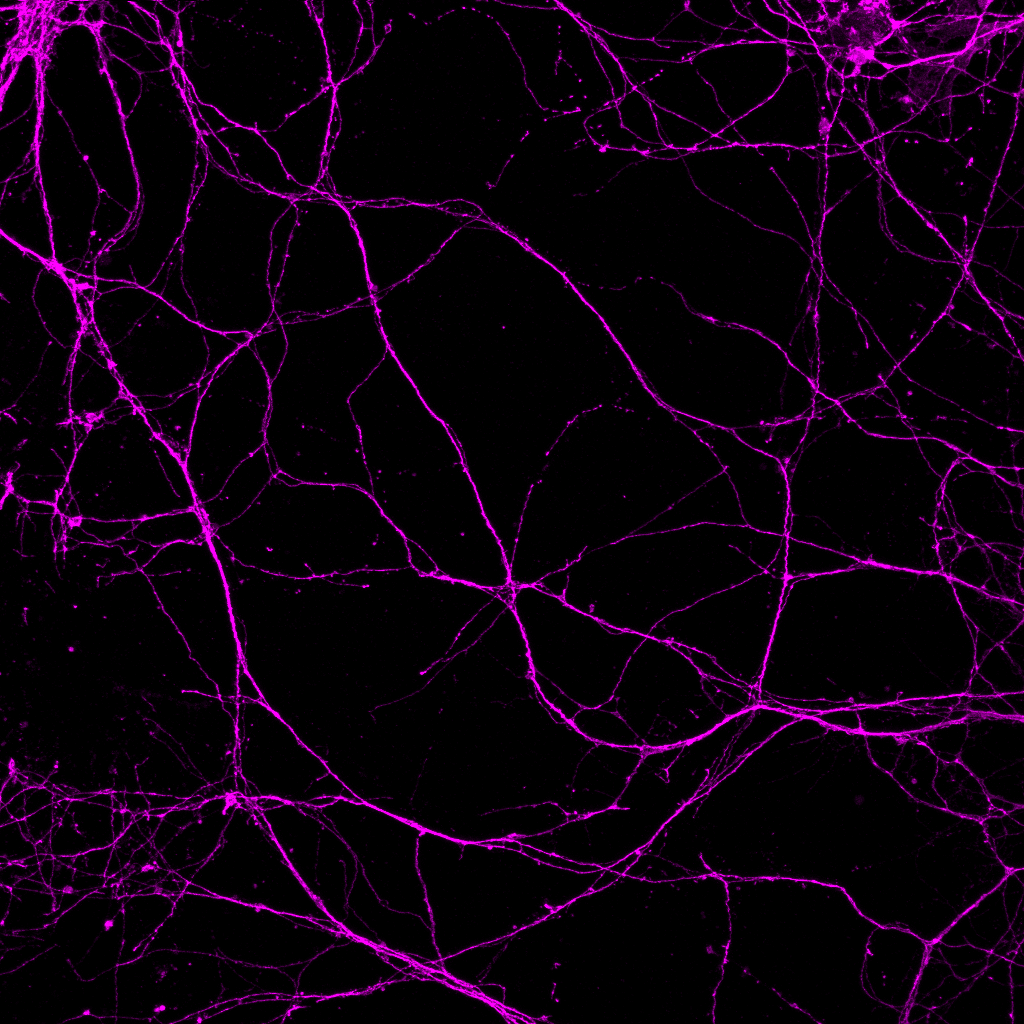

Supplement: Supplementary file 6 — Source data Fig. 5 [file 44319_2026_766_MOESM6_ESM.zip › 5I/Puro-mTOR PLA_NMDA-cal_Axon only_4. CalIn_NMDA002_Processed001_ch00.tif]

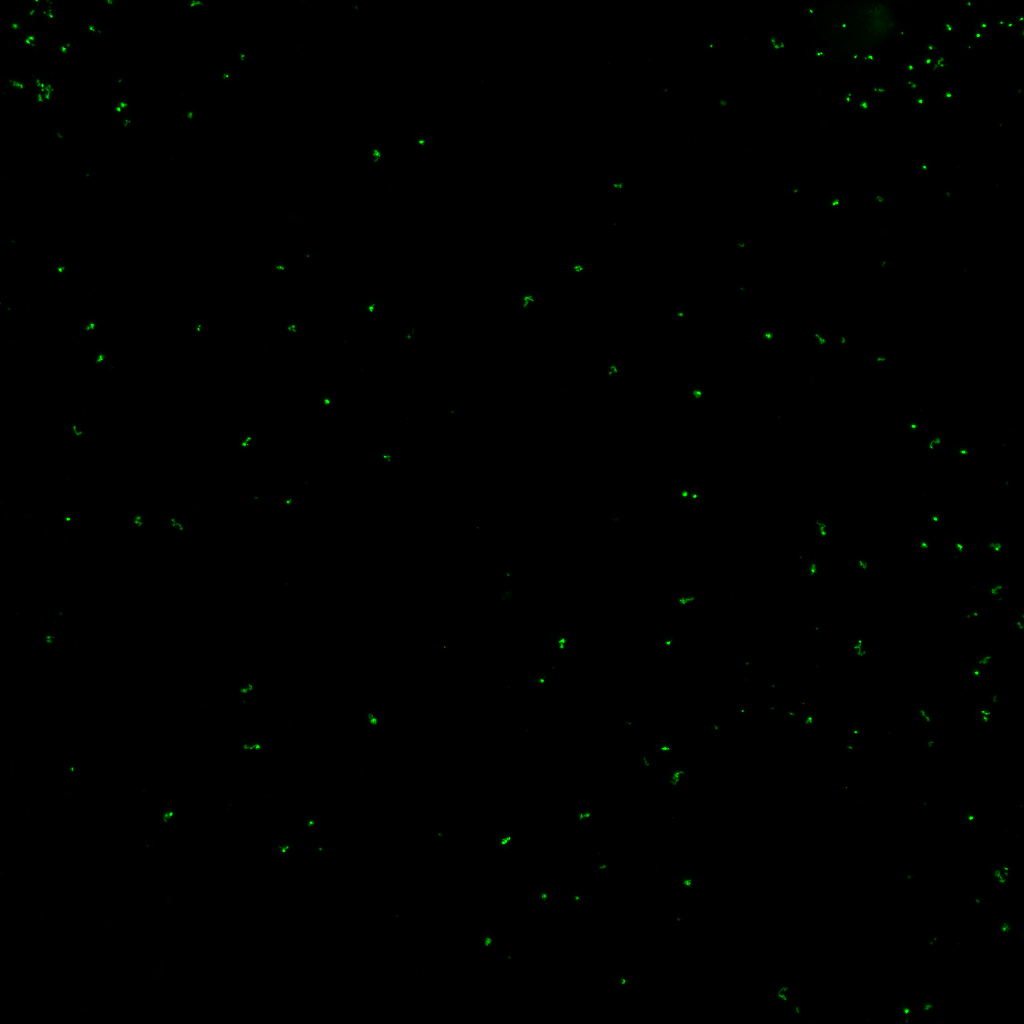

Supplement: Supplementary file 6 — Source data Fig. 5 [file 44319_2026_766_MOESM6_ESM.zip › 5I/Puro-mTOR PLA_NMDA-cal_Axon only_4. CalIn_NMDA002_Processed001_ch01.tif]

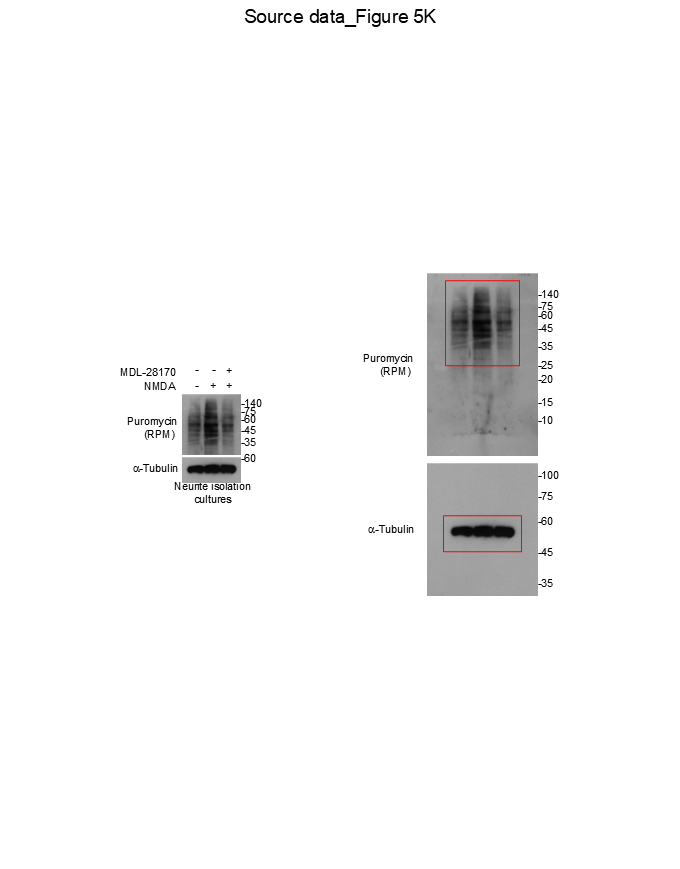

Supplement: Supplementary file 6 — Source data Fig. 5 [file 44319_2026_766_MOESM6_ESM.zip › 5K/Figure5K_Blots.TIF]

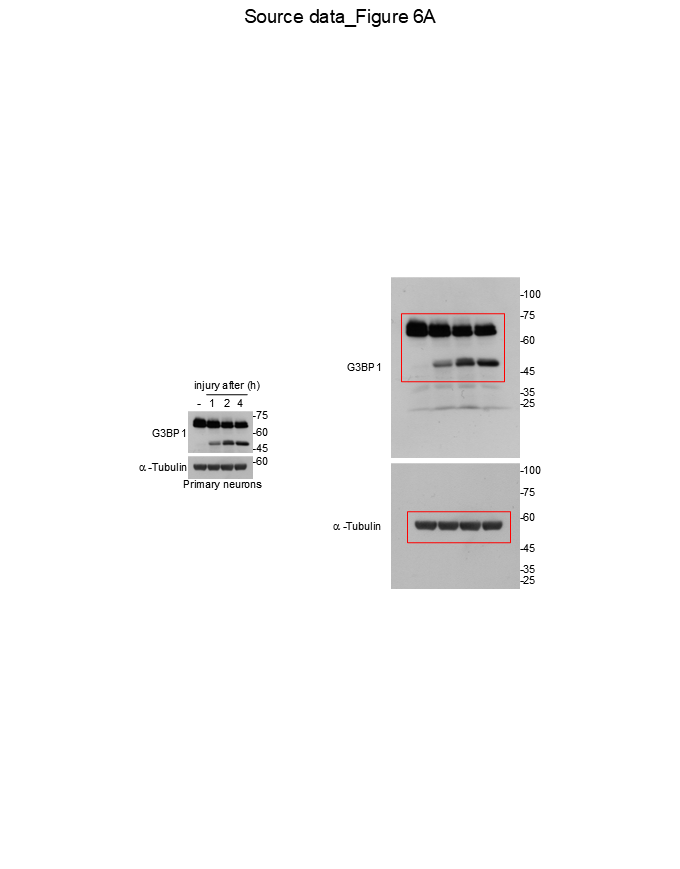

Supplement: Supplementary file 7 — Source data Fig. 6 [file 44319_2026_766_MOESM7_ESM.zip › 6A/Figure6A_Blots.TIF]

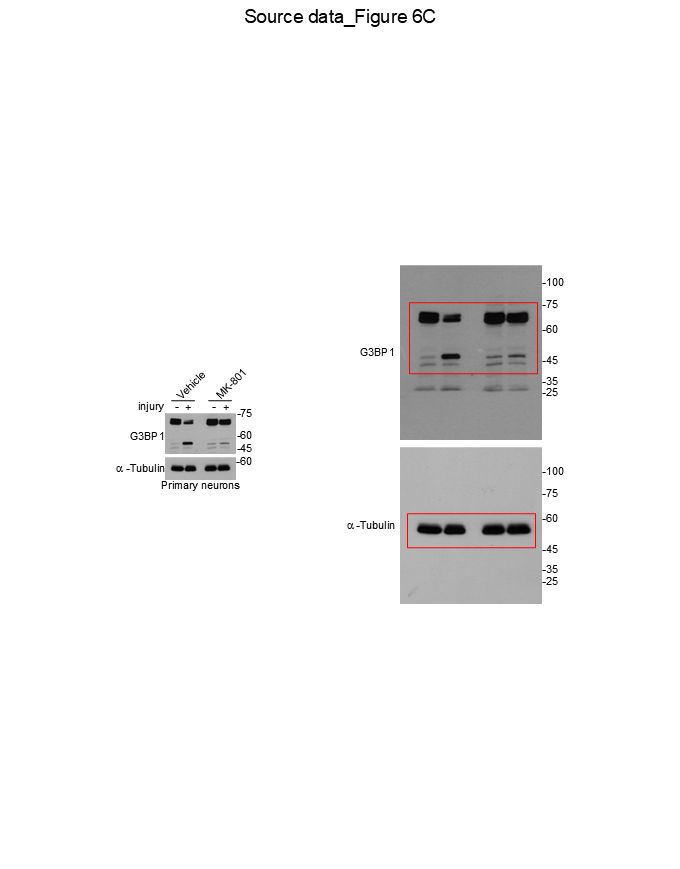

Supplement: Supplementary file 7 — Source data Fig. 6 [file 44319_2026_766_MOESM7_ESM.zip › 6C/Figure6C_Blots.TIF]

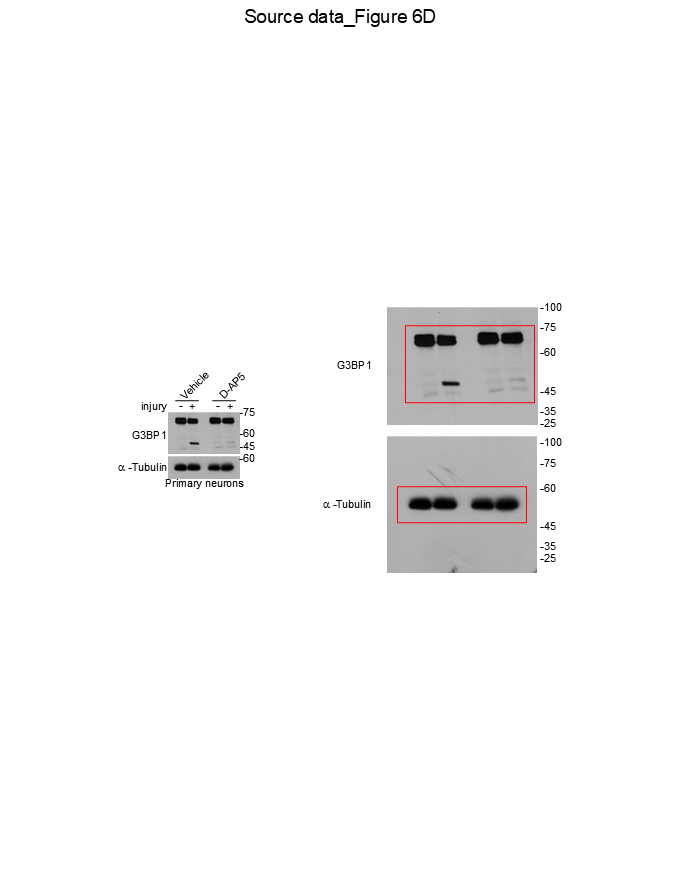

Supplement: Supplementary file 7 — Source data Fig. 6 [file 44319_2026_766_MOESM7_ESM.zip › 6D/Figure6D_Blots.TIF]

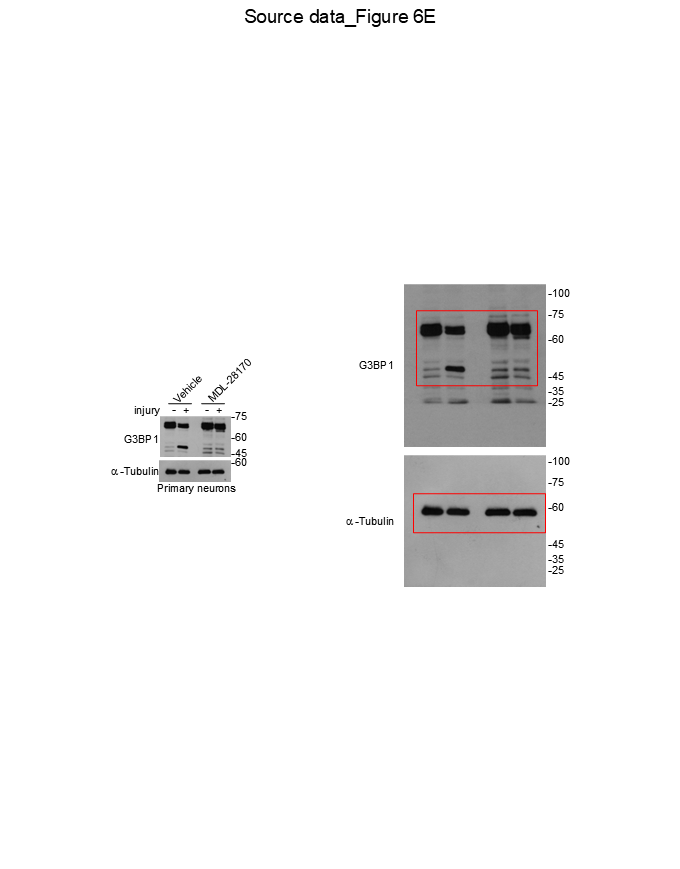

Supplement: Supplementary file 7 — Source data Fig. 6 [file 44319_2026_766_MOESM7_ESM.zip › 6E/Figure6E_Blots.TIF]

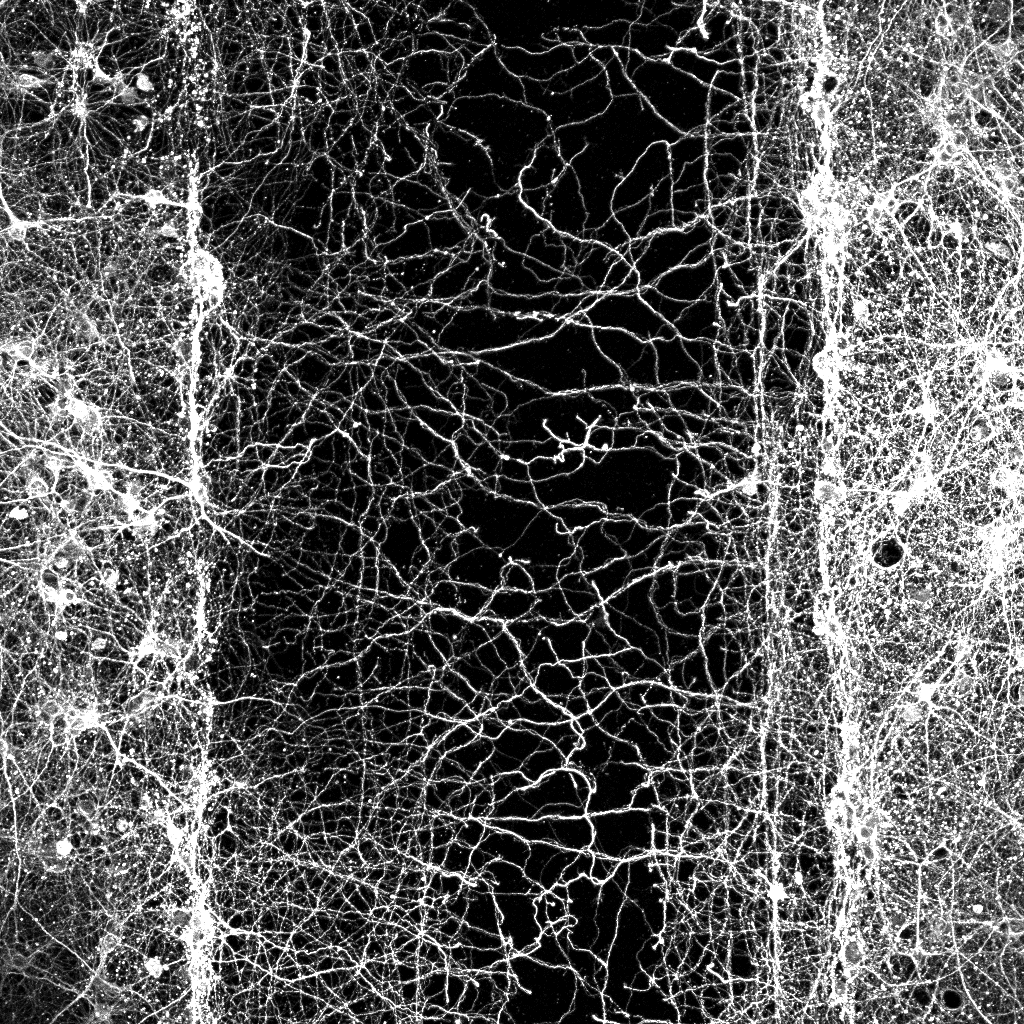

Supplement: Supplementary file 7 — Source data Fig. 6 [file 44319_2026_766_MOESM7_ESM.zip › 6F/251024_axon regen 48 hours002MERGED_1. Control001_Processed001_ch01.tif]

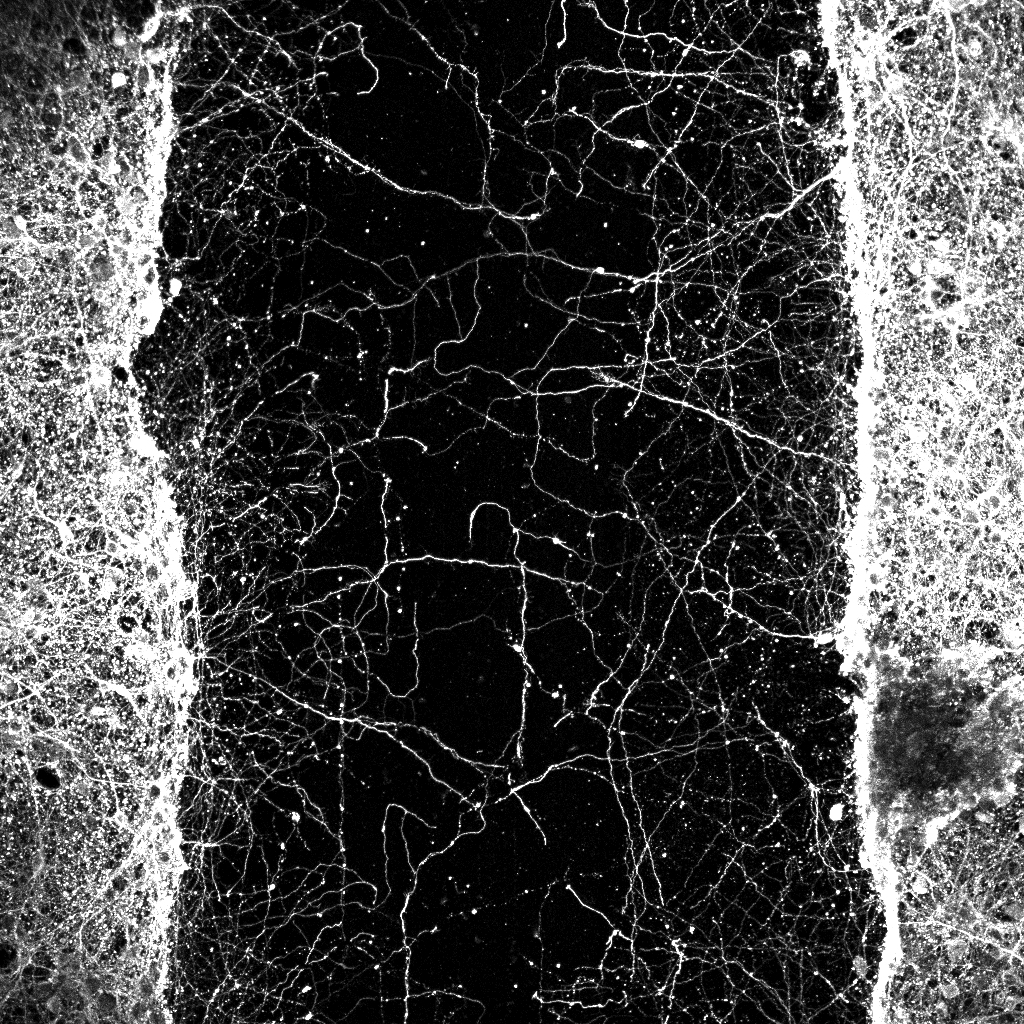

Supplement: Supplementary file 7 — Source data Fig. 6 [file 44319_2026_766_MOESM7_ESM.zip › 6F/251024_axon regen 48 hours002MERGED_2. Torin004_Processed001_ch01.tif]

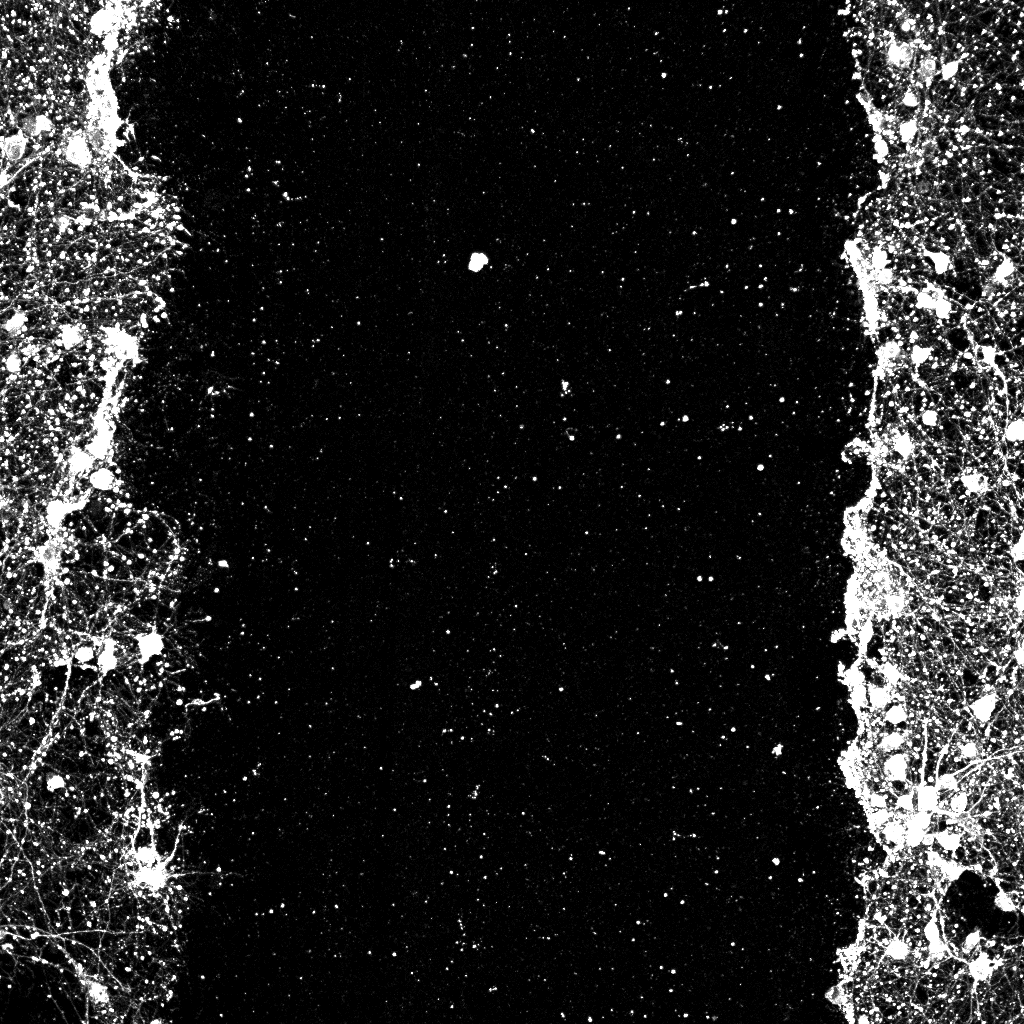

Supplement: Supplementary file 7 — Source data Fig. 6 [file 44319_2026_766_MOESM7_ESM.zip › 6F/251024_axon regen 48 hours002MERGED_3. EGTA003_Processed001_ch01.tif]

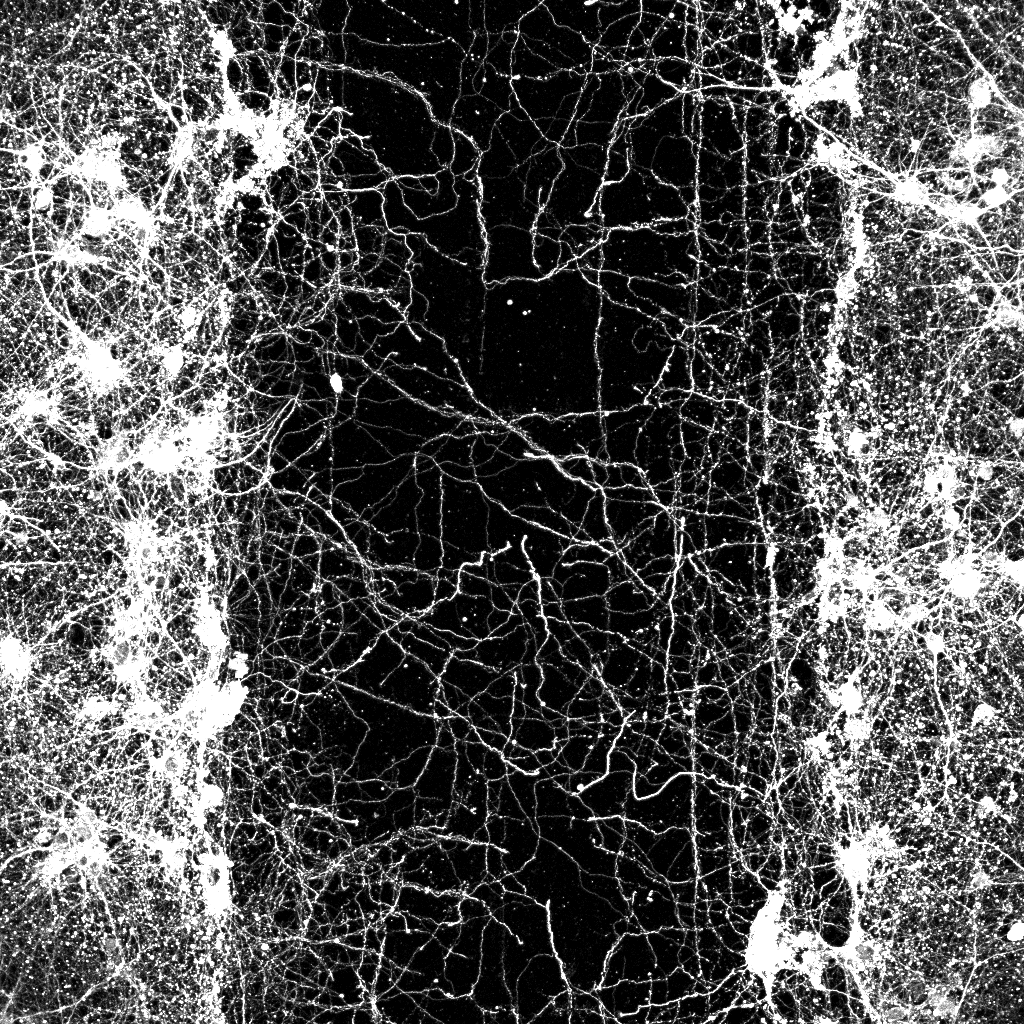

Supplement: Supplementary file 7 — Source data Fig. 6 [file 44319_2026_766_MOESM7_ESM.zip › 6F/251024_axon regen 48 hours002MERGED_4. MDL005_Processed001_ch01.tif]

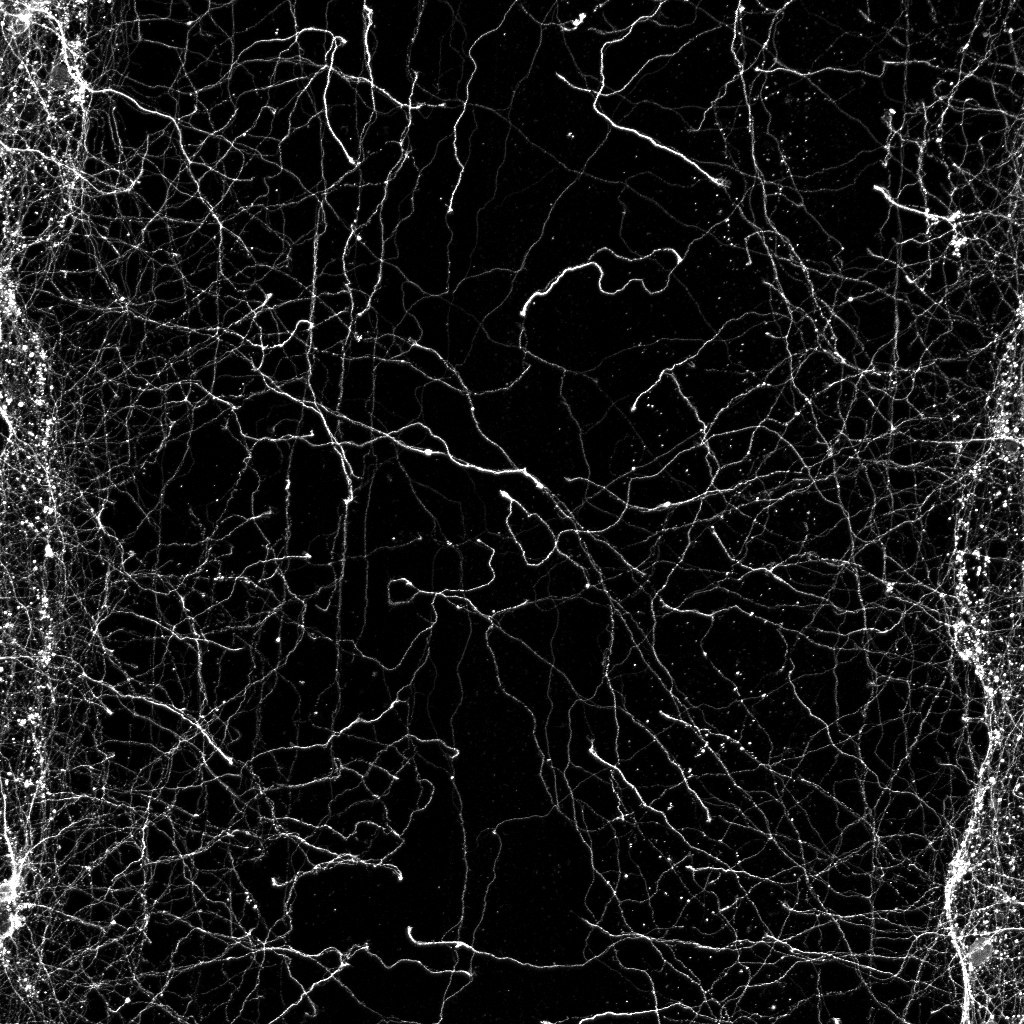

Supplement: Supplementary file 7 — Source data Fig. 6 [file 44319_2026_766_MOESM7_ESM.zip › 6H/260109_Cal2KD&G3BP Mut, TuJ Axon regen.002_1. G3BP1 WT rescue003_Processed001_ch00.tif]

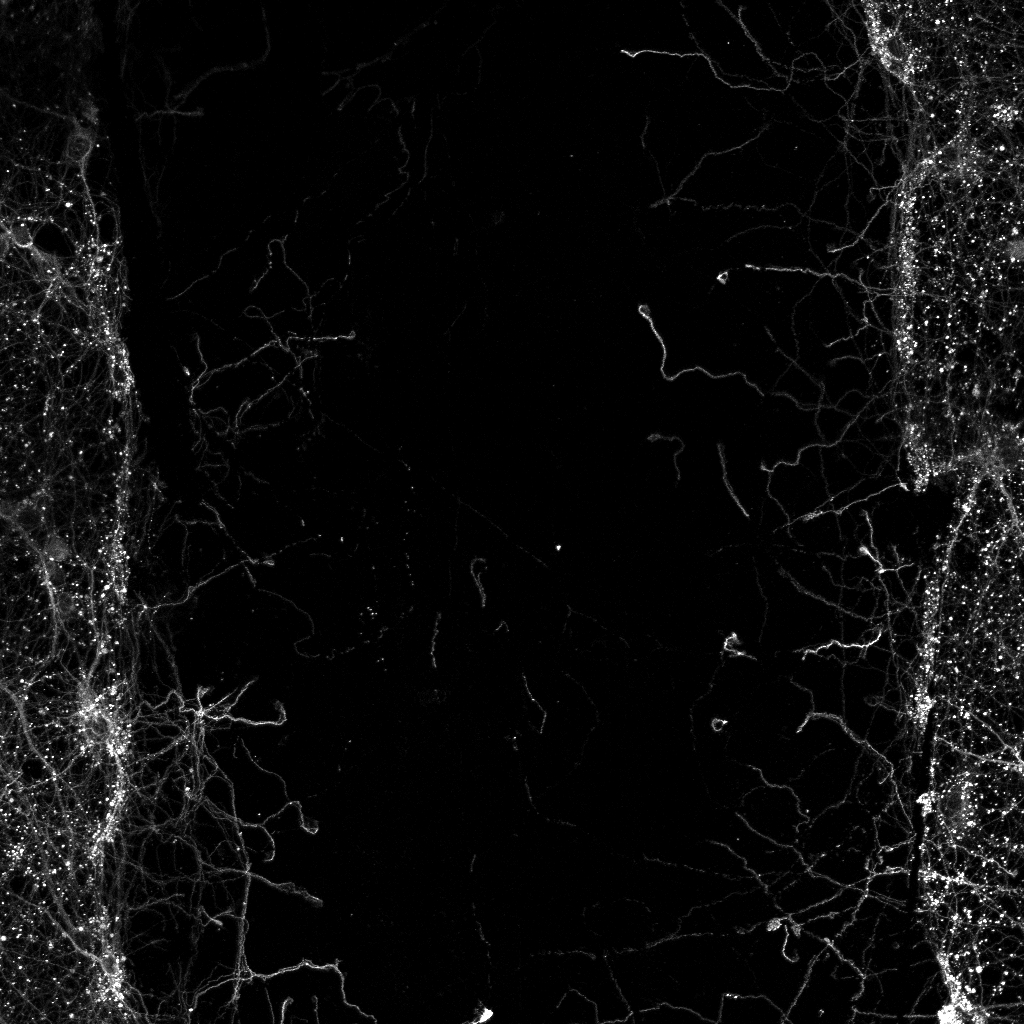

Supplement: Supplementary file 7 — Source data Fig. 6 [file 44319_2026_766_MOESM7_ESM.zip › 6H/260109_Cal2KD&G3BP Mut, TuJ Axon regen.002_2. G3BP1 Del_PxxP rescue001_Processed001_ch00.tif]

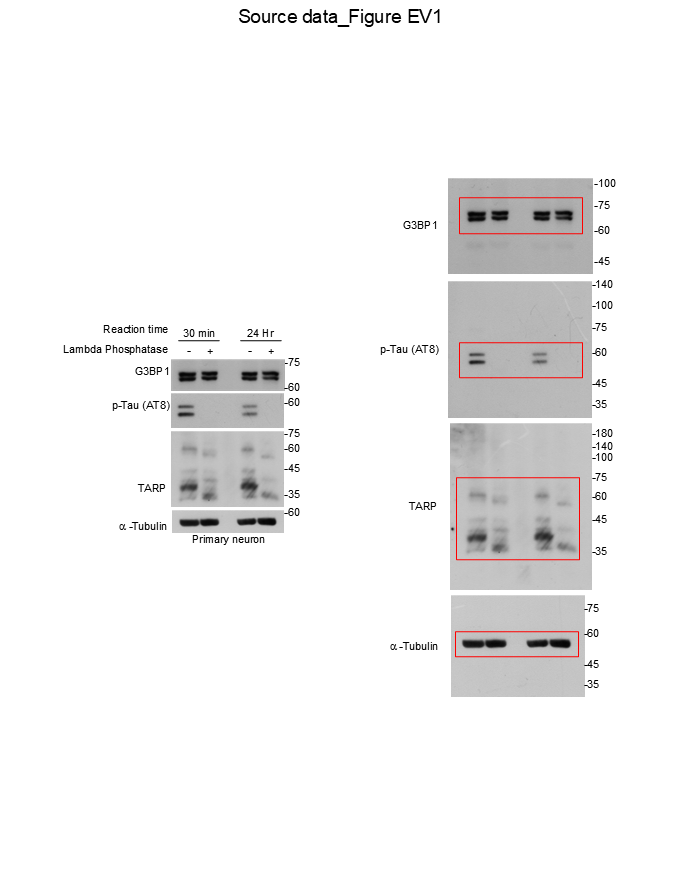

Supplement: Supplementary file 8 — Figure EV1 Source Data [file 44319_2026_766_MOESM8_ESM.zip › EV1/FigureEV1_Blots.TIF]

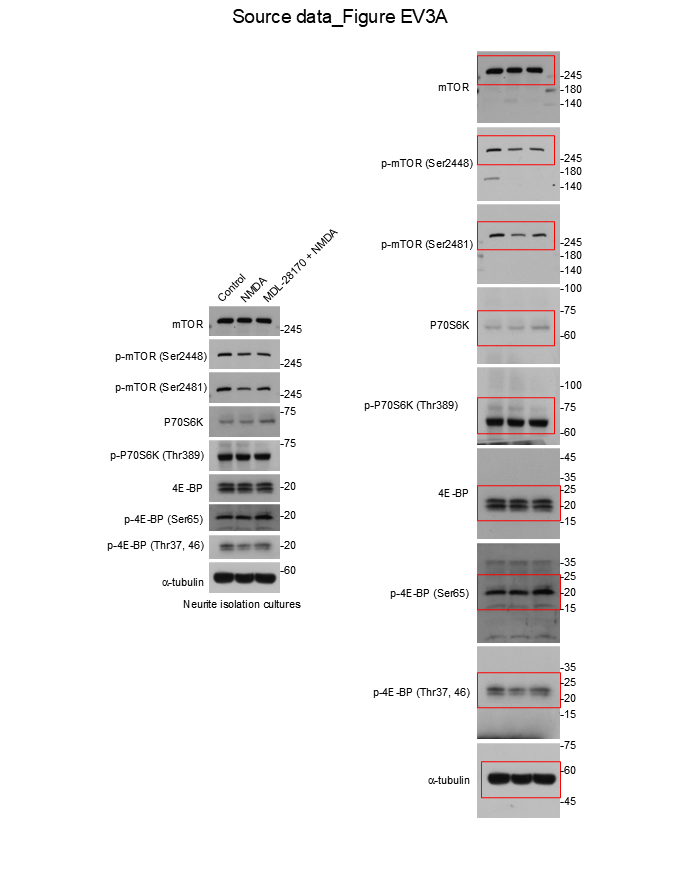

Supplement: Supplementary file 10 — Figure EV3 Source Data [file 44319_2026_766_MOESM10_ESM.zip › EV3A/FigureEV3A_Blots.TIF]

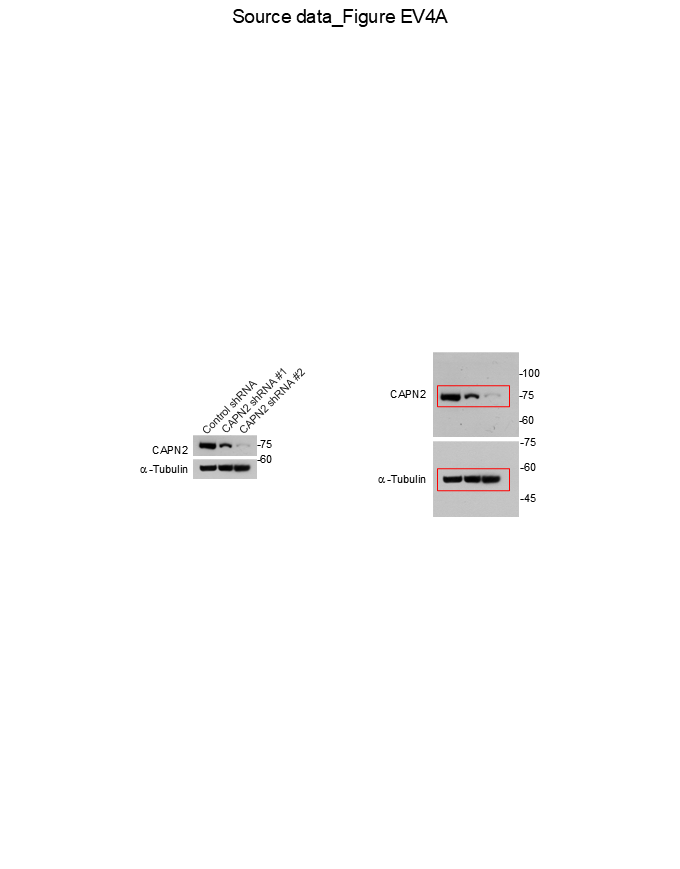

Supplement: Supplementary file 11 — Figure EV4 Source Data [file 44319_2026_766_MOESM11_ESM.zip › EV4A/FigureEV4A_Blots.TIF]

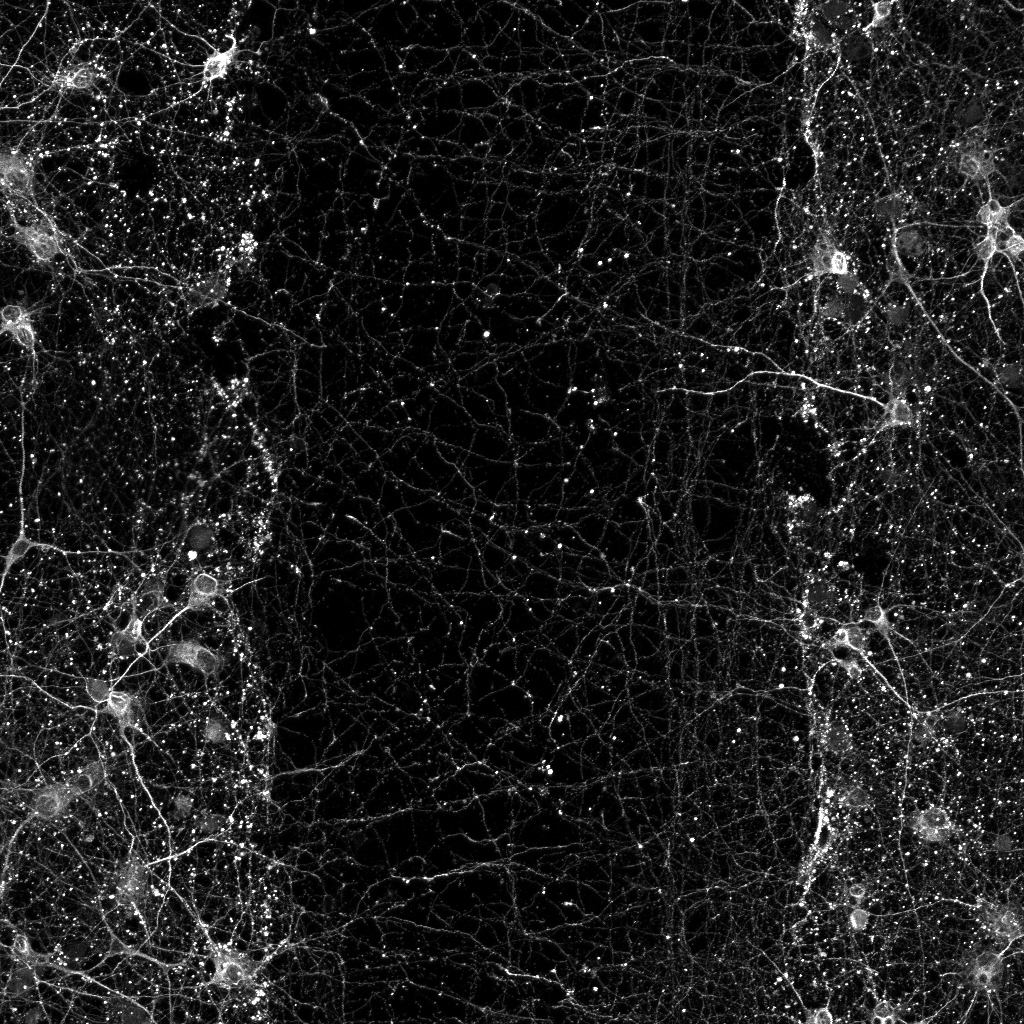

Supplement: Supplementary file 11 — Figure EV4 Source Data [file 44319_2026_766_MOESM11_ESM.zip › EV4B/260109_Cal2KD&G3BP Mut, TuJ Axon regen.001_1. ConKD005_Processed001_ch00.tif]

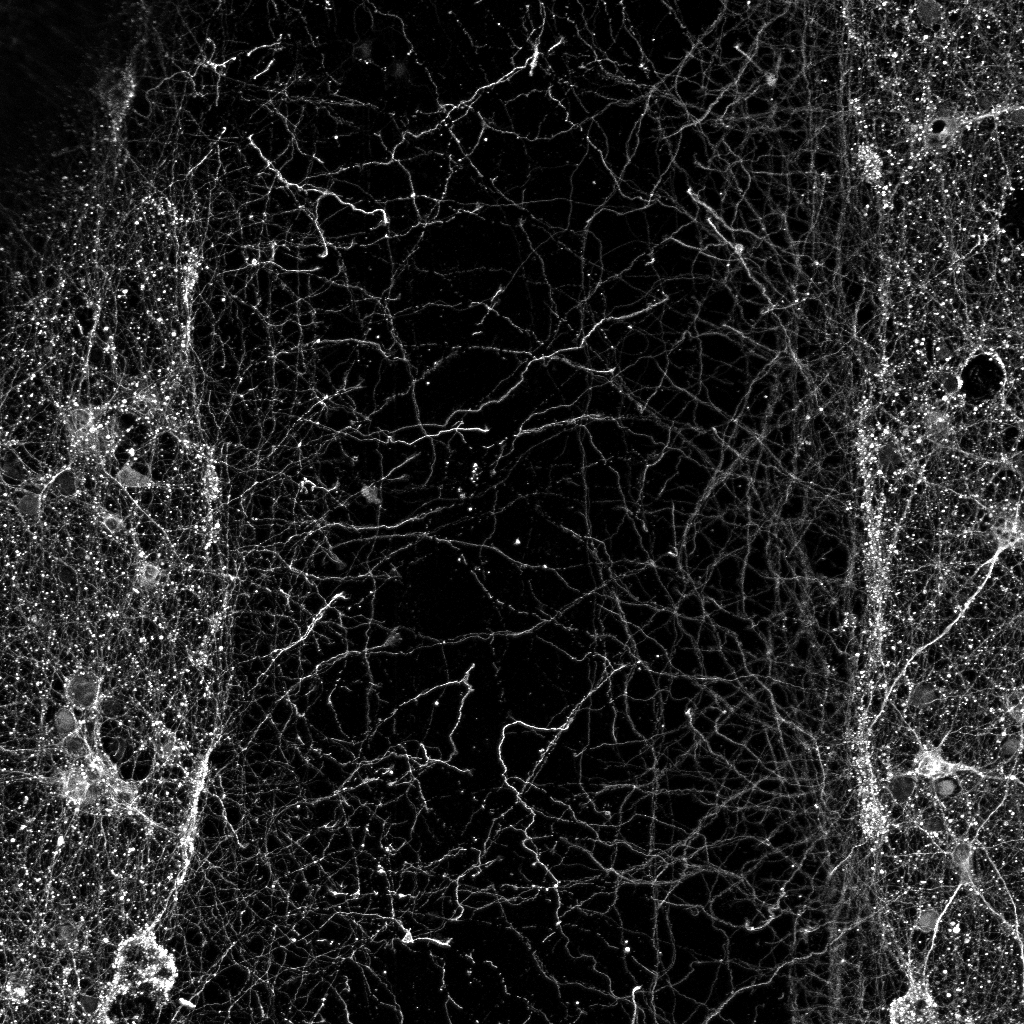

Supplement: Supplementary file 11 — Figure EV4 Source Data [file 44319_2026_766_MOESM11_ESM.zip › EV4B/260109_Cal2KD&G3BP Mut, TuJ Axon regen.001_2. CAPN2KD#1_001_Processed001_ch00.tif]

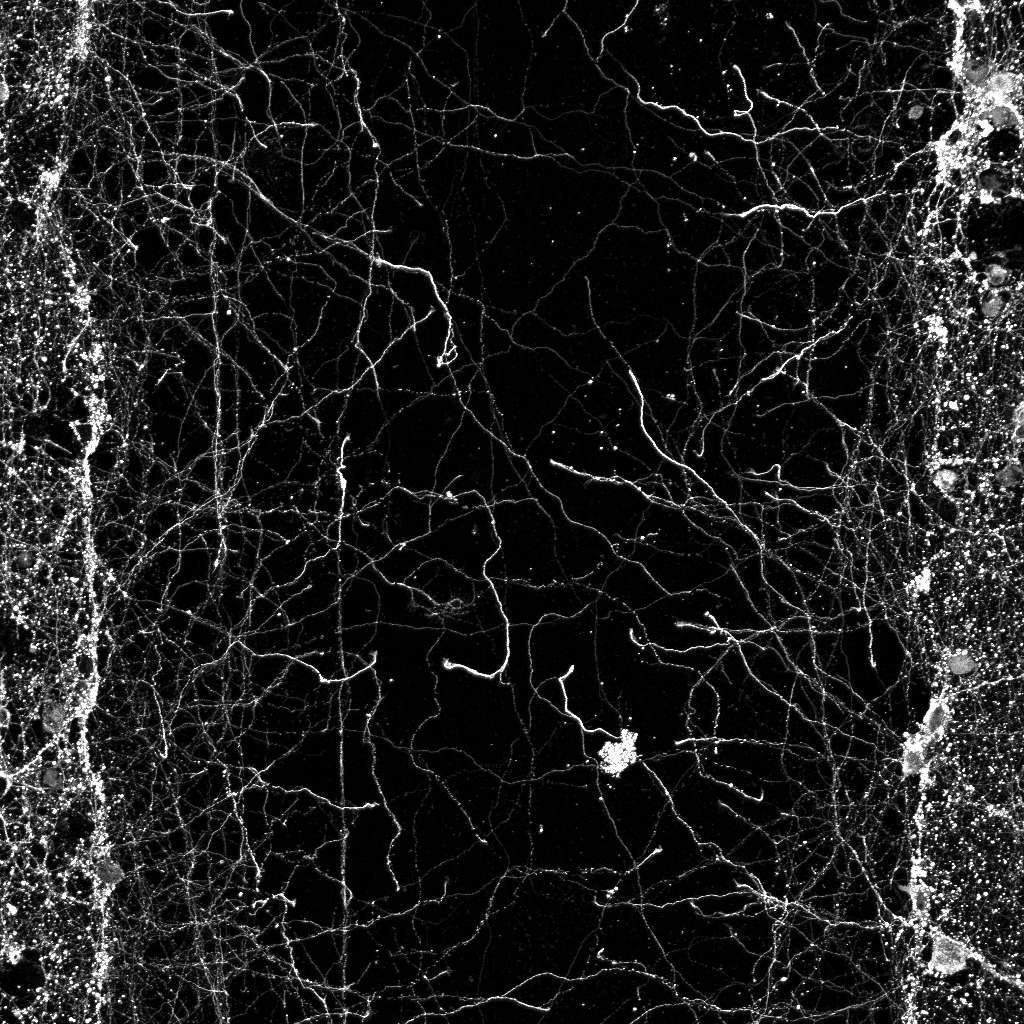

Supplement: Supplementary file 11 — Figure EV4 Source Data [file 44319_2026_766_MOESM11_ESM.zip › EV4B/260109_Cal2KD&G3BP Mut, TuJ Axon regen.001_3. CAPN2KD#2_001_Processed001_ch00.tif]

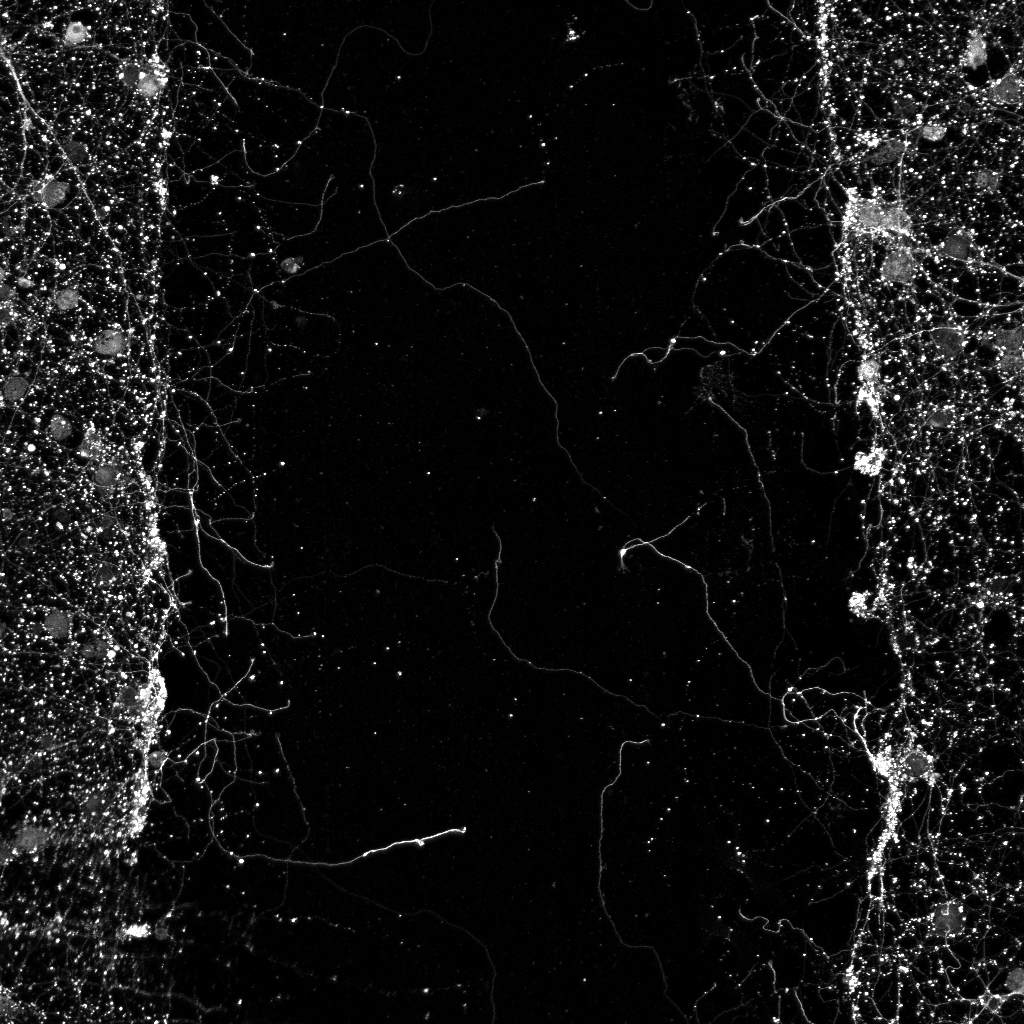

Supplement: Supplementary file 11 — Figure EV4 Source Data [file 44319_2026_766_MOESM11_ESM.zip › EV4B/260109_Cal2KD&G3BP Mut, TuJ Axon regen.001_4. ConKD+MDL004_Processed001_ch00.tif]

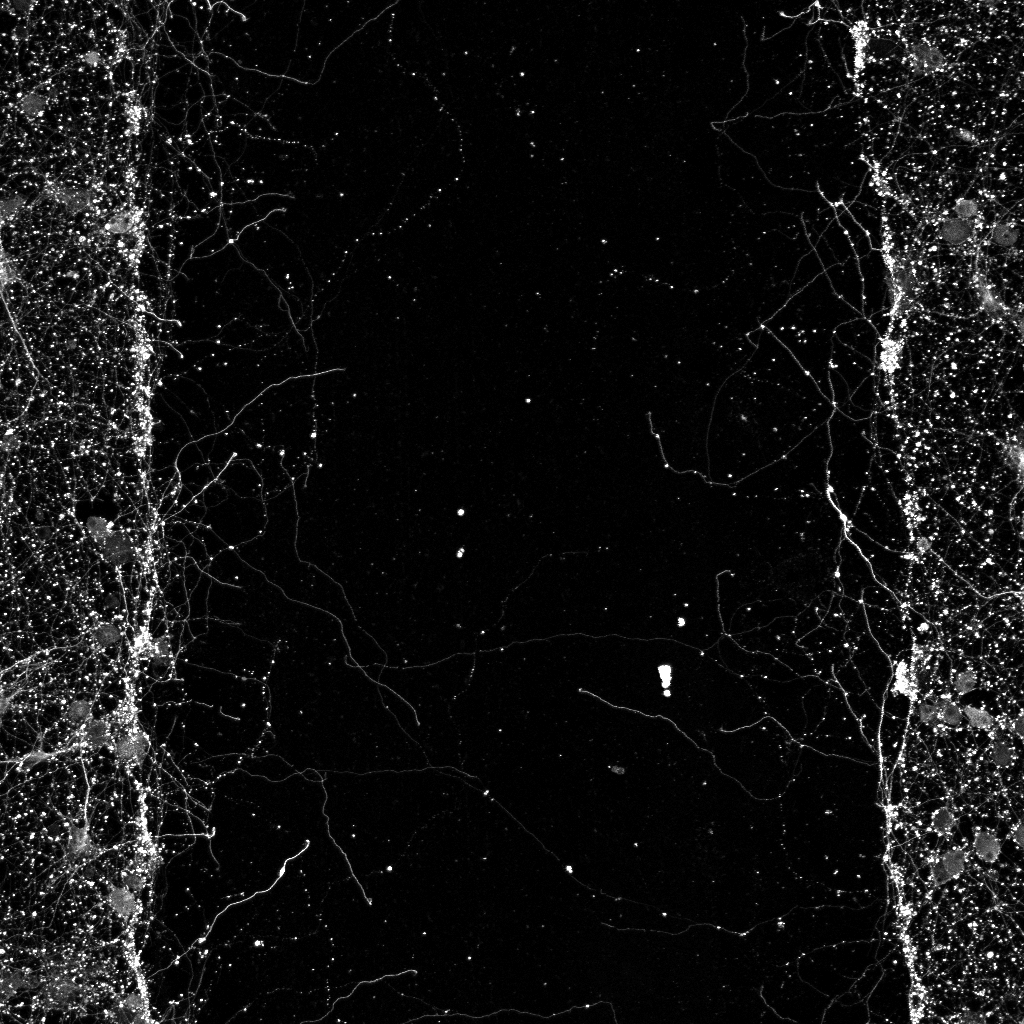

Supplement: Supplementary file 11 — Figure EV4 Source Data [file 44319_2026_766_MOESM11_ESM.zip › EV4B/260109_Cal2KD&G3BP Mut, TuJ Axon regen.001_5. CAPN2KD#1+MDL002_Processed001_ch00.tif]

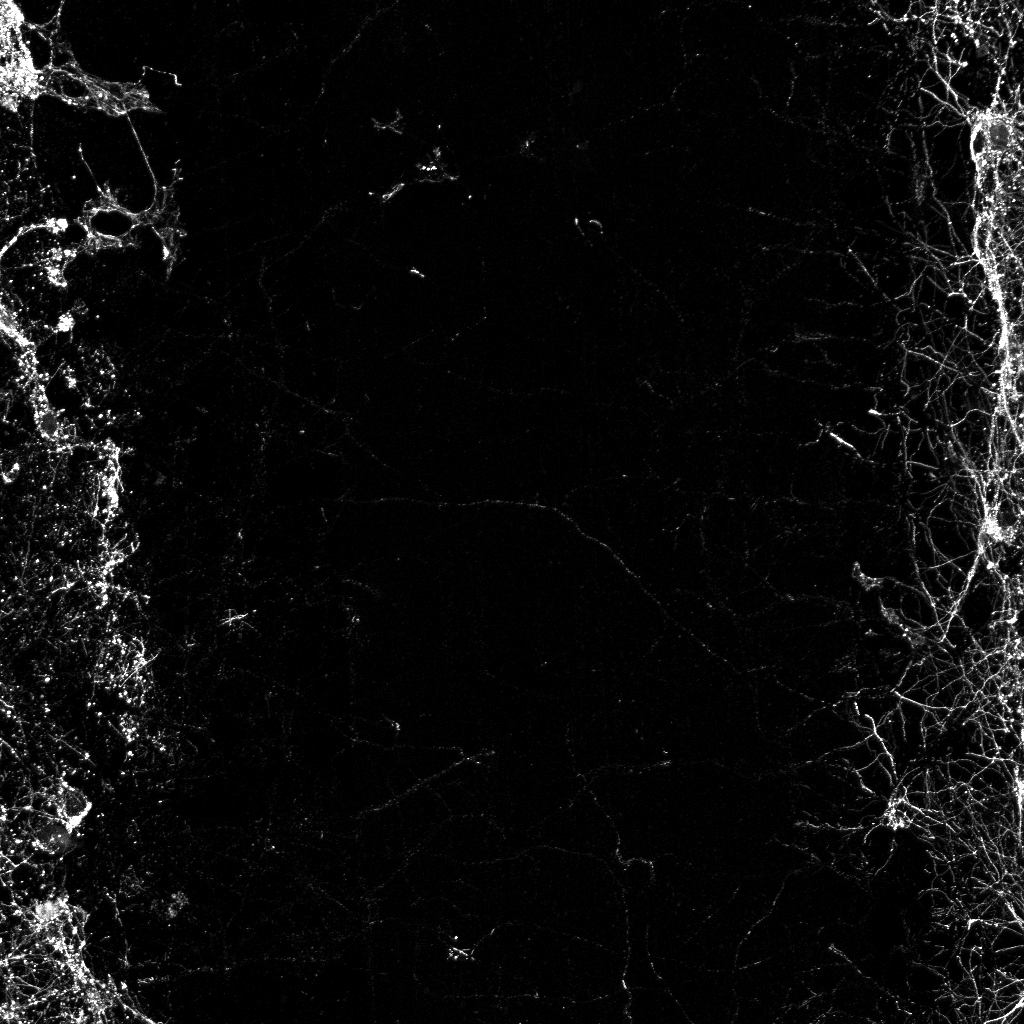

Supplement: Supplementary file 11 — Figure EV4 Source Data [file 44319_2026_766_MOESM11_ESM.zip › EV4B/260109_Cal2KD&G3BP Mut, TuJ Axon regen.001_6. CAPN2KD#2+MDL002_Processed001_ch00.tif]
