# Supplementary material for: Proteolytic cleavage of G3BP1 by calpain 1 couples NMDAR activation to mTOR-dependent local translation
Source: EMBO Rep. 2026 Apr 4;27(10):2749–71. doi: 10.1038/s44319-026-00766-9 (PMC13219515; doi:10.1038/s44319-026-00766-9)
Supplement: Supplementary file 12 — Expanded View Figures [file 44319_2026_766_MOESM12_ESM.pdf]

Expanded View Figures

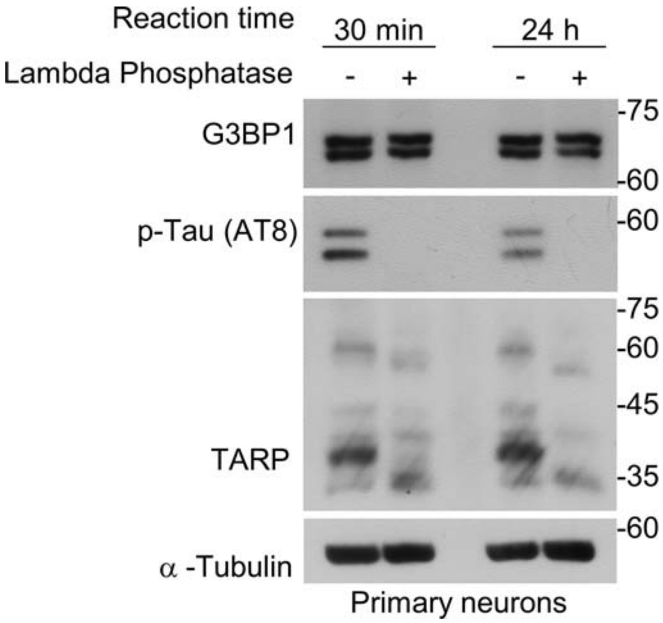

Figure EV1. The two bands of natïve G3BP1 are not due to phosphorylation-dependent post-translational modifications.

Western blot analysis of primary hippocampal neuron lysates treated with λ-phosphatase for 30 min or 24 h. The double band pattern of G3BP1 persists following phosphatase treatment, indicating that the upper band is not derived from phosphorylation-dependent post-translational modifications. Source data are available online for this figure.

 © The Author(s)

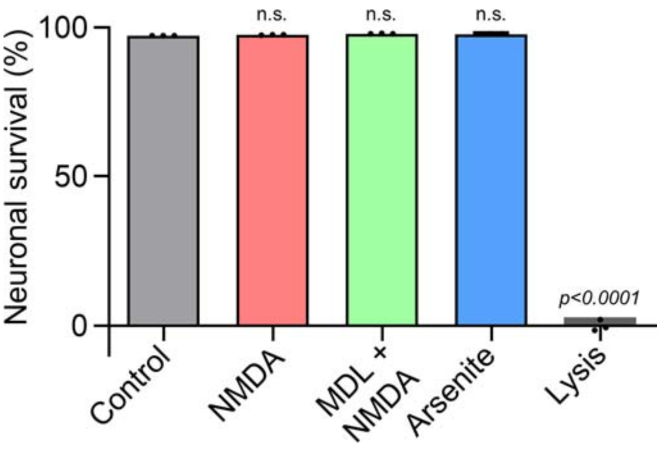

Figure EV2. Granule assembly or disassembly conditions do not significantly affect neuronal survival.

Neuronal survival was assessed by LDH cytotoxicity assay following 2-h treatments of DIV 14 primary hippocampal neurons. Conditions included granule disassembly (50 µM NMDA), prevention of disassembly (50 µM NMDA plus 5 µM MDL-28170), and granule assembly (200 µM sodium arsenite). No significant differences in cytotoxicity were observed among the conditions. Data are shown as mean ± SEM ($n = 3$, n.s., $P = 0.9910$ for NMDA, $P = 0.8722$ for MDL + NMDA, and $P = 0.9202$ for arsenite; one-way ANOVA with Tukey's post hoc test). Source data are available online for this figure.

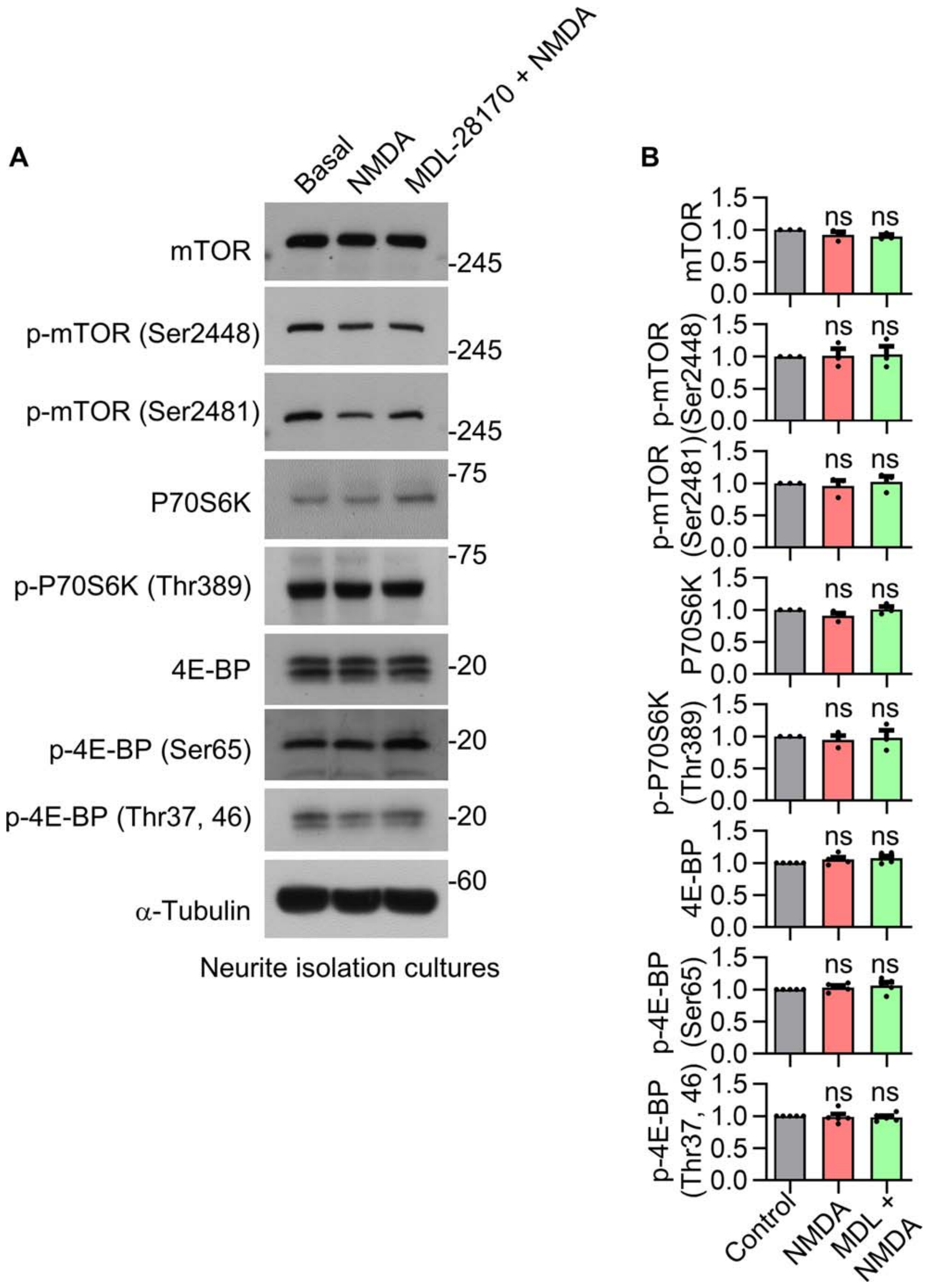
A
Basal
NMDA
MDL-28170 + NMDA
mTOR
p-mTOR (Ser2448)
p-mTOR (Ser2481)
P70S6K
p-P70S6K (Thr389)
4E-BP
p-4E-BP (Ser65)
p-4E-BP (Thr37, 46)
α-Tubulin
-245
-245
-245
-75
-75
-20
-20
-20
-60
Neurite isolation cultures
B
ns
Control
NMDA
MDL + NMDA

Figure EV3. Western blot analysis does not detect NMDA-induced changes in mTOR activity, even in neurite-enriched fractions.

(**A**) Western blot analysis of mTOR downstream signaling in neurite-enriched fractions of primary hippocampal neurons. (**B**) Quantification of mTOR downstream signaling from (**A**) is shown as mean ± SEM ($n = 3$–5, n.s., $P > 0.05$; one-way ANOVA with Tukey's post hoc test). For exact P values, please refer to the Source Data. Source data are available online for this figure.

© The Author(s)

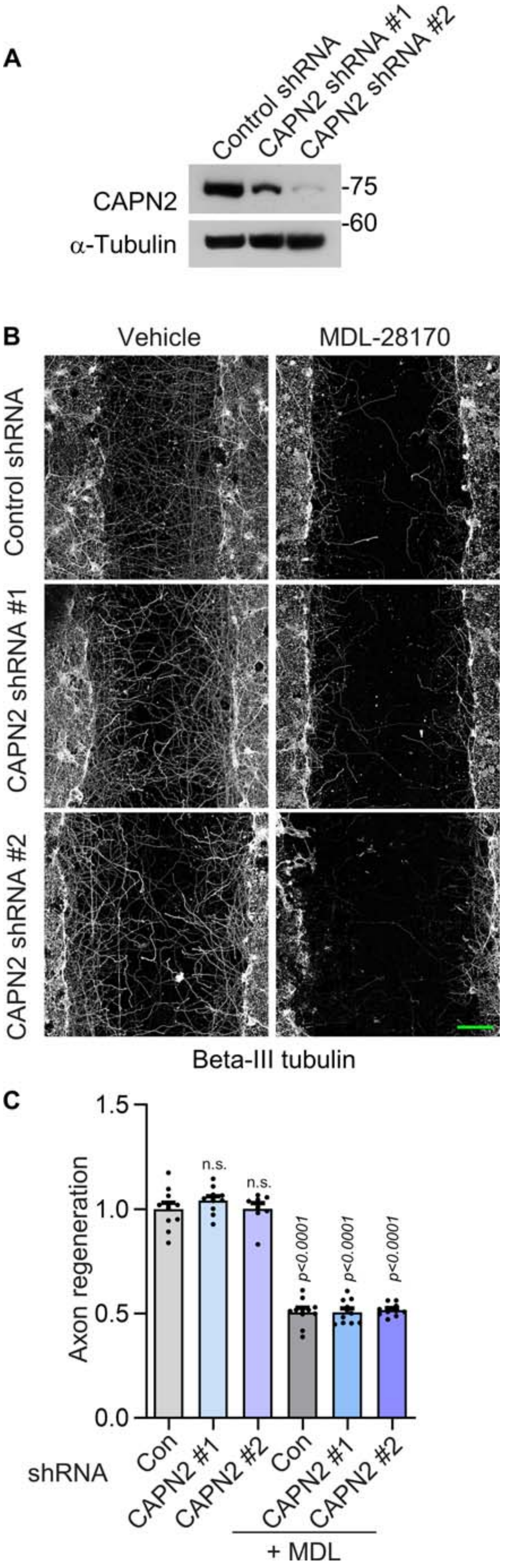
A
Control shRNA
CAPN2 shRNA #1
CAPN2 shRNA #2
CAPN2
α-Tubulin
-75
-60
B
Vehicle
MDL-28170
Control shRNA
CAPN2 shRNA #1
CAPN2 shRNA #2
Beta-III tubulin
C
Axon regeneration
1.5
1.0
0.5
0.0
n.s.
n.s.
p<0.0001
p<0.0001
p<0.0001
shRNA
Con
CAPN2 #1
CAPN2 #2
Con
CAPN2 #1
CAPN2 #2
+ MDL

 © The Author(s)

Figure EV4. Calpain 2 does not affect axonal regeneration.

(**A**) Western blot showing the knockdown efficiency of calpain 2 (CAPN2) using shRNA in primary hippocampal neurons. (**B**) Representative confocal images showing axonal regeneration visualized by beta-III tubulin immunostaining in neurons expressing CAPN2 shRNA or treated with MDL-28170. Scale bar, 100 µm. (**C**) Quantification of axon regeneration from (**B**) is shown as mean ± SEM ($n = 8$–10, n.s., $P = 0.7330$ CAPN2 #1 and $P > 0.9999$ for CAPN2 #2; one-way ANOVA with Tukey's post hoc test). Source data are available online for this figure.

© The Author(s)
